# Patient reported experience measures on HIV viral load testing at public health facilities in Dar es Salaam, Tanzania: A convergent mixed method study

**DOI:** 10.1371/journal.pgph.0001024

**Published:** 2023-04-07

**Authors:** Peter M. Karoli, Grace A. Shayo, Elizabeth H. Shayo, Christine V. Wood, Theresia A. Ottaru, Claudia A. Hawkins, Erasto V. Mbugi, Sokoine L. Kivuyo, Sayoki G. Mfinanga, Sylvia F. Kaaya, Eric J. Mgina, Lisa R. Hirschhorn

**Affiliations:** 1 Feinberg School of Medicine, Northwestern University, Chicago, Illinois, United States of America; 2 Muhimbili University of Health and Allied Sciences, Dar es salaam, Tanzania; 3 National Institute for Medical Research, Dar es salaam, Tanzania; Centre hospitalier de Cayenne, FRANCE

## Abstract

While viral load (VL) testing is critical to effective treatment of human immunodeficiency virus (HIV), little is known about patients’ experiences with, and barriers to VL-testing in the context of HIV infection. We assessed patient reported experience measures (PREMs) on VL-testing in public HIV clinics in Tanzania. In a cross-sectional convergent mixed method study, we collected information on VL test related PREMs, clinical and sociodemographic factors. PREMs were measured using a 5-point Likert scale. Focus Group Discussions (FGDs) explored on experience, access, and barriers to VL-testing. Descriptive statistics summarized patients’ factors and PREMs. Logistic regression was used to explore association of patient factors, PREMs and satisfaction with VL-testing services. Thematic analysis was used for qualitative data. A total of 439 (96.48%) respondents completed the survey, 331 (75.40%) were female, median (IQR) age was 41(34, 49) years. A total of 253(57.63%) had a VL test at least once in the past 12 months, of whom 242(96.0%) had VL<1000 copies/ml. Investigating barriers to VL-testing, most participants (>92.0%) reported good or very good health services responsiveness (HSR). A scale of very good was chosen by the majority for being treated with respect 174(39.6%), listened to 173(39.4%), following advice 109(24.8%), being involved in decisions 101(23.0%), and for communication 102(23.3%). Satisfaction on VL-testing services was significantly associated with respondents following care providers’ advice, (aOR) = 2.07 [95%CI 1.13–3.78], involvement in decisions aOR = 4.16 [95%CI 2.26–7.66], and communication aOR = 2.27 [95%CI 1.25–4.14]. FGDs findings converged with the survey data, with identified barriers to VL test including lack of autonomy in decision making, little awareness on the benefits of the test, long waiting time, stigma, competing priorities for those with comorbidities and transport costs. Satisfaction on VL-testing was largely a result of involvement in decision making, following care provider’s advice and good communication; entities needing universal improvement across the country.

## Introduction

In HIV care and treatment, VL testing is critical in informing clinical decision.[1]. Consistent testing of VL ensures timely monitoring of treatment adherence and efficacy as well as timely diagnosis and management of treatment failure in those on ART [2]. It serves as a better indicator for treatment response or failure than CD4 counts or clinical assessment [1]. Routine VL monitoring, improves the quality of HIV management and better patients treatment outcomes because, VL suppression is critical to achieve both reduction in mortality and morbidity as well as reducing HIV transmission [3] Regrettably, only 50% of the known people living with HIV (PLHIV) are estimated to be able to access the VL test services in both lower and middle income countries (LMICs) [4]. A study in Cameroon reported that only 24.3% of patients in care had at least one VL test between 2013 to 2017 and most were conducted more than 24 months since initiation of ART [5]. However higher coverage is possible with studies reporting higher rates of VL testing, such as rural Uganda which reported 66% of patients having a VL test in 2017 [6] however, in between 2013 to 2018, Uganda had 89% of patients who had at least one VL test within those five years [7]. The most recent published data indicates that, within the past five years (2013 to 2018) the proportion of having at least one VL test in sub-Saharan region ranged from 51% to 99%, Namibia having the highest rates of testing [7]. Unfortunately, Tanzania was not included in this analysis due to incomplete data [7].Tanzania has an estimated 1.4 million people living with HIV (PLHIV), with prevalence of around 6.3% female and 3.9% men [8]. In 2015, Tanzania adopted WHO recommendations of testing all patients in care at 6 months, then 12 months after initiation of antiretrovirals therapy (ART), and yearly thereafter for life [9]. The country scaled up the HIV viral load testing in 2016 by establishing zonal and regional laboratory capacity [9]. However, according to published data from 2018 to 2019, the country VL testing rates are below 22% among HIV patients who meet the criteria of testing as per national HIV VL management guidelines [10, 11]. These guidelines stipulate that all patients initiating antiretrovirals therapy (ART) should do VL testing at 6 months after initiation of ART, then once every year for life [9].

Understanding barriers to VL testing is important in improving quality of care and achieve the goals to end the HIV epidemic. System and structural barriers for VL testing in LMICs have been described. These include long turnaround time from specimen collection to results, lack of sufficient number of well-trained laboratory staff, and lack of awareness among clinicians on the importance of ordering and testing [2, 12]. Other barriers include inefficient specimen transportation lack of knowledge among laboratory workers about VL testing, reagent procurement delays and inadequate financial resources to support the HIV-viral load testing scale-up [2, 9, 12].

Although much of the barriers have been unearthed in previous studies, it is important to also understand the experiences of VL testing and explore barriers to VL uptake from the patient’s perspective. Building on work by the World Health Organization in Health System Responsiveness [13], patient reported experience measures (PREMs) are metrics that captures the interaction during an episode of care, and explain what happened from the perspective of the patient [13]. These measures have gained worldwide recognition as an important indicator of quality and people-centeredness of the health care services [13]. Results from studies in Kenya and Namibia have shown that decisions about whether to attend scheduled visits for care are influenced by experiences of care during previous interactions with the facility providers [14]. PREMs have also been associated with better HIV adherence and retention in Dar es Salaam, but little is known about patient experience on viral load (VL) testing and how it influences treatment outcomes beyond system and structural barriers [15].

This study was designed to measure patients’ experiences in the receipt of viral load testing according to national protocol in public health facilities in Tanzania and explored individual and structural barriers to accessing VL testing. The results are important to inform health care providers and other stakeholders on measures to improve the quality and rates of viral load testing.

## Materials and methods

### Conceptual framework

The King’s theory of goal attainment during health care provider-patient interaction was chosen to inform the design of this study and to learn from patients experience on VL testing services in public health facilities in Dar es Salaam. The theory describes patient/care provider interactions as the key process to ensure service is provided to attain the intended treatment goal. The framework explains that the health care provider has to take actions and set goals with the patient, apply effective chain of communication to interact with the patient which ends up with transaction and goal achievements [16–18]. The theory highlights how both the provider’s and the patient’s perception, expectations and communication are needed to achieve a desired outcome [16–18].

### Study design and setting

Between October 2021 and January 2022, we conducted a convergent mixed methods cross-sectional study employing a patient survey and focused group discussions (FGDs) with the patients in 6 public HIV clinics in Dar es Salaam, Tanzania. The data collected by the survey included clinical and sociodemographic factors and PREMs. FGDs were designed to further understand patients’ experience with viral load testing including facilitators and barriers to VL testing within and beyond usual structural and system challenges. All the facilities involved in the study were owned by the government of Tanzania and were under the support of a non-governmental organization namely Management and Development for Health (MDH) [19]. MDH objectives include working with the government to support HIV clinics and the public health in general using evidence-based and the innovative interventions for the benefit of people living with HIV [19].

### Population, sampling and selection criteria

At the conception of this study in 2020, there were 108 public, non-military owned health facilities in Dar es salaam. These included 5 hospitals, 16 health centers and 87 dispensaries. These health facilities were grouped in strata defined by level of care (hospital, health center, and dispensary level). From each strata two facilities were randomly selected using a random selection table [20]. Participants of the study were adult PLHIV aged 18years or older, attending HIV clinic in the 6 selected public health facilities. To be enrolled into the study, consenting participants needed to have been in care for at least a year before the commencement of the study.

### Data source and data collection

#### Quantitative data

Clinic-based survey was completed in a face-to-face approach following informed consent. A convenient sample of both men and women adult PLHIV attending in the 6 study sites were randomly invited to participate in the PREMs survey. These participants were obtained on their usual appointments to the clinics. In this study, a minimum sample size of 440 participants was calculated using the Cochran formula [9, 21]. A prevalence of 22% of accessing HIV VL testing [10, 11] was used, assuming 10% non-response rate and a 1.5 design effect. Socio-demographics including sex, age and education level were also collected. PREMs were measured using a survey which had been developed based on the WHO HSR measures and already used in the HIV care studies in Tanzania which had been translated in Swahili and undergone cognitive debriefing and validation [15, 22]. Additional questions were added with reference to another similar study measuring HSR in Ghana [23]. PREMS were measured using a 5-point Likert scale where patients were asked to rate the VL services using indicators including respect, being listened to, autonomy, advice, communication, turnaround time, and health system quality outcomes including satisfaction on how VL services received met their treatment goals (Table 2). Following the survey, we also reviewed the facility-based patient care and treatment card to record the VL test date and results in the past 12 months.

#### Qualitative

We also conducted three focused group discussions (FGDs) with the participants, one at each of the 3 strata of facilities to learn from the patient’s experiences beyond the survey data. The number of FGDs was limited due to time and resources. The interview guide questions were developed based on the King’s theory of goal attainment [16–18], designed to understand patient’s experiences of care, facilitators and barriers to obtaining VL testing and in receiving back the results. The intention of doing FGDs was to explore its agreement with the survey data.

FGDs were conducted in Swahili language. For each of the FGD, we used a convenience sampling to invite 9–12 adult PLHIV. Patients were invited to the discussion after receiving their health care services while exiting the health facilities. Informed consent was obtained for each participant. Each FGD had more female representatives to at least 60% to reflect the distribution of the HIV population in Tanzania. Participants of the FGD included at least one expert patient (a peer educator at their respective clinic). To ensure auditory confidentiality and privacy, FGDs were conducted in unused room or outdoor environment (behind the HIV building within the fenced health facilities). Each participant was given a chance to discuss and the expert patient wasn’t dominant. Audio recording was done and extra notes including patients’ characteristics were documented. The FGDs were conducted by PMK assisted by a social scientist (ZAK) with a vast experience in doing qualitative studies for more than 7 years from the National Institute for Medical Research Tanzania.

## Quantitative variables

The outcome variables in the survey data was satisfaction with VL testing services defined as meeting patient’s VL healthcare goals rated as very good. Independent variables included patients’ demographic information (age, sex, level of education), VL testing, VL suppression, and the PREMs.

### Data analysis

#### Quantitative analysis

Descriptive analysis for patients’ demographics, clinical characteristics, VL testing and PREMs were carried out using Stata software version 14. 2. PREMs indicators were further dichotomized from a 5-points Likert scale to very good versus other responses for the regression analysis. Univariate and multivariate logistic regression were computed to measure the association of patient’s characteristics and PREMs to the overall VL testing services satisfaction. Only variables with a p-value of ≤0.25 in univariate model was entered in a multivariate model. However, gender (sex) variable with p value >0.307 at the univariate model was included in the multivariate model because in some of the literature (published data), females are more likely to report being satisfied with the health care services [24–26]. Significance in multivariate model was set at P value of ≤0.05.

#### Qualitative analysis

For the focused group discussion, recordings were transcribed, and translated into English with an emphasis on retaining culturally embedded expressions. During analysis, PMK read all the transcripts and listened to the audios carefully to confirm the correctness of the materials. An initial code book was developed based on the King’s theory of goal attainment [16–18] where, patient-care provider communication and interactions served as a backbone for the codes. PMK and a second independent coder (TAO), coded the FGDs transcripts using an excel spread sheet. PMK and TAO met to compare and refine the codebook. Analysis included sorting and clustering the codes into themes addressing the key research questions. Quotes were extracted and presented to reflect the informant’s own words in a narrative report. FGDs findings served as supplementary information to provide insight into survey data on understanding the existing gaps in VL testing services.

#### Ethics statement

Ethical clearance was obtained from Institutional Review Bodies of the Muhimbili University of health and allied sciences [IRB No. DA.282/298/01.C], The Medical Research Coordinating Committee of the National Institute for Medical Research (NIMR) in Tanzania [IRB No. NIMR/HQ/R.8a/Vol.IX/3902] and from The Northwestern University ethical board in the USA [IRB No. STU00215874]. We obtained permission to conduct the study from the authorities in Dar es salaam city and their respective districts and health facilities. Moreover, we obtained written informed consent from the study participants. We adhered to the anonymity of the participant’s information, and we collected only the participant’s data related to the study. Participants’ information in electronic format was password protected and was only accessed by authorized study team individuals. In all stages of this research, compliance to all ethical boards’ terms and conditions were observed.

## Results

### Quantitative results

#### Participants’ sociodemographic and clinical characteristics

Of 455 patients invited to participate, 439(96.5%) completed the survey. All non-respondents asked not to participate because they were in a rush. A total of 331 (75.4%) respondents were female. The median age (IQR) was 41 (34,49) years. Most participants (80.2%) had primary level education with only 19.8% having attained a secondary school or higher education. About 253 (57.6%) participants had a VL test at least once in the past 12 months, with almost all tested 242/253 (96.0%) having viral suppression defined as HIV viral load below 1000 copies/ml. Therefore, 42.4% of the participants who are eligible for a VL test, did not perform the test as per the available HIV viral load guidelines (Table 1).

**Table 1:** Patients demographics and clinical characteristics, N = 439.

Variable		Number	%
**Sex**			
	Male	108	24.6
	Female	331	75.4
**Age**			
	18–30 years	78	17.8
	31–40 years	126	28.7
	41–50 years	136	31.0
	Above 50 years	99	22.6
**Education**			
	Completed Primary or none	352	80.2
	Completed Secondary or higher	87	19.8
**Facility Level**			
	Hospital	199	45.3
	Health Centers	164	37.4
	Dispensary	76	17.3
**VL testing**			
	VL tested in last 12 months	253	57.63
	HIV viral suppression (VL< 1000 copies/ml) (among those tested in last 12 months)	242	96.03

#### Patients reported experience measures

While the majority (> 92%) rated experiences as good or very good, only 20% to 40% rated PREMs as very good, including being treated with respect (39.6%), being listened to (39.4%), and easiness in following provider’s advice (24.8%), being involved in making decisions (autonomy) (23.0%), and providers explaining things clearly including VL testing (communication) (23.2%). The turnaround time for VL test results was rated very good by fewer respondents (19.1%), compared to other domains (Table 2).

**Table 2:** Patients reported experience measures (PREMs), N = 439.

Patient experience measures (PREMs)		Number	%
How would you rate your experience of getting treated with **respect** today? Respect means being treated politely and fairly			
	Very Good	174	39.6
	Good	234	53.3
	Moderate	26	5.9
	Bad	5	1.1
	Very bad	0	0.0
How well did doctors and nurses or other health care providers **listen** to you?			
	Very Good	173	39.4
	Good	241	54.9
	Moderate	23	5.2
	Bad	2	0.5
	Very bad	0	0.0
How easy or difficult was it for you to follow the provider’s **advice** for you to test for HIV VL and managing your illness when results were shared?			
	Very Good	109	24.8
	Good	309	70.4
	Moderate	13	3.0
	Bad	2	0.4
	Very bad	6	1.4
During the last time you tested for VL, how would you rate your experience of being involved in **making decisions** for VL testing? (autonomy)			
	Very Good	101	23.0
	Good	301	68.6
	Moderate	32	7.3
	Bad	4	0.9
	Very bad	1	0.2
During the last VL test and results sharing, how would you rate how **clearly** health care providers **explained things including VL test** to you? (communication)			
	Very Good	102	23.3
	Good	310	70.6
	Moderate	26	5.9
	Bad	1	0.2
	Very bad	0	0.00
How would you rate the **turnaround time** of the VL test at the facility before you received back the VL results? That means, the number of days you waited for the results after sample collection? (timeliness)			
	Very Good	84	19.1
	Good	294	67.0
	Moderate	49	11.2
	Bad	11	2.5
	Very bad	1	0.2
Overall, thinking about your entire last VL test visit, please rate how well the VL service you received **met your health goals**. That is, how much did the VL test and results help solve your treatment failure or help you keep on good treatment adherence? (satisfaction)			
	Very Good	131	29.8
	Good	282	64.2
	Moderate	22	5.1
	Bad	3	0.7
	Very bad	1	0.2
Overall, taking everything into account, how would you rate the **quality of VL test service** at this facility? (quality)			
	Very Good	130	29.6
	Good	289	65.8
	Moderate	18	4.2
	Bad	1	0.2
	Very bad	1	0.2

About one third (131, 29.8%) of respondents reported that the VL service received met their health goals, with similar percent 130 (29.6%) reporting that the quality of VL testing service at their respective health facility was very good (Table 2).

#### Logistic regression of barriers or facilitators of VL testing

In multivariate analysis, being able to follow care providers’ advice, adjusted odds ratio (aOR) = 2.07 [95%CI: 1.13–3.78], being involved in making decisions (autonomy) on VL testing aOR = 4.16 [95%CI: 2.26–7.66], and clear explanations (communications) on VL testing from health care providers aOR = 2.27 [95%CIL1.25–4.14], were significantly associated with high rating of VL testing services meeting participants health goals. The univariate model findings indicates that, getting treated with respect and being listened by their care providers were significantly associated with high rating of VL testing services meeting participants health goals; however, when these variables were adjusted with other factors, getting treated with respect aOR = 1.77 [95%CI: 0.96–3.26], and being listened aOR = 1.59 [95%CI: 0.85–2.96], were statistically not significant associated with the high rating of the services (Table 3).

**Table 3:** Logistic regression of association of patient factors and PREMs with VL testing meeting participants’ health goals.

Measure		Univariate			Multivariate		
**Sex (Ref: Male)**		OR	95%CI	P value	OR	95%CI	P value
	Female	1.29	0.79–2.11	0.307	1.64	0.90–3.00	0.105
**Age (Ref: 31–40)**							
	18–30 years	0.55	0.27–1.10	0.089	0.44	0.18–1.06	0.066
	41–50 years	1.32	0.78–2.23	0.299	1.07	0.57–2.00	0.843
	Above 50 years	1.31	0.74–2.31	0.354	1.06	0.53–2.10	0.879
**Education** (Ref: Primary or lower)							
	Secondary or higher	0.70	0.41–1.20	0.196	0.80	0.39–1.63	0.538
**Facility Level** (Ref Health center)							
	Hospital	1.41	0.89–2.23	0.143	1.28	0.73–2.24	0.391
	Dispensary	1.34	0.74–2.44	0.336	1.23	0.58–2.60	0.586
**HIV viral load in last 12 months** (Ref: Not tested)							
	Tested	1.32	0.87–1.99	0.192	1.09	0.65–1.82	0.751
**Patient experience measures (PREMs)**							
**Respect** (Ref: Other)							
	Very Good	4.32	2.81–6.68	<0.001	1.77	0.96–3.26	0.069
**Listened to** (Ref: Other)							
	Very Good	3.99	2.60–6.14	<0.001	1.59	0.85–2.96	0.146
**Follow advice** (Ref: Other)							
	Very Good	5.91	3.70–9.44	<0.001	**2.07**	**1.13–3.78**	**0.019**
**Autonomy** [Involvement in decision] (Ref: Other)							
	Very Good	7.92	4.85–12.94	<0.001	4.16	**2.26–7.66**	**<0.001**
**Communication** [Clear explanations] (Ref: Other)							
	Very Good	6.79	4.19–10.99	<0.001	2.27	**1.24–4.14**	**0.007**

### Qualitative results

On the other hand, FGDs participants had a median age of 40(range 21–65) years. Of the 27 FGDs participants, 16 were female, 17 were single and 15 had a primary school level of education. Majority of patients had been in HIV care for more than 5 years (Table 4).

**Table 4:** Characteristics of the FGDs participants.

FGDs Participants Characteristics: N = 27	Number
Median age (range)	40
Sex	
Female	16
Male	11
Marital status	
Married	8
Single	17
Widower	2
Education level	
No school	1
Primary school	15
Secondary school	10
College or higher	1
Facility level	
Hospital level	9
Health center level	9
Dispensary level	9
Years in HIV care	
Below 2 years	1
2–5 years	3
6 years and above	23

Patients’ knowledge on and attitude towards VL testing.

Some of the participants of the FGDs reported good knowledge on VL testing. They knew the benefits of doing viral load testing including monitoring the progress of their disease.

The following quotes testify;

*“When someone says viral load*, *it means that there is a test that we are supposed to do in order to know the number of viruses*, *because sometimes we stay a whole year without accessing the test*.” FGD participant, 29 years old female, 4 years in care, hospital level.

*“About HIV viral load testing*? *well*, *when we use medication we are supposed to test so as to know if those medications are working*. *In the other words*, *the test is to see if a client is using ARVs correctly*, *like taking at the appropriate time*, *that is why we test HIV viral load”*. FGD participant, 48 years old male, 7years in care, dispensary level.

Some of the patients reported that a VL result showing undetectable VL was a motivation factor for them to adhere to the medication as instructed. They also explained that VL results do help clinicians determine if current ART should be continued or changed

*“This is very important because when you test you can know your treatment progress*, *because you will know whether the medication is working in your body or not*. *The good results give hope and trust to keep on using the ARVs when you see that the viruses have been suppressed and your CD4 has increased so you feel healthy like anyone else*.*”* FGD participant, 48 years old male, 7 years in care, dispensary level.

Some patients also reported that knowing the VL results particularly those of suppression levels motivated adherence to clinic appointments especially those appointments for taking the test thus they would be eagerly waiting for the results because of the learnt benefits.

*“Yes*, *viral load testing helps to know the progress of the patients since they started using ARVs*, *what amount of virus do they have*, *and if their health is progressing well or not*.*”* FGD participant, 30 years old female, 7 years in care, health center level facility.

#### Communication between care providers and patients in the VL test service delivery

Similar to results in the survey, as part of VL testing, communication was felt to be pivotal in meeting their health goal. These included receiving phone calls or text messages from care providers as reminders for them to attend the VL test visit as revealed in this quote;

*“Even yesterday*, *they sent me a text message to take care of my health which reminded me that I need to come to the clinic*. *The service providers are good and even if you forget they must call you and remind you of the services you are supposed to get”*. FGD participant, 24 years old Female, 6 years in care, health center level

However, at health center level facilities some patients reported to have received low attention and unsatisfactory communication from care providers concerning VL testing, reasons thought to be workload and shortage of staff;

*““when a doctor is taking your blood sample you may ask questions*. *Because there are many clients*, *he cannot listen to you attentively*. *So*, *we are not given enough time to be listened to”*. FGD participant, 30 years old female, 7 years in care, health center level.

To address these communication challenges and improve patient/care provider communication, participants advised a clear division of roles among care providers;

*“…They [care providers] must divide themselves*, *if one dispenses medication then he/she should only dispense and if the other one is receiving and registering clients then he/she should only be playing such a role*. *So*, *this is a challenge that really needs to be addressed because we have experienced it here [name of the facility hidden]”*. FGD participant, 47 years old female, 13years in care, health center level

#### Respectfulness

Results from the FGDs showed that participants reported being treated respectfully as their ratings on respectful treatment were good or very good.

*“Surely they [care providers] receive us well and provide good services although sometimes they are busy with the works they have*." FGD participant, 47 years old female, 13 years in care, health center level

A number of FGD participants in all facility levels did also described some occasions where they received unpleasant language from care providers which intimidated them and resulted in low confidence in continuing with care seeking in the clinic.

*“Sometimes you may arrive on time at the clinic and find the care provider who is supposed to take your sample is busy with her work*. *So*, *she can tell you after a long wait*, *‘Why don’t you seat there and wait’*. *Sometimes she is not doing anything but just seated*. *As a human being it hurts because you do not expect to get such an answer from a service provider”*. FGD participant, 37 years old female, 6 years in care, dispensary level

#### Services and timeframe for VL test

The qualitative findings on VL turnaround time were consistent with the survey results. Many patients who had been tested for VL were comfortable with the testing schedules but indicated longer time to receive their VL results as getting feedback of the VL results would be done in the next ART refill appointment which is often 1–6 months away depending on patient’s stability.

*“It depends on the date you are scheduled to come and take medication at the clinic*. *So*, *when you come on that date you will also find your results are ready*.*”* FGD participant, 28 years old male, 11 years in care, hospital level

However, some patients were unaware of how soon they should receive VL results after they had taken the test, reporting receiving them when they are in the clinic for drug refilling.

*“I don’t know when they [the results] are made available from the lab*. *But if you are given medication for three months*, *once you finish those medications and you come to the clinic for refilling that is the day you will be given your VL test results”*. FGD participant, 33 years old female, 6 years in care, dispensary level.

Patients commended the efforts made by the care providers in notifying patients through text messages to attend the facility for retesting in case of invalid VL results.

*“My sister said results are usually ready after two weeks*, *so if the results are invalid*, *they (care providers) do send a text message to the client like the one they sent to me*, *so that the client comes to the clinic early to redo the test rather than waiting until he/she comes on his/her next clinic appointment”*. FGD participant, 33 years old female, 7 years in care, health center level.

#### Patient-reported barriers to access the VL testing

Patients reported barriers to VL testing including missing appointment due to distance and transport costs, persistent HIV stigma resulting in reluctance to attend the nearby facility (something not preferred by many) for the fear of meeting someone familiar at the clinic who comes from the same catchment area as them. Patients reported that some healthcare providers insisted that patients do receive services in nearby facilities, reflecting lack of patient-centeredness and disrespect of preferences. This has caused some patients quitting clinic.

*“The transport cost might be a barrier to some of us*. *I once faced this challenge*, *of course*, *they [care providers] advise us to register at the nearby facility*, *when we don’t do so*, *sometimes they forcefully transfer us*. *But they forget that we fear stigma from the surrounding community in the nearby facility*. *A good example is me*, *I once lived just near Kiwalani facility*, *the CTC is at the open space*, *if I go there*, *everyone knows me*, *now can I really take medication there*?*”* FGD participant, 46 years old female, 10 years in care, hospital level.

Competing priorities was also reported by patients with co-morbidities who had to attend multiple clinics for their treatments. These appointments conflicted with VL testing appointments sometimes resulting in missed appointment.

*“There is also a challenge for some clients having multiple clinics to attend at different service points*. *For example*, *a client may be receiving services at Magomeni for HIV and he is also receiving services at Mwananyamala hospital for hypertension*. *So*, *you can find that on the date that he is supposed to give a sample at Magomeni hospital is the same date that he is supposed to receive a service at Mwananyamala hospital*.*”* FGD participant, 24 years old, expert patient, female, 6 years in care, health center level

#### Lack of awareness

In contrast to the generally positive ratings for communication, patients who had shorter duration in care, and those who had never had a VL test were not aware of the VL test such that they could not tell the benefit of doing such a test as revealed in the following quote;

*“I am not very experienced*, *but I was told there is a test I am supposed to take and today when I came to the clinic*, *they took my blood sample*.*” “No*, *I have not been made aware yet*, *I was told that when I come today there is a test that will be done”* FGD participant, 44 years old Male, 1year in care, health center level.

#### Long waiting times

Patients also reported that long wait at the clinic was attributed by the shortage of staff which hindered timely VL testing.

*“For example*, *there is an instance that happened here*, *one client was waiting for COVID-19 vaccination*. *The client had both*, *a viral load form and a COVID-19 vaccination form*. *He waited for a long time without entering the room for sample collection and what he did was to fold those forms*, *left them on the bench and left the facility*. *For that day*, *it means he missed the service…*.*”* FGD participant, 21 years old male, expert patient, 17 years in care, health center level.

## Discussion

In this study, we found that, patients rated overall PREMs as good or very good with comparable scores, but only a quarter to a third of the participants gave the HSR the highest rating possible. The rating in some of these domains was overall high meaning VL services met participants’ health needs. For instance, participants who reported to follow care providers’ advice were 2-fold more likely to rate VL testing services very good than picking other Likert scale options, meaning VL services they received met their health goals. Findings from other studies showed that HIV patients’ adherence to health services had been associated with sticking to providers’ advice [27, 28]. However, for the patient to follow providers’ advice correctly, there should be a better communication from the providers [29].

In this study, communication has been significantly associated with patient’s satisfaction on VL testing services. Similar findings have been reported in another study conducted to evaluate HIV/AIDS care services in Tanzania where, better communication with providers was associated with accessing health care services [22]. While good communication was also reported in the FGD, this was not the case in lower level facilities such as dispensaries where patients were reported to changing care site due to poor communication. There is evidence in other studies that, poor communication from the care providers leads to poor treatment adherence of which it may affect adherence to VL testing schedules [15]. Communication includes clear explanations of the service provided including VL testing. Good communication with patients is important to ensure sustainable engagement into care, better understanding of instructions and thence adherence to treatment schedules including VL testing.

In the present study, being involved in decision making (autonomy) on VL testing was associated with a 4-fold increased likelihood of being satisfied with VL testing services. Similar findings regarding this indicator have been associated with good adherence to treatment [30], [31]. In FGDs, we noted that, patients had more experience on being subjected to provider-centered instructions than shared decisions. Patients whose care providers make all treatment decisions for them are said to be less likely to adhere to the treatment [30], including adherence to VL testing appointments.

As one of the emerging issues during FGDs, missing appointments have been reported to be a major patient-related barrier to VL testing. Residing far from the clinic and transportation costs were reported to be the main reasons for missing appointments. Cost and distance are widely reported in other published data, where expenses on transport have been one of the challenges contributing to missing the HIV care services [32–35]. It is reported that, in Tanzania, HIV-infected patients may use up to US$ 26.51 per year for commuting to and from the HIV clinic [34]. Some of the patients cannot afford this cost thus prioritizing drug refilling than HIV VL testing visits. In this study, patients who were residing far from their care and treatment clinic reported to be advised for a transfer to a nearby clinic when noted to miss treatment appointments. However, they were concerned with stigma issues in clinics near their residencies for there is a possibility of meeting a familiar person in the clinic. Another reason for missing appointment was conflicting clinic schedules among patients with multiple chronic diseases. Having chronic co-morbidity possess a need to have multiple clinics and is likely to cause competing priority in clinic attendance which may lead to missing the test appointment.

Lack of awareness of VL testing mostly among those with a year or less in care was found to be one of the reported patient-related barriers to VL tests. In this study, survey data indicated that, having attained a secondary school or higher education was significantly associated with accessing the VL testing services. Another study in Tanzania reported illiteracy to be associated with poor utilization of the HIV education on abiding to the health care services [36]. Interestingly, our FGDs results showed that, regardless of the education level, being in care for longer duration resulted into attaining a good knowledge and attitude on VL testing (patient experience). This could be explained by high frequencies of shared information from their care providers which with time, patients may understand and become knowledgeable with the test and other HIV services. But the challenge remains to be lack of conducive environment and sufficient time for health education. System-related barriers on VL testing which were reported during FGDs included shortage of staff resulting to conflicting roles. Shortage of staff have been widely reported to affect the quality of health services in sub-Saharan countries [37–39].

Even though the majority of the patients seemed comfortable with the VL testing turnaround time, we learnt that, there is no clear guidance and timelines for the care providers to share the returned VL results with patients [40]. In the current practice, patients only access their results in the next drugs refilling appointment date. In Tanzania, ARVs prescriptions range from one month to six months intervals, depending on patient’s health status [41] and thus VL test results are available to patients 1–6 months after testing depending on their drug refilling schedules. This practice subjects’ patients with more than 3-months prescription and having high viral load into risk of delaying further investigation and receiving appropriate treatment measures timely.

### Study strengths and limitations

This study has provided evidence that can facilitate development of strategies to improve the uptake of VL testing in Tanzania. It has used combination of methods which has increased the reliability of the data. It however had some limitations. Firstly, we only included NGO-supported public facilities in the city thus we cannot generalize the results to non-supported government-owned, private or rural facilities. Secondly, the respondents were patients who regularly attended their respective HIV clinics. We didn’t manage to receive responses from patients with track record of frequent missing appointments, or lost to follow up. Their experience could have made a different PREMs findings as presented in this study. Thirdly, due to time and limited fund, we included only 6 public health facilities. Including a greater number of facilities in this study could have improved the robustness and minimized any elements of selection bias. Finally, due to limited time and resource, we conducted limited number of FGDs which might have left unearthed barriers to VL testing services.

## Conclusions

While some components of HSR were rated high by one-quarter to one-third of patients, there is a room for VL testing services improvement. The association between VL testing service satisfaction with HSR components also highlighted these important observations. Patient-related barriers to VL testing included missing testing appointment, lack of awareness and over dependency on provider’s decision. Patient’s adequate health education, good communication, patient centered guidelines and adequate clinic staffing may help to alleviate the identified gaps to improving the rates and quality of VL testing services in Tanzanian public health facilities.

PREMs are a key in alleviating identified gaps to improve the rates and quality of VL testing services in Tanzania. The findings from this study are critical not only to designing an intervention and solve challenges related to VL testing services but also in all other chronic disease’s health services in the country, something which provides more strength to the impacts of this study for a positive change in our medical service practice than initially expected.

## Supporting information

S1 Data Quantitative Stata format dataset and do file. The authors have uploaded the complete survey dataset and its do file (Stata format) to serve as reference to the reviewers and editors.
(ZIP)

S2 Data FGDs coded excel sheet. This excel file have the FGDs code book and the representatives’ quotes for each theme.
(XLSX)

S3 Data FGDs transcripts (word documents). (ZIP)

S4 Data FGDs patients’ characteristics Stata format dataset and its do file. We imported the excel sheet FGD patients’ characteristics into the Stata software for conducting simple descriptive analysis. Therefore, a saved dataset and its do file has been shared with editors and reviewers for their reference.
(ZIP)

S1 Appendix Focused group discussion (FGD) interview guide. (DOCX)

S2 Appendix Patient reported experience measures (PREMS) survey tool. (DOCX)
