# Supplementary material for: Patient reported experience measures on HIV viral load testing at public health facilities in Dar es Salaam, Tanzania: A convergent mixed method study
Source: PLOS Glob Public Health. 2023 Apr 7;3(4):e0001024. doi: 10.1371/journal.pgph.0001024 (PMC10081772; doi:10.1371/journal.pgph.0001024)
Supplement: S3 Data — (ZIP) [file pgph.0001024.s003.zip › S3_Data_FGDs transcripts (word documents)/Amana Hospital FGD.docx]

Patients Focused Group Discussion (FGD) at Amana Hospital

**INTERVIEW DATE: 28- December- 2021**

**DEMOGRAPHIC CHARACTERISTICS**

| **ID** | **Age** | **Sex** | **Marital Status** | **Education Level** | **Source of Income** | **Duration (Year in Care)** | **Residence** |
| --- | --- | --- | --- | --- | --- | --- | --- |
| **1** | **29** | **F** | **Married** | **Form Four** | **Expert** | **4year** | **Buguruni** |
| **2** | **58** | **M** | **Married** | **STD Seven** | **Volunteer** | **17year** | **Kitunda** |
| **3** | **39** | **F** | **Married** | **STD Seven** | **Business** | **10year** | **Pugu** |
| **4** | **57** | **F** | **Married** | **No Shool** | **House wife** | **7year** | **Mkuranga** |
| **5** | **65** | **M** | **Widow** | **Form four** | **Retired** | **12year** | **Magomeni** |
| **6** | **63** | **F** | **Widower** | **STD Four** | **Business** | **20year** | **Ilala** |
| **7** | **46** | **F** | **Single** | **Form Four** | **Business** | **10year** | **Temeke** |
| **8** | **53** | **M** | **Married** | **STD Seven** | **Expert** | **6year** | **Mbagala** |
| **9** | **28** | **M** | **Married** | **Form Four** | **Expert** | **11yaer** | **Tabata Kimanga** |

I: Hello, how are you?

R: Very well!

I: Welcome, today is the 28^th^ of December 2021 and I am at Amana hospital doing a discussion with CTC clients at this hospital. Welcome

R: All, Thank you very much.

I: like I said in the beginning the aim of calling you here is to get your views and experience in HIV viral load testing which is one of the services provided at this facility. We would like to find out why people do not do the test or what are the challenges that you face when doing the HIV viral load test okay? So there are questions that I will ask to guide through the discussion and these questions focuses on that side of HIV viral load testing. I know that most of you are experienced like you told me, there are some of you with different number of years in the service so we expect to get lot of answers, right?.

R: Mmm (yes)

I: your participation is voluntary so I would also like us to make use of this voice recorder that will help us to store our information. So, feel free because this is a discussion and I would like that each one of you actively participates in giving his/her views. So, in these questions that I will ask, they are just ordinary questions, no wrong answers in whatever will respond, okay?

R: Okay.

I: so, feel free, okay. First of all, I know that all of you have experience in getting this service at this center, okay, now please tell me what are the services that you are supposed to get at this center when you come for clinic. What are the services you are actually supposed to get? You are now welcome for discussion. Who would like to start our discussion for us? Welcome participant number 2.

R: Number 2, what I understand is that when someone comes in the morning, he is supposed to start at the reception, and there is a box that has been prepared to put his clinic identity card when he arrives, and then he is instructed to remember his card number which is his file number. This is because they are now saying that it is not good to use names, they use your file number. That means when you put the card he tells you to seat there, you measure your weight, pressure. So when the cards are given they use them to find your file which were already prepared they are brought to the table. When they have brought them to the table, they call out numbers, the number they told the client to memorize, his file number. So after that they will start to arrange the clients, because you can find there are only 2 counsellors, so they say from this number to this number you will go to room number 9. The rest from number… to this you will go to room 12. So when a client goes It depends, one can have high viral load, there is a list and he has to talk to his counsellor, … they continue to counsel him and every time he comes there is a place to complete in a file, until that counseling season ends. There is another one who does not have, maybe he need to take the test, and the test could…. He has taken 6months and the results have not returned maybe it will come next month. That means, they will give you medication for 6 months maybe and schedules a date for testing and when you come, they will take the test before the last date for testing. So, it depends on him/her appointments.

I: Yes

R: Number 2, some of the patient may miss the test because they do not know if it’s their testing date. He might attend, but soon after getting drug, he walks away. The service provider will keep on calling the file number, but doesn’t show-up. Because he had already taken medication, so he will come maybe the next dates, that is when he will come and take the viral load test.

I: okay, thank you very much.

R: Mm

I: okay, thank you. We have heard participant number 2, he has started giving us the hints on the services offered here. He has already said number one is measuring weight, that’s right ee, another is viral load testing, okay. Now let’s continue with another participant, what are the services you are supposed to get, he has said measuring weight, viral load test, client follow up, welcome, welcome number 1.

R: Number 1, basically when you arrive at this clinic, firstly the service we get here… when I arrive first I place my card in the box there, I go and get my weight and pressure measurement, when they have found my file I will be directed to go to one of the counselors. You can find that there are 3 counsellors you will be directed in one of the rooms to go in. Once you are done with the counsellor you are supposed to go to the doctor. When you are going to the doctor the counsellor will tell you that if you did some tests before or if you have a test on that day to go for a test. For the results, they will tell you your results they are so and so. So if you have done well and in the beginning you were not doing well, the counsellor will tell you today we will give you medication for 3 months. So, when you go to see the doctor you know your results already, that I am doing good I am leaving with something different. Also, there are other services we are given, like medication for preventing TB.

I: Yes

R: Number 1, At the counsellors they tell you that now you need to take this kit because you are supposed to start taking medication for preventing TB. So when someone leaves the counsellor’s office he knows that there is something I am going to be initiated by the doctor’s. so when you arrive at the doctor’s, the doctor also tells the client, prescribes it for him, also if I have my own personal things I can share them with the doctor because the doctor is supposed to listen to me, so that he can serve me well. So he has to listen to me even on matters that disturb me personally, apart from family matters. So the doctor will also counsel you. When I leave the doctor my file will be, if I have a test I will go for the test, if I do not have a test I will go for medication. When I get to the medication area I will be given the medication prescribed for me, I will also be given my next appointment. Once I am done with that I take my card and medication and leave.

I: okay, thank you very much participant number 1. He has also shared with us about the services he gets. Looking at what he has said, he has hinted on testing, viral load testing, weight measurement, okay ee. He has also mentioned getting his medication, those are all services that he gets. I welcome another participant so that we can finish on this question. Welcome number 7

R: Number 7, I donot have anything extra because it has already been said, when you arrive and are told to go to the counsellor but what I like most is when you come for ARV services there are other services that you get. For example there is a test they wanted us to take, sugar test, cervical cancer, for women, medication for preventing TB, so we are doing well and we thank God.

I: okay, thank you participant number 7, she has also given us her views, she has said that apart from the services she gets from CTC but there are also other services that she has said you also get.

R: Yes

I: Welcome participant number 9.

R: Number 9, we are thankful for the improvements made on the services for clients… before they did not do all these services……

I: okay, okay, thank you very much. Thank you for your very good answers, lets continue. okay, you have mentioned the viral load test like you explained in the beginning, now let’s base on the viral load test. Now when someone says ‘viral load’ what does it mean? Or what comes to mind when you hear the term ‘viral load’? Welcome number 9.

R: Number 9, viral load is concerned with someone who does not use medication correctly, so those viruses grow stronger, reproduce and increase in number in your body, that is viral load. So CD4 decreases and the viruses increase that is the meaning of viral load.

I: Okay, number 9 says that a person who does not use medication has many viruses and that is viral load. Okay, who else would like to contribute something. Number 1 welcome, what do we mean by viral load what does it involve?

R: Number 1, When someone says viral load, it means that there is a test that we are supposed to do in order to know the number of viruses, because sometimes we stay a whole year without accessing the test. So, when you arrive at the counselor’s office, they will tell you to have the viral load test. So, it was in the beginning when I asked the counsellor what viral load was, she told me what it was, that is what I know, that is what they tell us.

I: Okay, okay, thank you very much participant number 1, participant number 2 welcome, do you want to contribute?

R: Number 2, I wanted to contribute that the meaning of viral load is the proper use of medication, that is what is involved in viral load. Because if you do not take medication properly then you can be taking medication but not at the required time, then the viruses will increase. Now you are asking yourself why am I taking medication and the viruses are increasing,

I: okay, okay, thank you very much. Ee I welcome another participant, let’s hear number 3 again.

R: Number 3, I am thankful because the ones who preceded me have spoken because, when they plan for you,…. when you are going to take your medication…. because when the doctor tells you, “You have to set a time for yourself when to take this medication” you need to plan for yourself, you commit yourself, that, …like, I will start taking this medication at 9pm.

I: okay, okay, thank you participant number 3, number 2 and number 1 for your contribution. Is there anyone else who would like to contribute before we finish this part? Welcome number 4, number 6, eee welcome madam.

R: Number 6, Everyone else has already preceded in talking about the services we are given as usual, eehe, taking the medication on time, come for the test, if they tell you to come on a certain date like today they have collected my sample for the test…….you need to come, so that is how it goes.

I: okay, okay, thank you very much, and maybe how often is this viral load test supposed to be done? How often are you supposed to take the test? Number 6.

R: Number 6, I took the test last year in November, and I have taken the test again today (December), yes.

I: okay, okay, so you say the test is done once a year?

R: Yes

I: Thank you very much, you have heard participant number 6. She says it is taken once a year. Welcome number 5.

R: Number 5, Yes

I: How often is this viral load test done? Or how often are you supposed to take the viral load test?

R: Number 5, Once a year, ee yes since I took the test the first time it is today that I got my results. Most of times I take the test in August.

I: okay, okay, thank you very much, welcome number 1 to share with us how often you are supposed to take the test, is that okay?

R: Number 1, Mmm (yes), once a year!

I: okay, welcome participant number 4, let’s hear you, how often are you supposed to take the viral load test? Or how often is it taken?

R: Number 4, once a year.

I: Once a year, okay, thank you very much. Is there any one else who would like to contribute? Welcome number 8

R: Number 8, yes the time for viral load for those on the list (mkeka) we say that they take…

I: what do we mean by “mkeka”, the list of clients or the one we use for seating down?

R: it is the, there are clients who have a big number of viruses, those do not take the test only once a year but they take it twice a year.

I: okay okay, thank you very much. So we can see that the clients who have a high viral load take the test twice, like you said. Is there anyone who would like to add anything? Okay thank you very much. So if this test is to be done for you who is usually the first to ask for the test?

R: Mm(yes)

I; maybe you come to the clinic, who is usually the first to talk about doing the test? Welcome number 5.

R: Number 5, Regrading the service, most of the times, the clinician decides, but some of them forget, others do not take the test (overlook), or maybe some of the clients afraid of the tests, but I’m thankful that when I meet the counsellors, the first thing they just open your file, they review your previous tests, and they advises you that today we ought to do this or that.

I: okay, okay, thank you very much. You have heard what participant number 5 had said, ehee, welcome number 6.

R: Number 6, I, personally when I get to the counsellor many times, I like to remind them because they are also human, they have a lot of things, when it is my date for testing, I remind them that I do like to take the viral load test, so they write it for me or plan something. Like today I have taken the test so I am waiting for the results.

I: thank you very much participant number 7. She has said she helps the counsellor if she forgets she reminds her. Who else, participant number 8, welcome.

R: Number 8, what I can say is that, the viral load test is your responsibility first you need to know its importance. So as you see that I take the test in August every year, when you arrive at the counselor’s you are the one to tell her that today I have a test. So she checks and says yes you do have a test, she tells you to go to the doctor, he writes it for you then you take the test. So first it is important that it comes from the client herself. She should know that he/she have to take the test every year.

I: thank you very much for your wonderful views. Now what is the main reason(s) that this test is being done, the viral load test why do we continue taking the test from time to time? Welcome number 1.

R: Number 1, the reason why we take the test from time to time is that, we are taking medication, how will they know if the medication is working. They need to know through that test, that is why every year they take our sample so as to know if the person is improving or is the same or otherwise. Because someone else is given 6months drugs refill, it’s a long time, you can find that the viruses have increased…

I: They have increased!.

I: thank you very much participant number 1. Welcome number 7.

R: Number 7, I would like to add on how we take the medication. We take the medication every day, that is every day, like for me now when it enters my body there are other things that work with the medication, for example the kidney’s work is to remove the waste, so the tests that we take often help us… how much have our inner things been affected by the medication we take.

I: okay, okay, thank you very much, number 5 would you like to add on the reason why we do the viral load test?

R: Number 5, the reason for testing, like my fellows have said, is to just know how you take the medication, if you adhere to it, ee that is the main thing there.

I; Thank you very much.

R: how do they work in your body.

I; okay thank you very much. Number 8

R: Number 8, to enable the doctors to know my progress, because they cannot know without that test.

I; okay, you have heard number 8, she has said that this is very important to her. Number 5 you have something to add?

R: Number 5, the importance exists and the importance Is wanting to know how the medication has helped you. Because there are times you can be given medication, especially in the beginning, and they…. When you come and test again you find that they do not work properly, so sometimes they change your medication after getting the results of the test.

I: okay, okay, thank you, eehee number 3 welcome. Is there any important in doing the viral load test?

R: Number 3, Yes there is an importance in doing the test, because someone can be given the medication and use it, when you use them you do not know if the medication is working or not.

I: okay, thank you very much, now let’s continue, I know that some of you have done the HIV viral load test, okay. Now I would like to know about your communication with the service providers when you come to get the test. Who can tell us? About how you communicate, welcome number 7.

R: Number 7, like we talked, when you enter the counsellor’s room or the doctor, will tell you that today you have a test, but as a human they can forgot to order the test, and if you know, you will have to tell them.

I: okay, okay.

R: that is your communication.

I: okay, thank you very much. So, when you are talking with your provider, were you given any explanation? I mean did you understand what the viral load test is?

R: Number 7, yes we get the explanation, we know the importance. That is why on the date of the test you remind them, asking to be tested. It means you remember the explanation and you know how important is to test.

I: Okay, okay, thank you very much. I welcome another participant to contribute, number 2 welcome

R: Number 2, Sometimes they use phone calls, if it happens you didn’t show up. That is why now they use phone calls to make follow-up, they call you and tell you, you have a test. So, the file stays there waiting for you, and it depends on the situation.

I; thank you very much, anyone else who would like to contribute, number 5 would you like to contribute something on communication when coming to take your last viral load test, how was it, how did they communicate until you got the test?

R: Number 5, Personally, the last time I came for the test I got it and was informed about the next appointment. The other appointments are for drugs refilling, but now I know, in every August, I have to test. I usually remember and I come on a date that is not for taking medication, it is just for the test.

I: okay, okay, thank you very much. now when it comes to getting the viral load test, I would like to know the language used between you and the service providers, how are you served, do they mistreat you or things like that, I would like to know from you? Welcome number 7.

R: Number 7, generally the services here are very good, they have improved. But in the beginning, it was really discouraging, because there were many patients and few providers, it was even above 100 up to 700 a day. So even the service providers were getting exhausted, sometimes you can think that they are doing it on purpose but they are human beings, they get tired. But as of now the services are very good.

I: Thank you very much. participant number 7 has said, in the past there were challenges but now everything is going well, the services are good. Are you served with respect?

R: Number 7, there is no problem.

I: without being maltreated

R: Number 7, without being maltreated

I: okay, thank you. Anyone else to contribute, welcome number 6, in the area of service, do they serve you with respect? during the viral load test, and how do you communicate with the service providers?

R: Number 6, we communicate well.

I: and the language they use?

R: Number 6, the language is good, they speak well, we understand each other, you get the medication and off you go home.

I: okay, okay, thank you, welcome number 2.

R: Number 2, the issue of maltreatment, there are times when the service provider has problems for example, sometimes they over react, harshly, once they call your card number and maybe you didn’t respond back.

I: okay, okay so…

R: Number2, Yes, even the service provider is a human with his weaknesses so, it happens to quarrel with patients...

I: okay, thank you very much participant number 2. Number 3 anything to tell us?

R: Number 3, I am thankful, the services are good, we are treated with humility, not like in the past. You know in the past we used to discriminate each other, but now there is no discrimination. When you get here the doctor will receive you well, the service providers will receive you well, and provide you with the service. If you see the service provider is busy, ask for a service, you cannot just seat and wait, no, because the service providers also have a lot of things.

I: they have a lot of things, okay, okay. Did they listen to you attentively, welcome number 2?

R: Number 2, I think that exists because sometimes a client in a que can ask how come the other client has spent lot of time in the doctor’s office… let say, more than 10minutes. Staying longer means we are free to explain issues to the doctor so that we can get the solution.

I; okay, thank you very much. Number 2 says that the doctors listen attentively to you and they try and solve the issues with you. I welcome another participant.

R: Number 5, Yes, I have seen that today, and even in past days. So, it’s true that they listen to us and we are counselled very well even if you are found with high viral load, they will tell you what you should do.

I: You get counsel, okay, and were you given sufficient answers to the question you asked?

R: Number 5, Yes, many times we are given answers that are very sufficient.

I: okay, okay, when you are with the doctor are you given a chance to ask him questions, for example you have arrived at the doctor’s office? Number 5, welcome

R: Number 5, we are given a chance all the time, we are given a chance to explain ourselves too.

I: okay, okay, number 3, what do you say, when you are in the doctor’s office, are you given… are you given a chance to speak and be listened to? number 4, sorry.

R: Number 4, you are given a chance and they listen as usual.

I; okay, are you given sufficient answers?

R: Number 4, the right ones! yes

I: okay, number 3 welcome

R: Number 3, they give us sufficient answers…..

I: Okay, thank you very much. Now what would you like the service or providers to improve? Welcome.

R: Number 7, I say the challenge is with people who are not educated because some of them they do not follow what they are told to do. Now for example, if the doctor told you to do the test and you don’t go because you are afraid of the syringe, now who are you despising? Its despising yourself because you are the one with the problem. In the past it was that you take the test but you don’t get the results, they take your sample and when you come for the results, they look for them and don’t find them. And they repeat taking your sample again. Now that was making people afraid.

I: okay okay, that’s in the past

R: Number 7, yes, they would say, “Every day you take two tubes of my blood, my blood is reduced in my body and when I come, I don’t get my results.” Now that problem no longer existing, when we come for the test date, then the results will be available when you come next.

I: You get your results, thank you very much. Anyone else?

R: Number 6, if you do not want to the test then you are not sick but if you have problems you have to take the test.

I; Yes, so where is the challenge?

R: number 6, there is no challenge, I have not seen a challenge, yes, because when I am told to take the test I do so as usual.

I; okay, okay, number 6 says she has no challenge. Number 5, welcome.

R: Number 5, Yes like my sister has preceded me in saying, challenges were there in the past, even I had faced them, those of giving my sample twice.

I: it happened to you?

R: Number 5, it happened to me, every time you come for your results they are not found, and we are not told the reason, they come and pierce you again. But I would do my best that when I come I ask them to take other tests.

I; okay, okay, so it also happened to number 5.

R: Number 5, Yes it happened to me but like 3 years back. But for now I have not faced that challenge.

I; okay, okay, thank you very much. welcome participant number 2.

R: Number 2. The problem is not… those are challenges and we are in a challenge, we already went through those thing. So we were just asking for one thing, If it is possible, to add one person in the lab so that there are 2 because many times there is only one person in the lab (phlebotomy room).

I: one?

R: Number 2, So he should label those bottles then come and take the samples and it could make someone give up because everyone has plans and he is waiting to be called for sample collection. so, if it is possible, they should add one more person in the lab. In the beginning there were two, it used to help a little because one would write and the other take the sample.

I; okay okay, thank you very much participant number 2, she has gone straight to the next question, she has given her suggestion on what should be done. Although she has also mentioned the challenge that there is a challenge in the service providers who take your sample. Number 1 is there a challenge you have faced in the area of viral load testing, why is it that clients do not want to take the test?

R; Number 1, Like my fellows have said, it is not that people do not want to take the viral load test, we are also human, we also have our personal challenges at home. When your time comes for you to take the test you get an emergency, a very important one, this is important but that is also important. So on the day a client is supposed to come for the test he is needed somewhere else. You can find that she is a mother and her family do not know what is going on, so she goes to care for her family and here she looks like she has become a problem. So the challenge is there, sometimes things are complicated.

I: Family, okay, thank you very much participant number 1. She has said there are family challenges that you may face. Number 2, is there any challenge that you have faced in HIV viral load testing?

R: Number 3, the challenge was there in the past, like my fellows have said, you can take the test, I have tested 3 times and I was told they could not find my results. I even cried, and I said “Why is it that every time I take the test I do not get my results.”

I: they can not be found, okay, okay.

R: Number 3, They cant be found, how come, you see. There are others who would be told to go and take the test and they are told “is there no possibility to give me medication, why should I do the test.” But it is important to test so as to know your progress.

I: okay, okay, thank you very much participant number 3, Number 7 welcome so that we can finish up this part.

R: Number 7, I would like to add like the brother there said, in the issue of adding a service provider. You can find that someone is working for an Indian or Arab, he has asked for a short time so that he can come and get the service and go back t his work, both places are important. Now if he loses his job his life will also be destroyed because we are delayed here, you can seat there and waste 30minutes, a mother Is alone …. So the major challenge is to add a person it will help, so that If we need the test we can take it fast and go back to work.

I: Go back to work early

R: Number 7, Yes, because you know how our jobs are.

I: okay, thank you very much. Now let’s move to . you would like to add something number 9.

R: Number 9, NO

I: Okay, okay, thank you. Now let’s look at what you expect your service providers to improve in the things you have spoken about, number 2 has already contributed that service providers should be added in the area of viral load testing in the lab, okay, ee, so what else should be done or you expect to be done. Welcome number 9.

R: Number 9, My contribution is that those areas they take the test should improve the testing equipment. For example, when they are trying to take the sample a person can not see properly until he uses spectacles, they are too small. So he uses a lot of time to write, I am asking that they wash those things febrile so that he can write properly and use less time.

I: okay thank you very much number 9, ehee is there anyone else who has something to contribute Number 2 do you have something to add?

R: Number 2, let’s look at a challenge in the very beginning when someone comes and places their card there, there is something that was going well in the beginning, that when you arrive you know your file number, but there were number that were going as number 1,2, so as to go orderly according to who arrived first. So you can find someone who came in the morning but his file is not yet found but he has number 1 or 2 so we ask how come he has not gone in yet to the counsellor while he is here. So that we do not fuss the one who came first he gave his card first should be taken to the table, there he is given a number, but the one whose file has not been found… the procedure of going by who came first is a good one it should continue.

I: okay, thank you very much participant number 2 has said we should improve on how to serve the clients, clients should be served according to how they came in. is there someone to add to that. Number 5 would you like to add anything?

R: Number 5, what I see is the same as my fellows have already said.

I: Yes, okay, thank you very much. now is there any ease or difficulty to get the viral load test. Number 5 welcome

R: Number 5, When it comes to taking the test I have not seen any difficulty. The difficulty comes in the delay of being served because of one person.

I: Number 7 is there any difficulty?

R: Number 7, Difficulty

I: or is there any ease?

R: Number 7, I haven’t seen any difficulty but , I don’t know if now they have special numbers, when a certain number reaches then no one else can go for the test. Because when I am there the doctor tells me if you want a syringe you have to go there to the test room, and another lady shows up and say has my number reached, it seems…

I: There are some numbers…

R: Number 7, Yes, when it reaches a certain time there are some files maybe at the end, but all of that is because he is alone. In the past we used to come at any time and all of us get the test.

I; Thank you, so it is easy for you,

R: Number 7, It is easy for me.

I: The difficulty is only here

R: Number 7, except for how they work, the madam needs to be given assistance…

I: Thank you very much, maybe participant number 6 is there any difficulty you have faced or is there any ease In getting the test?

R: Number 6, I have not faced any difficulty.

I: you have not seen any difficulty

R: yes

I: so it is easy, okay, number 7, I mean number 8

R: Number 8, Yes, on the side of difficulty it is like it has been said, for example that woman is alone, she becomes slow, but if she gets someone to hold her kids then I think things will go faster.

I: okay, thank you very much number 9

R: Number 9, it is the same

I: okay, okay, thank you very much. now let’s continue, in the side of challenges it is ok, but on the side of costs, is there any challenge in side of costs that you face, maybe in getting that test?

R: There is no cost

I; Number 5 welcome

R: Number 5, I have not faced any costs and I don’t think there is a cost.

I: okay, okay, number 4 what do you say about the cost that you have faced?

R: Number 4, I have not faced any cost

I: mm okay, number 3

R: Number 3, There is no cost.

I: there is no cost in testing

R: Yes

I: okay okay, number 4, number 2 sorry

R: Number 2, what I see is that I have never heard that there is a cost in testing while it your right for them to treat you and it is your right to be served by them and she is there for you. So where is the cost written that there is a cost, there isn’t any. That someone was told to bring money, no.

I; okay okay, thank you. Number what do you say?

R: Number 1, There is no cost.

I; There is no cost

R: mm (yes)

I; and what of this distance challenge, what do you say about that, I know everyone is coming from a different place?

R: Number 2, That is outside their scope, that cost of distance. The issue of distance really depends on the client because you can be told from the place where you live there is a center there, and you say I can not go to that center, that is why there are options so that someone can choose, choose where he wants to get the service. If you take him there the result is that he says I will not go, that is how we get another new, it came from another center. So it seemed that if you transfer someone by force without his consent he wont attend the clinic. There are people who come from Kibaha, others from Morogoro, others from Mafinga, they come here and get the service as long as they follow procedures. But if you say it is far you should transfer, that because you live far we are transferring you there, he says “ okay transfer me but I will not go, you will take those medications yourself” so it becomes a complaint, why should I not listen to the one who takes the medication, did he complain that he uses too much money for transport?

I: No

R: Number 2, he has come at the required time, yes, so the issue of distance is not an issue.

I; okay, okay

R: Eee (Yes)

I: Thank you very much participant number 2. Welcome number 7

R: Number 7, The transport cost might be there to some of us. I once faced the challenge, of course they advise us to register at a nearby facility and sometimes they force us to be transferred to a nearby facilities. But we fear the stigma. The good example is me, I once lived just near Kiwalani facility, the CTC is at the open space, if I go there, everyone knows me, now can I really take medication there?

I: is it difficult?

R: Number 7, from my view it is something that is impossible, because when you enter there, the first section is open, everyone knows where you have gone, what you have gone to do, that is why we want Amana. It is not that we don’t have nearby facilities we are afraid to be stigmatized, people would start pointing of fingers, it is not good.

I: okay, okay, number 6?

R: Number 6, it is like that

I: Ee?

R; Number 6, just like that

I: About distance?

R; mm (yes)

I: okay, okay, let’s go back to the service providers, we have already talked about that challenge that you face. What about the date of the test being different from the date of taking the medications mmh, the date for the test is different from that of taking medication, welcome number 5.

R; Number 5, It is usually like that for me, it does not usually bother me, when I come he writes for you, many times the doctor will really ask you, that please make sure you come on your scheduled date for testing.. it does not bother me.

I: it does not bother you.

R: number 5, Ee, for me viral load testing is in August and my date for taking medication is in June.

I: Yes, okay. Number 1 welcome

R: Number 1, Firstly the protocol they have put things good, because the day I come for my test they look for my file, I give my sample then I leave! When I come on my clinic date I will not need to go through seating and waiting and the whole process, no. I go to the counsellor, I get my results, I go to the doctor and he writes my prescription for me I go to the pharmacy I take the medication and I leave. So we are grateful that the date for the test and the medication is different, it is good and it makes things easier.

I: okay, okay, thank you very much. welcome number 2

R; Number 2, that is many times they had given education on data, the data is that if like me I take my test in August if I take it in June then in the data it wont show that I took the test and in August again it will show that I need to take a test. So that is where the challenge started so that the date he is scheduled for the test he has to come on that same date because of his data. He can take it before but when the date in the system reaches they will call him and tell him you are supposed to take a test, but he had already taken the test, it wasn’t changed in the data. So I said he should use if it is your date for testing the same month you should do the test so tthat you are given medication for 6 moths, so that you come for that same date so that data is not disturbed.

I: They are not disturbed.

R; Number 2, Now I think that everyone was told such and the reason for coming today in June and you give your sample in August. They say why you don’t give your sample in June while I am here, they tell you that is when your date that was schedule is and the months that were counted amount to August. So he also has to explain to you, he instructs you how to put it, that it might be a disturbance, eee (yes)

I: thank you very much number 2 for good views, number 7 would like to finish up, welcome

R: Number 7, That has touched me, because my date for the test was 11^th^ November, but when it reached September I got a funeral, my aunty who raised me passed away, I went home to Tanga, but I called to tell them that I am travelling I have a funeral. My date for the test is 2^nd^ November, they told me you can go, when you come to take your medication you can also take the test. Now I don’t know if the data will be changed or I will have to take the test again and I have already taken it.

R: But you had given them prior information

R; Ee

I; okay, okay.

R; I took the test and I came back the results were not there because of data. They were not there.

I; okay, okay, like participant number 2 said I think that like you gave the information they have your record, okay ee

R: Mm

I: thank you, now let’s move from the issue of dates to the issue of the lab failing to do tests, what do you say, is there such a challenge?

R: Failing to do which test?

I: That is when you take samples and they fail to test them or give results

R: that challenge exitsts

I: Number 1

R: Number 1, mainly we get it from our new clients, when you take them… the challenge has started now but in the past we used to get good cooperation, now days when you take samples of a new client you have to test for clav if CD4 is low. Now for that client they don’t test it on the same day because if they make a list and the results have come in today, they don’t test for that day so you can find that a client has already left, he Is at home and only thinking about the date of returning and he Is new. And maybe I took enough time to talk to him and he has accepted the service, I come and call him and tell him you have… your results are out. Although we prepare him psychologically that you will come for your results but some of them are hard headed.

I: Hard headed, okay, okay

R; Number 1, the person refuses and says I will come on my date and you will give them to me, I am not in Dar, I have given him medication for 2 weeks and then he says he is not in Dar. So you have to talk to him slowly, that is a very big challenge with new clients. We were told that new clients will be given priority to get results early so that we can know how many CD4 they have so that they can test for clav, some of them come with a very bad condition.

I: okay, okay, thank you very much, is there anyone who would like to contribution the challenges in the lab, number 5 which one have you faced?

R: Number 5, no I have not faced any challenge.

I: okay, okay, Number 3 and you?

R: Number 3, I have not met any challenge

I: Okay, okay, and concerning equipment, are there challenges you face when it comes to the equipment for testing like our fellow number 9 said there are those for writing

R: Number 9, the ones for writing are very small.

I: the ones for writing are very small which causes him to fail to read properly

R: Number 9, the print becomes very small

I: okay, okay, what other challenge do you face in the equipment

R9: when it comes to equipment like these for testing here we have them

I: okay

R9: I don’t think there is anyone who went back because there were no bottles to put blood in, It has never happened, maybe what you said that the print is too small. But on the issue of the space being small its because of one person. Because the person who is there is busy writing in the small spaces …. And then now she comes back to take the client’s blood so you can find that she uses even up to 30minutes for one person, if they are 20 it depends when it will give

I: okay

R: so having 2 people will make it better

R: when there are 2 … even if its not a medical person there should be someone to support her to write

I: Mm

R: On the bottles

I: okay

R; this one takes the sample and bottles leave

I; and the other one writes, thank you.

R: and there is also a book to write viral loads, and there is another to write CD4, there are 2, more than 2

I: okay

R; she must write in all of them

I; okay okay thank you, number 9

R: Number 9, we only ask that they improve when they are removing the fero in looking for a vein, the one that is like a rope, now many people share that, and other people have skin infections. We also ask if they could find us, I don’t know the meaning, I don’t know the finances also if it is enough, because sharing is not good, everyone has their own skin diseases.

I: Thank you

R: And there is only one rope and so many of us share it.

I: okay, okay, thank you, those are things that should be improved. Number 9 has also contributed on things that should be improved. Okay, now let’s continue, I would like to know, what helped those who it was easy for them to get the viral load test, that is what made it easy for you to get the viral load test, I know that all of us have taken the test this year, is there anyone who hasn’t done the test this year?

R: I have

I: Number 9 has, Number 8 has.

R: Number 8, I have

I; Number 7 has,ee, number 6 also has, number 5 has

R: I have

I: number 3, number 4 have you taken the test?

R: Number 4, I am coming back in January

I: January, okay, number 3 what about you?

R: Number 3, I have done it today

I: Okay, thank you, number 2?

R: I have

I: You have, number 1 has, okay thank you very much. now in getting the test, what made it easy… made it easy to get the test?

R: to get it as a client?

I: You as a client to get it ?

R: A client to get the test?

I: Yes to get the test

R; or to get the results?

I: to get the test first, we will talk about the results, the test first. What made it easy for you to get the test?

R: Fast?

I : Yes, number 1

R: Number 1, I got the test fast because I have my schedule when I take the test, so when my month arrived, it was different from my normal clinic date, So when it reached I came and I was told today you have a test. They looked for my file and it was taken to the doctor and I went to que. The person from febrile came and called me, he served me and told me, “you came only for the test eeh, so you can go home” because I was careful about my date and I did not pass it…

I; thank you very much, welcome number 2, you would like to add something

R: number 2, there is where I would like to add something, it is that if the date that I remember is the one then there is no problem, the problem comes for those who have been called 2, 3 times. Your file was taken from data to the overseer, she made the calls then those who agreed to come she put them aside, so now maybe the number was not reachable, and your supporter number was not reachable, so it has to stay aside. Now if it is put aside and the office is not a place for files then they have to take them back to position. So when you come, “my test was to be done in a certain month” then you have to wait for them to look for your file because it went in and out of position because we did not get your information or even from your supporter. So they have to look for your file and because it is your right to do the test. But that whole process will cause you delays.

I: okay okay, thank you very much. Number 5 what made it easy for you to get the test?

R: number 5, I think what made it easy is coming on time, just adhering to the schedule.

I: okay, okay, thank you very much, number 3 would you like to add something?

R: Number 3, Yes, I am also thankful today it has been very easy for me. Today when I arrived I put my card in the box, they took it and looked for my file, they found it very easily. They took it to the vaccination area, I was called there, they asked me a few questions, I answered them and I got vaccinated. I came back there to the table and entered the counsellor’s office, then straight I entered the doctor’s office then they told me to go straight to the testing area. I did not take much time in the testing room, it wasn’t even 20 minutes, not even 15minutes, just like 5 minutes, I went in and came out. I went for my medications, took them and I am done.

I; thank you very much, now let’s go back to this; there is a challenge of waiting for the test, in the beginning we spoke about this, is there anything to add now? The issue of time, that you are scheduled to take the test today and you have come early in the morning and you have waited, is there that type of challenge in the issue of time? Welcome number 2.

R: Number 2, the challenge can be there because you have come in the morning, they look for your file, now it depends how many people’s files have gone to the lab. So, it is not that those who are not scheduled for a test on that date are separate, no, you will have to que after those you have found waiting for those who are waiting for the test... that is where the delay comes from. I think they could separate that those who is not their date should be separate from those whose date it is so they give their samples separately.

I: okay okay, thank you very much for that, is there anyone else who would like to add on the issue of time. Number 8 I can see you smiling, do you want to add something?

R: Number 8, like my fellow has said, that is the issue…., because those who comes for testing and the one who came to take medication are not siting separately.

I: okay, that means they are put in the same place

R; Number 8, so those who came first to take medication, has come before you, you have come now just to give your sample and leave. Now you find that he was the first to arrive and the service provider cannot say that because you have come to take the test, you should be served first, it is not like that.

I: okay okay, thank you very much for your views. Now let’s go back, I know that all of you have taken the test but there is a challenge that we would like to discuss a little. I know that all of us know that we faced the corona pandemic, is that right, now was this one of the reasons that some of you, I know that all you got the test, but just tell me about COVID-19, was that a challenge for people to get viral load test in that time? Welcome number 1.

R; Number 1, That time when the hospital was under the government and they used to send us text messages and call us that you are needed at center so and so to take your medication. There were certain centers that we used to go to get medication. Now there were some clients who were used to when they come to Amana, they can say I lost my card but today is my date, and he does not know his file number, now you can find that the other client who went there and needed those services, like the way he used to get them at his center. And secondly, I am supposed to give… How can we serve you while you do not have your card or any other identifiers, you say, “I will give you my 3 names and you will find me in the data” but the data is not like yours here…? so it becomes a challenge. You go to someone’s center and you explain to him what you need but he can’t help you until you tell them your file number and you tell him that at my center we just mention names and they serve us, help me to get medication, and someone else says I have come for the test, so the answers are different.

I; Different okay, okay

R: Number 1, Yes, because the days were busy

I: okay, and I remember that the hospital was taken so as to… some clients to stay here right?

R: Number 1, Mm (yes)

I: okay, okay, what other challenge, Number 2 welcome

R; Number 2, so when I was listening and you were talking about vaccination, right?

I: Not vaccination, I was talking about…

R: Number 2, That people don’t come because…

I: COVID-19, how does COVID-19 affect you getting the viral load test

R: Aaa viral load

I: Yes, I know all of us have tested but I know we have friends…

R: Number 2, What they did for those who had already tested were… transferring them to other center, like Buguruni in the council office. For those who did not have the test they were told to come at 7, …..from 7.30 there up to 9, to get service there. If come late, then you would have to come to Amana, but that Is for the one who had a test scheduled, I think before they opened this part people could use the TB facility there, so that who had not yet taken the test could take the test. Although it did affect because does not come to the office that we used to….. and this affected us.

I; okay, okay, welcome number 5

R: Number 5, During corona even I faced some challenges because the services for example of getting medication was shifted to Buguruni. However, during my scheduled test date, I came here and did not get any problem.

I: okay, thank you very much. after looking at COVID-19 now we are going towards the end. I would like to know how long did it take after taking the viral load test for you to get your results? Number 9 what do you say, how long did it take for you to get your results?

R; Number 9, it depends on the date you were scheduled to come and take medication at the center. So when you come on that date for your medication you will find that your results are ready.

I: okay, okay, number 8

R: Number 8, it is the same, that is the time, because if I leave in May if they schedule me to come back after 3 months, when I come back on the scheduled date I will find my results.

I: okay, okay, thank you very much, number 7?

R; Number 7, it is the same with me, maybe the date for this of yesterday will change

I: okay, okay, number 6, what do you say, how long did it take since you took the viral load test until you were given your results, did it take 3 years, 2 years or 1 year, or how long did it take?

R: number 6, It did not take long.

I; it did not take long?

R; mm

I: okay, okay, they were given to you on time

R; Yes

I; okay, number 5, what do you say?

R: Number 5, Even for me it did not take long, it was as usual, December when I came to take my medication I was given my results.

I: okay, okay, and that time was okay for you?

R: Number 5, Yes it is just ok, I took the test in August, in December I came to take my medication and I got my results.

I: okay, okay, number 4, how long did it take after taking the test

R: Number 4, it did not take time

I: it did not take time?

R; Mmm

I: how long did it take? Were you given your results in the same time you usually get them?

R: Number 4, if you go there where they give us our results, I got a challenge like one day… like once or twice, that they do not see my results, but it did not take too long, they repeated to take my sample again.

I: So, you took the test again?

R: Number 4, I took the test again, when I came back I got my results.

I: Your results, okay, when you got this challenge that they cannot see your results, what explanation did they give you?

R: Number 4, They took my blood sample again.

I:they took your blood sample again.

R: Mm (yes)

I; they did not tell you why when you asked them why they did not have your results, what did they answer you?

R; Number 4, They said there are no bad results but the results have not come. Mm

I: okay, okay, thank you very much participant number 3 how long did it take after taking the viral load test?

R; Number 3, if you have taken the test today, it is until the next appointment you have for clinic when you come to take your medication that Is when you will find your results at the doctor’s.

I: okay, okay, and the time taken was it normal or ?

R; Number 3, it is just normal.

I: mm why do you think it is normal or you think it is just okay?

R; Number 3, because I don’t know they have felt sorry for us, because you can take the test this week and then maybe they tell you to come back after 2 or 3 weeks, so I think they see it as a disturbance, so why do they just come on their dates when they come and take medication they will get their results.

I: so your results were not late?

R; no they were not late.

I: Thank you very much number 2 how long did it take for you?

R: Number 2, What I am saying is that it is not that you are told to come after 6 months, after maybe3 months your results may be out but how to access must come to clinic. Some of us our HIV condition is unknown by relatives and friends. Now, if the provider sends them and find your phone is with your dad or mom, so they will ask what are these results for again?

I: okay

R; Ee(yes)

I: and you got your results when you came for clinic?

R: Number 2, I got them, after 6 months, had already come a long time ago.

I: and participant number 1, you got your results in your second appointment? Or did you get them by phone, were you called to be told your results?

R; Number 1, they cannot call me, as per protocol and the system, they cannot call you. When you come for your next clinic that is when you will be given your results. As per protocol we do not call

I; Mm okay, okay. Participant number 5 were you called or did you come to get your results during your clinic?

R: Number 5, I came for my clinic as usual, on the scheduled date to come and take medication

I; the date you came to take medication is the date you took your results?

R; Number 5, Ed (yes)

I: and participant number 9 what do you say?

R; Number 9, it is that way, the day you come for clinic on the date I was scheduled after 3 months or 6 months, or 1 months, that’s when I get my results.

I: okay, okay, thank you very much. now how was the feedback when you were given your results? What was done was it easy or difficult in receiving the results, let’s look at that, if there was any ease or difficulty in receiving the results?

R; Number 1, there was no difficulty, it was just easy because the test is in November and the date for coming back is in January. So when I come back I find that everything is ready, all they have to do is read them to me, that’s all. Even if they don’t read them to me I must ask them, I took the test I need my results. So the counsellor will tell me.

I: okay, how was the language used to give you your answers and how was your communication with the service provider?

R: Number 1, Aa because my results every time I test are zero, so he told me congratulations, continue to do what you are doing because your results are very good, continue to do as you are doing.

I: Thank you participant number 2, how easy was it for you?

R: Number 2, The results are usually in the file, yes. So maybe for the one who receives the results that are not zero… you start asking where is this coming from, you are surprised and start to panic. But they tell you not to panic, I am not the one who tested them, so that is where a little bit of panicking come. But If they know how my results are and they show zero then they say continue to be that way, take your medication on time. So maybe the one who does not have a good result that is the one they will have to say wait, let’s talk, why are your results like this. Sometimes they don’t have time to take the medication.

I; Yes

R: Number 2, so you have to find a way that you will be able to take your medication. Yes, plan it well with your counsellor, so that you do not go back there.

I: Mm, okay and the language used to give you your results, how was it?

R; Number 2, the language used is a polite one, they say things like these are your little results, no. they would say something like your results are good, continue to take your medication on time. But you your results are not good, we will have to seat and talk, where are you stuck, is it in taking the medication on time, that you fail to take it in the time planned. You will have to look for a way to take it on time.

I: Thank you very much. Number 3 what do you say is there any ease in getting your results and what kind of language do they use?

R: Number 3, There Is ease and the language is good.

I: What makes it that way?

R; Number 3, You can arrive at the doctor’s and say “Doctor I have come, today is my date, and I have come to take my results,” the doctor will check for your results. And yes there is a challenge when it comes to the results as my fellows have said. You can be told you have viruses like 3months ago, you can find sometimes you have 200 viruses, you are told, “okay, try and take your medication on time” When you come on the next appointment to test you take the test, when you come back you find that you have 3000 viruses! You must be surprised, “ doctor why is it like this?” you become afraid, you pressure goes up. The doctor will try and help you cool down, “Please listen” he counsels you, although you got counsel before from the counsellor but they doctor will also counsel you. If you do not use the medication on time, or there are othere things, eeh, you know some of us drink , eeh, sometimes when you drink you come and take your medication at 8, you forget, you remember at midnight you take the medication at midnight, it will not work.

I: Okay, okay.

R: Number 3, you see someone else, you know all of us are grown-ups, about sex, unprotected sex. So the doctor will tell you if you are having unprotected sex you should stop.

I; okay, okay, thank you very much participant number 3. Maybe participant number 4 what was it like for you, what made it easy t receive your results?

R; Number 4, Aaah I received my results as usual, it was not hard, yes

I: okay, okay and the language used in getting your results?

R: Number 4, the language was good, not bad.

I; Not bad, okay, number 5 welcome, lets finish up

R: Number 5, Yes, I also received my results well and the language used was good. Although I got a small challenge, because last year they were 21, today I was told they are 22, it means an increase of 1 I don’t know, they told me …

I: there is a place you did not do well

R: Number 5, I didn’t do well, where did this one come from. Even this 21 was not supposed to be there, but we talked

I; Yes, yes

R: All: Yes

I: okay, okay, thank you very much. Now in finishing up, number 7 you can finish, while receiving your results what made it easy?

R: Number 7, What made it easy for me to receive my results is my communication with the doctor. The doctor truly knows the importance of his role, and he is at work, doing his calling. We used to talk very well and he share results and I accept them. And if there is anything extra, any advice he used to advise me. I think it is a good communication between a client and a doctor, that makes it easy to receive results.

I; Okay, okay, thank you very much. now let’s go to the issue of education, we would like to talk about the education you get, but education concerning viral load, okay ee. Can you tell us about the viral load education, how do you get it? How is that education given? Is that education given at your center? This is our last question, welcome number 5.

R: Number 5, we do get education, the first thing in the morning when you get here and leave your card there at the first point of counselling, then they start teaching, reminding us what to do, reminding us about the test, if your results are not yet how to ask the counsellor, we get that education even before we get to the counsellor.

I: okay, okay, thank you number 5 has spoken that you get education in the morning when you come to the clinic here. Okay, number 2 how do you get education? And how is it given?

R: Number 2, Education is available in the morning…. Just like she said in the morning, they teach us on drugs adherence, if you do not take it on time then when it come to your viral load test, the results will tell them that you are not using ARVs properly.

I: okay, okay

R: Ee (yes)

I; and this education that you get, how often is it given?

R; Number 2, per day?

I: Ee(yes)

R; Number 2, in a day, those who arrive in the morning, they will get the education, and those who come at 10am, they will also be taught.

I: Number 1, welcome, what education do you get?

R: Number 1, Yes we get education, we get it when we are taught in the morning, along with what the brother said, to make sure you adhere to the medication, to ask for your test results, to ask for the test if it is your turn, and now they have added education on the vaccine. And the education on testing for cervical cancer for the women, we get those services.

I: okay, okay, thank you. How often is it given?

R; Number 1, like this one of the cervix they give it when they see people have come in In a good number. They give it while we are queuing too so that someone can know that this cervical cancer test concerns 1,2 ,3

I: Thank you very much. participant number 8 welcome on the issue of education, what education are you given when you come to the clinic?

R: Number 8, Aa the education is the same, adherence to medication, that is taking medication on time, ee they remind you to do the viral load test, ee and other things that you ought to do we are taught all of them in the morning, in the afternoon and like my fellow has said.

I; oaky, okay, thank you very much, is there something else to add

R; Number 7, The education we are given is that one on viral load testing, unprotected sex, all of them we get the services. We get the education on time and In a proper way.

I: and how is it given?

R; Number 7, it is given as they said when you come in the morning, you will find everything, when you come again at 10 you will also get, but if you are late you cannot get those things, so if you want them you should come early.

I: okay, okay, thank you very much. Number 3 would you like to add there on the issue of education, what do you say, what education is given?

R; Number 3, like the ones who have spoken have said, adherence to medication, and other education.

I: thank you very much, and what language is used in giving that education?

R; Number 4, Normal language

I: Mm English or Swahili?

R: Number 4, Swahili

R: Number 1, the language used Is Swahili but the way we study is like a teacher and student, they use participatory approach, it is not all the time that a teacher will teach, sometimes he will seat and make the class laugh so that the kids can come back to class, that is the language they use.

I: okay, okay,

R; Mm

I: Thank you very much and in that education that you are given, are you given a chance to ask questions while they are teaching? Or do they give the education from start to finish and leaves? Do they give time to listen to the questions? Number 5 welcome

R: Number 5, Yes we are given the time to ask questions. It is the first thing after he finishes giving his advice, but there are also questions that he gives, and anyone can be chosen to answer the question. So we answer his questions and he gives us a chance to ask any question.

I: okay, okay, and how far does that education help you?

R: Number 5, it has helped us or it has help me a lot because I came from there, I started using the medication when my CD4 was 70 I think, today it is 900+, because of listening to the instructions. And I had I don’t remember how many viruses, back then we were not tested or they do not tell us, but I have gone to below zero.

I: okay okay, thank you very much. Number 2 welcome, tell us, are you given a chance to ask questions?

R; Number 2, You when he teaches you need to allow room, and when you see people asking questions you know that they want to understand or they have understood. And even when he is teaching and see that people are not asking questions you need to stop, you tell them this is time for questions. So everyone asks a question, when they ask questions there some who have good questions to benefit all.

I; okay, okay, and when you ask, are you given answers right then?

R: Number, yes, right then

I: okay, okay, thank you very much. Number 1 welcome

R; Number 1, On questions?

I: Mm are you given a chance to ask questions and are you given answers right then and there?

R: Number 1, Ee(yes) when they are educating you ask questions, even when you go home counsellors and doctors you get a chance to ask questions and you get your answers right then and there.

I; Right then and there

R: mm

I: and that education, how far has it helped you?

R: Number 1, the education has helped me a lot! Because I have become a good counsellor to my fellow youth. After getting the education and training I have become a good help to my fellow youth, so it has helped me and I am still helping to save a lot of youth. Someone comes up to you and asks, “how do you do it until you get zero viral load?” I have a lot, so I sat down with the young person I talk to them, tell him that he can do it, so I save a lot of youth.

I: Thank you very much, you have heard participant number 1, the education has helped him a lot. Number 9 what do you say, welcome

R: Number 9, it helps us know many things that we did not know, we are taught, and they keep teaching us every day.

I: okay, okay, number 7 you want to contribute how the education has helped you?

R; Number 7, Personally when I came to test for the first time, I got results that I was positive (words not heard) it’s impossible that I am sick, all this flesh, they must have lied, I stayed home and didn’t take the medication since 2008. 2012/2013/2011 I started getting rashes and I had to tell my sister, she told me to go to the hospital, that maybe I have allergies, but I remember that I had tested and had not gone to the hospital again, it will be a problem. I came here with 13 CD4 but by the grace of God I came on my own, I did not come with anyone else. I came in got educated and that is what has helped me to be here until today, you 13 CD4 is not a joke, I am doing well now.

I; Thank you very much, number 6 let’s finish up on the issue of education what do you say, are you given a chance to ask questions when you are there, and how has this education helped you?

R; Number 6, it is helping me because I can come, I was sick but the infections I had now I no longer have after using medication.

I: okay, okay, thank you very much. we are almost at the end. Now I would like to know are there banners or posters here at your center that talk about viral load testing, maybe fliers, or things like those. We say these are called environment for learning, that when someone sees the banner he learns, ee, if someone sees the flier, does the environment of your center encourage learning like that? Number 9 welcome

R: Number 9, truly there are no banners/posters for learning but we used to get the education daily at clinic when we come, and the counsellor is here and doctors to train us…… yes.

I: Yes, number 9 says there are no posters but the education is given, okay, number 8 what do you say concerning education?

R: Number 8, I say that at this center they have not put any posters, that someone can come here and read, that this is explaining this and that this, no, but the method used here is that of teaching and counselling, doctors give education and when you go home, they are free you can ask any question and they will answer it .

I: okay okay, thank you number 7, welcome

R; number 7, there are no posters but education is given, it is enough of a poster, we benefit from it.

I: okay, okay, number 6 what do you say about posters and fliers?

R; Number 6, There are no posters.

I: None

R; Mm (yes)

I: Number 5

R; Number 5, for now I can say that there are no posters, I remember in the past they were there, now I don’t know if they wore out or were removed, but I remember they were there, posters to remind us about the tests. When you get to the counsellor ask for your date for testing, they were there in the past years, like 5 years back, but now we do not see them.

I: okay, okay, number 4 what do you say on this matter?

R; Number 4, in the beginning like this man said posters were there but now there are none.

I: None, okay, okay, thank you, lets finish with number 3.

R: Number 3, There are no posters now days, in the past they were there.

I: They were there.

R; Mm(yes)

I: okay thank you like you said number 2 ee, sorry

R: Number 2, I would also like to finish up there on the issue of posters, I think there is a time they came, there are others who came and questioned about posters. They said the issue of posters was an issue of 5S. 5S did not want anything to be posted so they had to be removed. 5S or (words not audible) they need to use another method, so what was done here is that they have a protocol with the industries, the protocol looks for a way to post posters that are of standard of 5S. they give out the education but they said the posters were removed because of 5S, because when they come for inspection and they find the posters it reduces the marks because of the way they were placed. So, they said they will prepare a method to post them by following the requirements.

I: It’s given, okay thank you very much we are heading now to our final question, I want to get your thoughts well, what should be done to improve these services of testing the viral load at your center? I know there are those challenges that you’ve spoken about for instance in the lab, what should be so that things become well at the center? This is our last question as we finish. Welcome number 8.

**R: Number 8:** Ee, the improvements that I see should be to increase the number of services providers in all sections because there are days, we find only one doctor, while there’s many patients, so the work becomes hard, so if the facility get staffed, I think the service will go well.

I: Mm, okay, okay, thank you very much. Anything to improve at district level?

R: District.

I: Ilala District.

R: Ee [yes]

R: We don’t know what we should say.

I: You just speak.

R: [laughter]

I: Number 6?

**R: Number 6:** Like they’ve said that staff should be added.

I: Ee only service providers?

R: Ee [ yes]

I: So, there’s nothing more to be added.

R: Correct.

I: Number five welcome.

**R: Number 5:** I think in summary the main challenge that is here is only about the fewer service providers, ehee…. if they improve that other things will go well, because as of recent when we come to test, we get our results on time.

I: Yes.

R: So, I don’t think there’s a bigger challenge, than the one about service providers.

**R: Number 3:** They should add service providers for us

I: Mm adding service providers.

**R: Number 3:** Service providers should be added.

I: Mm anything else.

R: The work will go well.

I: Ok okay, thank you very much, and you number two

**R: Number 2:** The question of what should be done, we’ve spoken earlier they should add service providers, and then at the district, we had or even at the region we’ve spoken, because if we can talk about the district level, there were these antibiotics meds that they used to bring, then later, they removed them, so we were saying if it is at all possible for them to bring them back. I remember when someone had coughing, amoxicillin was available for free.

I: Ok thank you very much, number seven let us finalize.

**R: Number 7:** Dah! I forgot regional-wise, I was asking, there was a time when you come here and you have fungus problems you get medication for free, but now days, it is a problem, you can find someone only able to cover transport cost, but not those medication which are no longer there. The opportunistic infections meds should also be available like ARVs.

I: Ok, thank you very much, and I thank you, I thank you, we have reached the end of our interview, thank you again for your time that you’ve given me and agreeing to sit with me and being able to give your opinions and like I have said they’ll help us in getting the picture and knowing what should be done, and for the reminder that clients should get the viral load test service as much as possible.

R: Okay thank you.

R: Amen.

R: We thank you as well.

I: Thank you very much.
