# Supplementary material for: Patient reported experience measures on HIV viral load testing at public health facilities in Dar es Salaam, Tanzania: A convergent mixed method study
Source: PLOS Glob Public Health. 2023 Apr 7;3(4):e0001024. doi: 10.1371/journal.pgph.0001024 (PMC10081772; doi:10.1371/journal.pgph.0001024)
Supplement: S3 Data — (ZIP) [file pgph.0001024.s003.zip › S3_Data_FGDs transcripts (word documents)/Magomeni Health center FGD.docx]

**Patients Focused group discussion (FGD) at Magomeni Health Center.**

**INTERVIEW DATE: 21^st^ - December- 2021**

**DEMOGRAPHIC CHARACTERISTICS**

| **ID** | **Age** | **Sex** | **Marital Status** | **Education Level** | **Source of Income** | **Duration (Year in Care)** | **Residence** |
| --- | --- | --- | --- | --- | --- | --- | --- |
| **1** | **30** | **F** | **Single** | **Form 4** | **Small Business** | **7year** | **Kimara** |
| **2** | **21** | **F** | **Single** | **Form 4** | **Small Business** | **3year** | **Buza** |
| **3** | **24** | **F** | **Single** | **Form 4** | **Expert patient/Small Business** | **6year** | **Keko** |
| **4** | **47** | **F** | **Single** | **STD 7** | **Small Business** | **13year** | **Kimara** |
| **5** | **33** | **F** | **Single** | **STD 7** | **Small Business** | **7year** | **Jangwani** |
| **6** | **28** | **F** | **Single** | **Form 3** | **Home** | **7year** | **Manzese** |
| **7** | **21** | **M** | **Single** | **Form 4** | **Expert patient (peer health education)** | **17year** | **Mikocheni** |
| **8** | **44** | **M** | **Single** | **STD 7** | **Small Business** | **1year** | **Magomeni** |
| **9** | **44** | **M** | **Single** | **STD 7** | **Small Business** | **8year** | **Magomeni** |

I: As I have previously introduced, my name is Dr. Peter Karoli, I have asked you here to give us your experience about the HIV viral load test at this CTC. We would like to know how you get it, the challenges you face in getting the test so that at the end of the day we as professionals can advise each other and we can communicate with the ministry so as to improve the service at this center and this test in particular. You are welcome to give your views, I am accompanied by my colleague, his name is Zenais, he is a professional at conducting these discussion, so he will be asking the questions and you are free to give your views. We will be addressing you by your numbers, we will not use your names, I hope that is ok, right? So when you want to say something we will say, “Yes number so and so…” although I see you are seating in a number order, but if your numbers are visible, it will be even better.

R: Asante

2: Ok, thank you so much Doctor. As he said, my name is Zenais Kiwale, I work with the Dr. we are here to have a discussion with you, so please feel free. There are questions we will go throught with you, so as to get your views on the services you receive especially on the side of HIV viral load testing, is that ok? So please feel free and try to speak up so that we can all hear each other well. Ok

R: All together, OK

I: well, to start off, I know that all of you attend this clinic, is that right?

R: all together; Yes

I: First question: Now I would like to know, what kind of services are you supposed to get at this center. I know there are several diffent kinds of services offered at this center, but I would like to know about the kind of services you in particular, get when you come for clinic. Welcome, anyone can answer first. Welcome number 3.

R: Number 3, Thank you. At this Magomeni center, all services are offered here; HIV testing, clinic for women and children, there is an eye clinic, dental clinic, blood pressure and diabetes test, and a clinic where women give birth in our hospital and there is also a clinic for clients living with HIV that is to get their medication at their clinic at Magomeni center.

I: Ok, ok, thank you so much participant number 3. You have heard him, he has told us about the services available here. Anyone else, welcome to add to that.

From Dr Peter, Maybe I would like to suggest we improve by talking about the services offered here at HIV CTC, yes somebody else. Thank you number 3, you have opened the discussion well for us. Welcome number 1.

R: Number 1: Services that are available here at CTC include ARVs drugs refilling, viral load testing, and VIA testing.

I: Ok ok, we have heard number 1 telling us of the services available, maybe if I can hear more, you have mentioned VIA testing.

R: Number 1 yes, VIA, that Is testing for cancer of the cervix.

I: okay, it is the cancer of the cervix. Thanks for the good input participant number 1. Welcome someone else, welcome participant number 7.

R: Number 7, I would like to add on the services available here, in the area of testing there is CD4 test.

I: Ok participant number 7, he has added that there is a test for CD4. Any other participant who has something to add? Welcome number 4.

R: Number 4, there is also weight and height measurement.

I: ok ok participant number 4, he has said there is testing of weight and height, welcome participant number 9, can you speak loudly?

R: Number 9 There is also testing for TB, TB medications and HIV medications.

I: Participant number 9 has mentioned that there is medication for TB being offered here and medications for HIV, thank you so much. Welcome participant number 2 so that we can wind-up

R: Number 2 They also offer vaccination against COVID-19

I: Ok, ok, is it offered here at the CTC?

R: Number 2, it is offered here

I: for those clients who come for clinic here?

R: Number 2, eee

I: thank you so much participant number 2, he has added about vaccination for COVID-19, welcome participant number 7 as we windup

R: Number 7, there is 24hr PrEP medication for protection

I: Maybe we should listen to participant number, eee what do you think, ok, thank you all participants who have shared what you understand, perhaps there is something you would like to add? Doctor Peter ?

Question number 2

If No, thank you so much. Now we would like to move on to another question. Among those services you have mentioned, including HIV viral load testing, what do you understand by HIV viral load testing? Welcome number 3

R: Number 3, Thank you, from my understanding this is a test for the amount of CD4 at this center. I mean looking the progress of HIV clients who receive services at this clinic, and their adherence to medication in order to check the level of their body immunity. Because we know that viral load ought to be below 1000 and not above, and this test is done every year. In the past it was done after every 6months but they have changed it and now it is every year. The aim is to check If this client who receives medication here, uses the medication. So when the client comes and the test is done, the aim is to see if his viral load is above 1000 or if its below 1000. So that is my understanding on viral load testing.

I: Thank you so much participant number 3, we have heard his view about HIV viral load, ok, welcome participant number 4

R: Number 4, in my understanding when we test for HIV viral load, it helps us know that when you have a lot of viruses it means you do not take the medication on time but when the viruses are few the Doctor knows that you are taking the medication on time, so it helps us to take the medication on time. If you are scheduled to take the medication at 9 it means that you are to take the medication at exactly 9, every day you are to take the medication at the same time, and that is when the viruses will decrease.

I: Thank you participant number 4, he has spoken more on the importance of viral load testing. I welcome another participant to tell us what they understand about viral load testing, welcome number 8.

IMERGING THEM

R: Number 8, I am not very experienced but I was told there is a test I am supposed to take and today when I came for the clinic, they took my blood sample

I: okay, when they told you to come for a test what test did they tell you it was?

R: Number 8, No I have not been made aware yet but I was told that when I come today there is a test that will be done.

I: Did you ask the Doctor what test they did by collecting sample from you??

R: Number 8, No

I: Ok, thank you so much, anyone else to add to that, welcome number 1

R: Number 1, let me add something, though everything has been said already

I: not everything has been said, there is something you have forgotten.

R: Number 1, in my understanding, the viral load test is for checking our health progress.

I: for determining your progress health wise

R: Number 1: “Yes, viral load test helps to know the progress of the patient since he/she started using ARVs, what amount of virus does he/she have, and if his/her health is progressing well or not, that test checks these things.

I: okay, ok, we have heard participant number 1 emphasizing on the importance of the test. Welcome someone else, welcome number 7, what do you say?

R: Number 7, I think they have said everything, but in my understanding this viral load test is done to test the progress of patient because It is estimated that in one to three months if the patient adheres properly to the medication or if we take the medication correctly, the viruses will diminish even if they were 1 million they will be below 1000 because of this medication we are using. So the main aim is to see the patient’s progress. Therefore, we can use it as a test that indicates that a client is adhering to medication or not.

I: Thank you so much participant number 7, please let’s listen to another participant then we can wind up this section. Welcome participant number 6

R: Number 6 I’m thankful that since I started coming here for clinic I haven’t seen any fault in the services offered, they serve me well.

I: Ok, we were talking about viral load testing, when you hear that you are coming for viral load testing, or when you hear HIV viral load test what comes into your mind first, or what do you understand from that?

R: Number 6, when the test is written for me and my date is due, I come for the test so that I can know my progress, to know whether the viruses have increased or not and I am very happy to know.

I: Okay, so you feel happy when you are told to take the viral load test to know the amount of viruses you have. Thank you so much participant 6, number 5, welcome.

R: Number 5, I feel the same as my fellows. I have the same views so even if I speak my answers will be the same.

I: I would like to hear from you, because everyone has given their understanding on the matter, what is your understanding?

**R: Number 5, my understanding is that when I am told that in a certain date, I am supposed to do the test, it's the chance to know how my health is progressing. So, when they take the sample, I need to know my viral load. When they take my sample today, then on the next scheduled clinic, I will have to ask them for the results, I want to know the results.**

SECTION A

I: ok thank you so much. So after establishing that everyone knows what viral load is I would like to know now, what is the importance of viral load testing for your health? There are some that you have already mentioned, maybe just in addition to them. Welcome participant number 2

R: Number 2 I think that when you give a blood sample for viral load test it helps you know how you are doing, if your viral load has increased or decreased and according to your results you can meet with the Doctor and he can advise you so that you can progress well.

SECTION B

I: okay so we have seen that if you get a HIV viral load test you can know your health progress and you can get counselling from the Doctors. Now how often is that test supposed to be done? Welcome participant number 3.

R: Number 3, in my understanding it is done once, in the past it was done after every 6months, but now the protocol I know is once every year. But it also depends on whether you have come for your clinic and you are required to take the test, then you will take the test on that day. So what I know is that its done once a year.

I: ok, welcome participant number 7.

R: Number 7, what I understand is that it is according to how the client comes for medication. There are those that take medication after every 6months, 3 months and every month, this means that the client that takes medication every 6months in a year he comes for clinic twice, right, for example in the beginning of the year then he comes back in the middle of the year, so those kind of clients do the test only once a year, he is stable. And those who take the medication every month, it depends on their medication. What I know is that a client can take the test today, when the results come back he is supposed to take medication and once he has taken medication after like three months he takes the test again. That what I know, in order to take care of his health.

I: thank you so much, we have heard participant number 7 and he has given us his views, maybe we go back to another participant, number 4 what do you say?

R: Number 4, From what I know, it is done twice a year.

I: Eee twice a year, maybe which months of the year?

R: Number 4, it depends what month it was, maybe it was March maybe, but it depends on which month you took the test, so you will be taking the test according to when last you took the test. For example If you took the test in March you can’t do the test again in April.

I: thank you so much we have participant number 4, welcome participant number 5 so we can hear you a bit.

R: Number 5, my understanding for this 7 years in the clinic, there are those who are given medication for a week and another is given for one month you see, and the one who is given the medication for a month his health is not stable, so whenever he comes to the clinic the test is taken, that is what I know, and if you are doing well, they schedule you for the test after 3months, this is why even me after 6months is when I take the test. I have taken the test after 3months as well in the past and after 1month. So the way your progress is good that how you take the test. That’s my understanding.

I: ok ok thank you participant number 5 has given us his answers, welcome participant number 9.

R: Number 9, the test is taken according to how the health of the client progresses, for example I have 6 weeks ..

SECTION C

I: so viral load test is taken according to how the client is and how your health progresses, thank you so much, let’s go on. Now, who is always first to talk about needing to perform the viral load test, is it you or someone else? Welcome participant number 2.

R: number 2, the one who gives the information is the Doctor.

I: ok participant number 2 has said it is the doctor who is first to instruct that the test is done. Welcome participant number 7.

R: Number 7, in my view it’s the doctor because most of the clients do not know the importance of the test, that is why it Is hard for the client to ask for the test himself. We do not know for instance, how long it takes to do the test according to the stage the client is at, because every client is tested according to their condition, others test after 6months. When can arrive at the doctor’s and say, “Dr. , how about my date for testing, because it has been a long time since I did the test.” But most clients do not know the importance.

I: ok ok, we have heard participant number 7, he has given his views. Welcome participant number 4

**R: Number 4, in addition to that, because there are times you may take the test but find that you stay 6months or more because Doctors are also human beings, they may forget or they may have a lot on their plate.** “ But also you may remind the doctor that I haven’t taken the test in a long time, please let me take the test.” So when you give that idea the Doctor checks your file and they perform the test.

I: ok ok so it happens sometimes that the Doctor forgets to tell you to take the test.

R: Number 4, yes, doctors are also human beings hahaha

I: hahaha ok ok, so if you do not remind him you will not take the test. Thank you so much participant number 4who has said that the client also ought to remind the doctor. Participant number 1 welcome

R: Number 1, it’s the doctor and the patient himself.

I: okay, so it’s both of them, participant number 1 has said its both of them, the Doctor and the client. Welcome participant number 6, tell us who reminds you.

R: Number 6, the first is the Doctor, he checks date and if it is due he will tell you When you come for clinic there is a test. So the day you come for clinic you take the test in order to check your health.

SECTION D

I: thank you very much for your input in this question. We have seen that some of you have said it’s the Doctor and some that it is the client who can remind the doctor and others have said both the two collaborate, ok. So what is the reason for continuing to test HIV viral load through that viral load test, what is the reason? Welcome number 7.

R: Number 7, I think first it increases one’s hope, because there are some clients who if you tell them that they are doing well, haaaaha, he can eat chicken only! He is overjoyed. And those results motivate and the client can become a peer educator that, “I used my medication properly and the doctor told me this and this, so the results come from the client himself. That the way he started clinic and the way he is now, he is doing well, they have helped him.

I: ok ok, participant number 7 we have heard his views. Welcome someone else, welcome

R: Number 3, thank you, in my understanding, first I can say that it is a reminder to every client who attends clinic here, the test reminds him why he is taking the medication. In my view it is a reminder to the client and the service providers who are reminded o their responsibility, that in all the activities that you do that what is important Is that test for the client that makes him come to the clinic every day to get services. So to me it a reminder to every client.

I: ok, ok

R: Number 3, and it is a very important test, and it can protect the client. Also through this test it first reminds the client to be a good user of medication so that the viral load does not increase but decreases, like I said so that they are lower than 1000 because, if he knows it becomes easy for him to take medication. Because when the viruses are many the client continues to have a high viral load.

I: okay

R: Number 3, when the viruses are too many, now he can be at TND, that is, the one who does the test it helps to remind him that even if he had a viral load below 1000, or there is another client who can have a viral load of below 300 or another client can be even below 0, it helps to remind the client that even if he was scheduled to take the test and after taking the test he is told his result is 0, that is TND, so that test that is taken continues to remind him that in your taking of medication you should continue to adhere to the medication.

I: ok

R: Number 3, because it can lead the one with viral load 0 the viruses to rise again they can grow afresh, even from 0 to above 1000. So it acts as a reminder to us. Yes, you take the test for checking viral load, they can be either below or above 1000, so as I said the client can be at TND and at the end of the day end up taking second line medication.

I: thank you so much participant number 3 we have heard your views, welcome participant number 4,

R: number 4, in my understanding it is the checking of my progress in taking medication. And when I find that my viral load is low I cannot be deceived that today I should have sex with her and tomorrow with the other one, so when the viruses are low I can protect myself.

I: thank you so much participant number 4. He has said that it helps him protect himself and his health. Welcome another participant, to add to that. Welcome number 9

R: Number 9, I don’t have anything

I: welcome participant number 8

R: Number 8, I am grateful that like how we are reminding each other they encourage us and helps us continue taking the medication.

I: Participant number 8 has said it can act as a reminder to follow up on your treatment, so that you can get them on time and to know exactly what the client ought to get from the viral load test, ok.

R: Ok

I: now for those of you who have taken that test before, can you tell us how was your communication with the service provider the last time you came for the test? We would like to look at your relationship with them. Welcome participant number 1.

R: Number1, once you are in the doctor’s office he takes your file and you must ask him, “Dr. I took a test last time, I’d like to know my results, because I took the test more than a month ago, I want to my progress.” So the Doctor will read your results to you and he will tell you if you are progressing well or not. You feel peace and joy if your results are good. And when the doctor sees that your progress is not good he counsels you like taking your medication on time, to not pass up the time for taking your medication, getting enough rest and he will counsel you more on nutrition.

I: Maybe I should refine the question a bit more, when you are with the service provider and he is explaining about the test, do you understand the language used?

R: Number 1, its understandable.

I: what do you mean when you say it’s understandable, and what language is used, and we would like to know if there is any challenge in the part of the language used, because some service providers you may find mix English and Swahili, I don’t know whether when they were explaining if you understood the test? Welcome number 8.

R: Number 8, the language they use is Swahili.

I: Its Swahili.

R: Number 8, and I completely understand our service provider.

I: Participant number 7, do you have something to add to that?

R: Number 7, to add to that, I think the language used is correct according to how they received me last. For me when I talked to the doctor he told me my results are good, the viruses are subdued I should continue to take my medication on time but this is according to how a client receives it, others can go and abandon taking medication.

I: ok ok, so how the client receives it.

R: Number 7, eee yes there are some who receive it differently, but as for me I understood that the viruses have been subdued and if I become negligent in taking medication they can rise up again.

I: Number 5 welcome.

R: Number 5, I also asked the doctor concerning my results and he told me that my results show that my viruses have been subdued. I asked him, what do you mean they are subdued, he answered that my results are good. So I continued to ask him questions, I asked him, “Now I can even get pregnant?” He said, “Now you can get pregnant but you should continue to take your medication on time.” And he also asked me about the food I take. So when he asked me questions I would answer according to the question.

I: Ok ok, you have heard participant number 5, and the importance she was told by the doctor. Welcome participant number 3.

R: Number 3, I can say that what my fellows have said is true, although there are challenges especially concerning this viral load test. In my understanding a big percentage of the clients donot know the meaning of viral load or another does not know why they are doing the viral load test. Because those getting the service are not only adults but children also and there are also youth, you see. Because here at our Magomeni hospital the Doctor can tell you that you are to take the viral load test or the CD4 test but in other hospitals the Doctor may just tell you you are to take a test next month but the client does not know what test that is. Another client just does the test and the next time he comes for clinic in his scheduled date he does not ask for the results of the test he did.

I: ok, ok

R: Number 3, so this is part of the challenge, yes the language used is Swahili, but one just says next month there is a test to be done. “For example our father said in the beginning that he came to the clinic and was told there is a test but test for what he does not know and I do not know if he asked what test is to be taken from him.” Because there are two tests, one for viral load and the other CD4 and in a month you can take one of the tests or both of them**.**

I: ok, ok

R: Number 3, so doctors ought to tell clients that this month you are to take viral load test, ok, or this month we are going to take the CD4 test. There is also another challenge, “when the sample is taken, its either the results return on time or they do not come back on time. So you can find that the client comes back to ask for his results and he is told the test failed. That forces the client to give the sample again, and at other times the client asks, all this blood you are taking from us, are vampires? And also there is a challenge with the results, a client results are back and he is told his viruses are subdued, you know we are human beings.

I: Okay

R: Number 3, Everyone has their own understanding and everyone has their own faith, so at the end of the day this viral load test and the results we are given and those with their faith go to be prayed for. “Here at the hospital the Doctor gives the client his results and tells him that your viruses have been subdued they are not seen, so when the client goes back to his faith like churches and he gives a testimony that he is healed of HIV through that test that he did and the results he received.”

I: ok ok

R: Because when the client receives his results he thinks he is completely cured of HIV, while the truth Is he needs to go back to the system of taking medication. So those are the challenges that evolve from not explaining to the client the meaning of viral load, meaning of CD4 or the meaning of the blood sample that is taken. So the Doctor ought to explain to the client when giving him his results and they should explain those challenges of why the results are late or why he has to give another sample.

I: ok, ok, maybe we can ask the client who was tested today. What did they tell you the test was for? Because a blood sample can be for viral load or CD4.

R: Number 8, I was told that it is for CD4 and viral load.

I: You were told both, ok that is ok number 8, today he was tested and he was told it’s for both tests. Welcome number 5

R: Number 5, what I want to contribute is that those lab personnel should be very careful because the challenge is there, you can find that the client is screaming and complaining that he had already given his blood sample and he is not going to do it again. So what we want is that the lab personnel should be good at taking blood samples from clients so that he can get the correct results and know that he is doing well. “the challenge is present and it is quite extensive, and you can find that a client is complaining and raising his voice and refusing to repeat the test because he had already given his blood sample in the beginning and now he is told the test has failed. How is that?! Because you ought to give me the reason why the test failed.” Now those reasons are not given, now even if you were in my shoes you would not have agreed.

I: ok, ok, thank you participant number 5 for addressing that challenge, maybe I will invite participant number 4 then we will end there.

R: Number 4, it is true this challenge is present because, “It has happened to me before, I gave my blood sample for the test, then when I came next to the clinic for my results I was told that I need to give another blood sample. I asked them why because I had already given that sample and you are supposed to give me my results. They told me that they cannot locate the answers from that test. When they told me that I had to give another blood sample. “So this challenge is real. It is true that our doctors help us a lot but they must also be very keen.

I: ok, ok

R: Number 4, this is because when a person gives blood often, the first time, the second time and then he does not get his results on time, this discourages others. When you get your results they help you become more keen on taking your medication.

I: thank you so much participant number 4, now let’s close with participant number 7 so that we can continue with another question.

R: Number 7, I have just a few things to add on behalf of all.

I: ok, ok

R: Number 7, I am adding this on behalf of others, the major issue here is knowledge. Even those doctors and lab people you can find that it is not them that cause the challenge, you may find it Is the machine that is used to perform the test, maybe the blood sample has coagulated, and the machine cannot run a coagulated sample, so there may be other small challenges.

I: okay

R: Number7, the important thing is that the client who is going to redo the test should be educated on why he needs to redo the test, what happened until his blood could not bring results. You know, pricking a person is not an easy job because the client feels pain and not only pain but it is disturbing the client.

I: ok ok

R: Number 7, But also to add on the issue of language used when the doctors tell us that your viruses are asleep, this is also an area where education should be given out, because when a client is told that his viruses are asleep then the client will ask himself a lot of questions, because you know everyone has their own understanding. He will ask himself, “I have been told that my viruses are asleep, maybe I should stop taking the medication, for example some clients stop taking the medication, and others that when someone uses this medication when he goes for testing, a big percentage of the clients here are stable, when he goes to check his results and he is told they are asleep and you find that he is coming from his faith and he is coming from being prayed for, he seems to be cured while it’s all because he was told his viruses are asleep. Now there are statements that really confuse the clients because of the lack of education when they receive their viral load results. So if education is given it will help because many clients are perishing because they lack education on the viral load test. R.LINE

I: thank you very much participant number 7, he has spoken mainly about the challenge of lack of education, but isn’t education given often in this clinic or how is it given, on the issue of education what do you say? Welcome number 3

R: Number 3, Education is given but on the issue of viral load it is not given to the public because It is difficult. This is why those of us who get services at this clinic are not only adults but there are children and youth also. Youth are given education on viral load.

Question number 4

I: thank you very much, please lets continue. I would like to know when you are getting the viral load test how where you treated? Were you treated with respect, were you treated well or not, were you received by a doctor, did he treat you with respect. Addition from Dr. Peter, meaning that, did the Dr show you respect or did he just answer you anyhow because he had a lot of patients to cater to. How was the service given, how was the communication with the doctor, do not hide anything because it is part of the improvements for your benefit? Okay your welcome to give your inputs. Welcome participant number 5.

R: Number 5, so in my understanding, are you asking about the first time I got the test or?

I: Dr. Peter, anytime that you got this test, we are talking about the viral load test, when you go and get the service how are you treated, are you treated with respect or how are you treated?

Section B

R: Number 5, our service providers treat us well, we cannot say we are maltreated

I: Participant number 5 has told us that he is treated well and there is no maltreatment. Welcome participant number 4 so that we can hear your views.

R: Number 4, Surely they receive us well but sometimes it is that they are busy but about receiving us they give us a good reception and good service although sometimes the doctors are busy with the works they have.

I: thank you so much, we have heard participant number 4, he has said he is treated well but the service providers are busy, welcome participant number 7 so that we can hear your views.

R: Number 7, Just like my fellows have spoken, there are times when the doctors are busy, you find that there are so many of us and like now the season of vaccine(Covid-19) you find that maybe a doctor has come from a meeting and then one doctor is missing. Now that one doctor has to go here and there so he will disturb us because he will waste the time he was supposed to give service because he is also human. Those are the few challenges that we face but not so much, just those few.

I: Number 8, welcome now we hear your views.

R: Number 8, we are served well and we are received well.

I: number 3, welcome

R: Number 3, in addition, the services are very good, especially by the doctors, they remind us and you can find another client takes medication for 6months or 3months and the doctor reminds you that next month you have a test. We will not give you medication for 3months you will go back to taking medication for 1month until you take the test because we are human. “so if a client is given medication for 6 months he can get an excuse so the doctor in order to counter the situation will tell the client we will not give you medication for 6months we will give you medication for 1month so that next month you will come back for the test.” So when your test is done we will return your services to normal. I think in terms of services offered they are doing good.

I: ok ok and do they listen to you attentively?

R: Number 3, Oh even if you forget to come for the test they will call you to remind you that you should come and take the test so you come take the test and leave, although you have already been given medication. “Even yesterday, yesterday they sent me a text message to take care of my health which reminded me that I need to come for clinic. The service providers are good and even if you forget they must call you and remind you of the services you are supposed to get.

Section C

I: thank you so much participant number 3, now when you have gone to see the doctor were you allowed to ask him questions? Addition from Dr. Peter, and do they give to time to listen to you attentively? That perhaps today is your scheduled clinic and you have gone to see the doctor, do they give time to listen to you, so that you can tell him how you are doing? Number 9 welcome

R: Number 9, when you go in to see the doctor he asks you how your health is doing.

I: from Dr. Peter, so you also explain yourself?

R: Number 9, Yes.

I: ok, welcome participant number 1

R: Number 1, we are given time for him to listen to us but it’s not a long time, but we are given the chance.

I: okay so it’s not a lot of time, how much time is it maybe?

R: Number 1, when the Doctor is taking your sample you ask him questions and he answers you because there are many clients so he cannot give you just a seat down time listening to you. So we are not given a lot of time for him to listen to us.

I: thank you participant number 1. Welcome participant number 7.

R: Number 7, in short we are given time to be listened to and the doctors listen to us if we tell them what bothers us.

I: and when they were listening to you did they give you sufficient answers and that sufficed your need as a client? Number 5 welcome.

R: Number 5, when you go in to see the doctor he must ask you how you are doing and you explain, he listens ad while you are explaining he continues to question you. Truly we thank God because maybe if you as a client when you get to the doctor’s office and he asks you how you are doing you answer hurriedly that I am okay, what time you take your medication, I take my meds at 10… it depends on you as a client how you started the conversation with the doctor. But if you start explaining that I have so and so problem the doctor must ask you questions.

I: ok ok thank you so much, any one to add to that so that we can wind up, welcome participant number 4.

R: Number 4, to add is that I thank the doctors they treat me well and they must ask how you are doing with your health. If you answer that you are sick he will also ask you. And I was sick and the doctor took me to the test I was supposed to do. We thank God we live with them well. Surely they listen to us.

I: okay thank you so much for your views like you said you are all treated well and listened to. I would like to hear from you, what do you expect the service providers to improve, in all that we have discussed, what would you like to be improved? Welcome participant number 3

R: Number 3, to the service providers or?

I: From Dr. Peter, it is in the relationship between you and the service providers, is there anything to improve or should it continue as it is.

R: Number 3, hahaha improvements should be made at giving education about viral load, they should improve at giving results after the test and they should improve at collecting samples from clients they should not collect it twice, I think that is all in terms of improvements.

I: okay, welcome participant number 1 to give us your views, what are the areas you would like the service providers to improve when giving service.

R: Number1, they should improve on giving education about viral load, because you can find that another client is old, he does not know the meaning of viral load, they ought to be told clearly that this is a test that involves this and this. Because not all are educated and understand this issue of viral load. You can find that other clients are children and their mothers or fathers do not understand. So, I suggest that education be given to them because they will understand and when they go in to the doctors’ room, it is easy for them to know which test they are supposed to do and they can even ask the doctor questions or ask for the test.

I: thank you so much, maybe participant number 7 would like to add to that, about what you want to be improved.

R: Number 7, clients should be given explanation about the viral load test, that is, when the doctors are explaining they should give a detailed explanation on the importance of viral load testing. They should find a way that they will give this education from time to time. But the education should have emphasis on the new clients who are starting clinic. Also on the side of the elderly who have a challenge on this.

Question number 5

I: thank you very much, let’s go on now, how easy or difficult is it for you to get the HIV viral load test when you are told to get the test, how difficult is it for you to get the HIV viral load test when you are told it is supposed to be done or is there any difficulty or ease that you have come across? Welcome participant number 3.

R: number 3, I think there is some convenience because the doctor reminds you before time that in a certain month you have a test. So, now the challenge can rise at this point because if you tell a client that a certain month you have a test then for that month it depends on the patient location because some clients have jobs that require them to travel. There are other clients who live in Dar es salaam, but you will be surprised that he receives a call for work and leaves the region. But the ease comes when the doctor and client meets because the doctor tells the client directly that next month you have a test and the client says okay but when the date arrives you find that the client has travelled. Difficulty arises when the doctors forget to take the client’s sample. Another difficulty is when the client is unavailable through his phone.

I: okay

R: Number 3, because when the particular month for the test arrives the client must get the test, but when 2 or 3 months pass it is not good even for the clinic. There is difficulty when a client travels for work and there is difficulty in communication, when the client is unavailable over the phone.

I: thank you very much, welcome participant number 7.

R: Number 7, sorry, was that question asked for an individual or?

I: From Dr. Peter, it is just sharing the experience that you have or have seen with another patient or you have seen other patients facing the challenge. It is not necessary that you faced it, so he spoke well when he spoke of the experience of other patients that they travel, it is possible that he did not travel but he has seen other patients travelling and they failed to return on time. Now we wanted to know if that difficulty is coupled with cost, maybe someone is scheduled to come back but he does not have the fare or is it connected to distance, that he stays far, that he has touched slightly, that someone stays far and comes only once to the clinic or maybe someone is scheduled to come on a day that is not for taking medication. So tell us how those issues are, explain them to us. Welcome, I have seen number one raising his hand.

R: Number 1, “I will add to that, another client faces difficulty in his work place because a client enters work at 6 in the morning and he comes from work at 6pm, so when he decides to come for clinic he finds that it is too late and he fails to get the service.” The ease is because that client will be called and asked, “Please try and come, even ask for one hour off and come give your blood sample and leave.”

I: Number 7 welcome

R: Number 7, I think the difficulty is there, for the clients from Magomeni we find it easy because we are informed about the test before the date so when you come for clinic you come prepared psychologically, so it is easy. The difficulty comes when on the date the client is scheduled to come for the test he is not available, that’s where the difficulty arises. There should be improvement, maybe the client be followed at the area he is and his sample is taken it would really help. That is why there are many clients who miss their scheduled date for sample taking.

I: ok, ok, addition from Dr. Peter, I would like to know if the dates you are given are the same ones you come to take your medication or the month and date can fall outside of that. Welcome number 1.

R: Number1, There are other fellow clients who don’t afford the transport cost. He can tell you that, ‘I do not have the bus fare, I cannot come to the clinic’ So, you have to persuade him for some time until he attends the clinic”.

I: ok participant number 1 talked about clients failing to come for clinic because of the cost of transport. Welcome participant number 3.

R: Number 3, there are also challenges, some don’t have a transport fare, and accommodation fee, these challenges contribute to the problem. But there is also the challenge of some of the clients having clinic with more than two service points, for example, “a client may be receiving services at Magomeni for HIV and he is also receiving services at Mwananyamala hospital for Hypertension. So, you can find that on the date that he is supposed to give a sample at Magomeni hospital is the same date that he is supposed to receive a service at Mwananyamala hospital.” There is also the challenge of some clients leaving the service (lost to follow-up) which poses a difficulty in that service.

I: thank you very much, maybe if we look at the issue of the time of waiting to get that test, what do you say? Welcome participant number 7

R: Number 7, when it comes to time it really depends on the day, depending on when you come for clinic. It depends on how many clients are there and what kind of services they have for. It depends on whether they are experienced clients or new clients. Because in the case of new clients they have to be given education first, so when the doctor is giving education to that new client the rest have to wait, so it takes time but it depends on the day.

I: thank you, welcome participant number 3.

R: Number 3, to add to that like the sister here said, some clients are at work so you can find that in this center the clinic start at 8am so you find that a client is supposed to give a sample and to give results to those who tested is supposed to be given at 1pm.

I: okay

R: Number 3, so you find that at 1pm a client has come from the office for a short time, that is the opportunity he has to come for clinic and it is his date for the test but when he arrives, “he is told today was the date for giving his sample but you have come late so come again tomorrow, and if he comes tomorrow the situation is the same.” So my advice is that the time for sample taking should be extended because every client that comes here has his own challenges.

I: Number 5, welcome.

R: Number 5, I would also like to talk about this challenge of waiting to do the viral load test, the time should be extended. If they say today it Is from 6am to 6pm or from 8am to 2pm because the clients wait in que from 6am and when he finally arrives at the collection point they say you are already late. This challenge is present here and “it has happened to me before and I was so angry because I waited in que all those hours from morning and when my turn came they told me I was already late, and it is their responsibility so why should they tell me that I am late.” This is prevalent and we are suffering.

I: Okay, welcome number 7

R: Number 7, this challenge is prevalent at our center.

R: number 9, it is true this challenge is present.

I: thank you very much number 9, when we look on the side of the lab failing to run the tests, is there such a challenge or difficulty here. Have you met this challenge while you came for testing? Number 1 welcome

R: Number 1, it is true this challenge is present in our lab. There are times they can tell you that your sample is destroyed you have to repeat it, so you have to give your sample the second time. This is a serious challenge; the lab is not fit yet.

I: ok ok participant number 1 has said there is still a challenge in the side of the lab and there is a difficulty. Welcome number 3.

R: Number 3, there is also a challenge in the service providers, and this happens when you find that a service provider in a list of duty to attend patients is attending a seminar. So, you find few service providers left at the clinic who have to see so many patients. So, this challenge is present too. I: there is another participant who wants to add to that, number 4 I see you smiling, let us finish this.

R: number 4, challenges like those are present and the services are seen. Service providers should really help us because some of us are coming from far, we have a difficult life.

I: Yes

R: Number 4, but when we come here the service providers don’t know, they see it as something easy but they need to look at us differently.

I: okay, okay, thank you very much number 4, so if we leave the difficulties are there any conveniences that you have found in getting this test? Because I know it is not only difficulties you have come across, there are some whom everything has been easy for them is that right? Welcome number 7.

R: Number 7, “Conveniences are there because now days they don’t prick you several times in order to find a vein, no, they prick only once and put their devices on you it becomes easy to take your sample.”

I: okay, ok, maybe we continue now, is there someone among you who has not done the HIV viral load test in this year? Welcome number 6.

R: Number 6, I have never missed it because when your date reaches you have to do the test.

I: Welcome number 3

R: Number 3, I agree with the former speaker, when your date reaches you must take the test and the doctors must send you a text message or they must call you but there is another challenge I want to add.

I: that is on testing in this year? Ok you can finish up the challenge you have come across.

R: Number 3, it is out of the scope of what we are talking about but I just wanted to mention it.

I: we will give you a chance later on. Ok

R: Number 3, there is another client who is called by the clinic and he knows the importance of that test and the medication but the client says he does not have transport money and when you observe it is true and he needs to come for the test. I wanted us to look at that.

I: Okay

R: Number 3, if the client says that he doesn’t have transport fare then medication should be taken to him or transport money should be sent to him. So that he can come and take the test. Some clients really need the service but he doesn’t have the financial ability. So, I think that these kinds of clients should be considered so that they can continue with clinic. It’s either they should be supported on transport cost, or medication should be taken to them. Alternatively, if there is a center that is closer to him then the doctors of this center should communicate with the doctors of that center so that the client can give the sample at that center. This can also be used for those clients who want to keep their conditions a secret. Doctor should call the client and ask where he has travelled to so that if he is close to a health center in the region he has gone to he can go and do the test there.

I: Okay

R: Number 3, the client ought also to go to that center and talk to the doctor and ask him to talk to a doctor from this clinic so that he can get the services because our id numbers are everywhere, so the doctor will check the id number and take the sample for testing so they can check how he is doing. Please may this challenge be addressed.

I: ok ok

R: Number 3, Please I implore you this issue should be addressed. If there is a client who did not get to come to the clinic, then the doctor should try and follow him and give him the service.

I: ok thank you very much participant number 5, let us move on to others, is there another client who has not done the test in the period of one year, welcome number 1

R: Number 1, I have done it.

I: Number 4

R: Number 4, I have also done the test but I have not seen my results.

I: Number 4 did the test but the challenge was that he did not get the results. Addition from Dr. Peter, what month was that?

R: Number 4, it was in June.

I: From June until today the results are not back?

R: Number 4, I have not been shown my results, maybe because I was supposed to be told your results are so and so, but the problem is they write in the card but I remember when we tested CD4, once you have given the sample when you come for clinic you find that your results are already written in your card so as a client you know how you are doing. So in this viral load test the same thing should have been done.

I: okay, okay

R: Number 4, because I cannot read from my file or I cannot access the file so then if the doctor forgets or has a lot on his mind it is not easy for him to tell you that your results were such and such and you should do this and that.

I: Add from Dr. Peter, have you ever reminded him of your results?

R: number 4, yes, for those first few weeks I asked but they told me that my results were not out yet that they will check the system for them.

I: thank you very much, so when we consider the part of testing

R: Number 4, if I am given my results early enough and check my viral load after 6months pass and I do the test again after 6months I will if the viruses have diminished or increased, this means that I can control myself, so I will know where I am lagging if it’s in taking medication or elsewhere.

I: okay

R: Number 4, so it is good for the client when they get their results back because you can know that in this month I had a viral load of such. I can also save my results in my phone when they are given to me, it will help me know that after every 6months I am supposed to get the test, this will really help.

I: add from Dr. Peter, okay, and luckily I see that most of you have gotten the test, although some of you have not received your results. So what challenge…

R: Number 4, You can find that for a client his viruses are asleep but you do not know how many they are, this is still a challenge. But if I know my results that in this month my viral load is such it is below or above 1000 and I come and test again maybe they have decreased or increased so this really helps.

I: add from Dr. Peter, now I would like to know, this challenge of COVID 19 if it has delayed your viral load tests or if it did not bring any problem. What is your experience during this challenging time of COVID 19 in connection with viral load testing? Are there any clients who failed to get this test, especially you peer coordintors, because of COVID?

R: Number 1, we do get the services but there is a lot of delay because you can find that a client arrives at the clinic and has to go test for COVID because the services are given at the same place. So you find that you have to wait until other clients get serviced first. So you find that there is a long que, so it delays us in getting the service.

I: ok number 1, add from Dr. Peter, does it delay you from getting your results or from getting the test done?

R: Number 1, a client is delayed in getting the test done.

I: ok ok, thank you very much participant number 1, who has said he was delayed in accessing some of the tests. Okay, welcome another participant. We have not heard from you number 6, welcome.

R: Number 6, no it’s the same as my fellows have said, the service is as they have said, you can arrive at the clinic to get services before you go in to see the doctor you find that another client has entered to see the doctor and he takes a long time to come out. So you ask yourself one client went in to see the doctor but what is he doing in there all this time.

I: okay welcome number 7.

R: Number 7, for example there is an instance that happened here, one client was waiting to access some COVID services, they start with vaccination so you find a client has both a viral load form and a COVID form. Now the client notices that its getting late and he has not yet gone in to give his blood sample, he folded those forms, left them on the bench and left. That means that he has missed the service, this shortage of service providers is a problem because the same service provider has to give service, write, it is a challenge.

I: thank you, welcome number 4.

R: Number 4, what we ask is that the service providers should be properly allocated, so that not the same service provider vaccinates anti-COVID, the same dispenses medication and the same one should listen to the client. That is just not right.

I: okay

R: Number 4, now what time will you listen to the client? that is when you will find that the client is listened in too hurry, the doctor does not get enough time to listen to clients. They must divide themselves, if one dispenses medication then he/she should only dispense and if the other one receiving client then he/she should only be receiving clients. So this is a challenge we really want to be addressed because it is present here at Magomeni.

I: Number 7, welcome

R: Number 7, this challenge is really serious here at our center.

I: okay, okay, thank you very much, so there is a shortage of service providers in this center.

R: Number 4, the same service provider has to dispense medication, the same one has to collect sample, the service provider has to look for all the files, is that possible? really! Is there going to be good services? This challenge is really serious here. I would really like them to look into this and also for the clients who come from distance.

I: ok ok thank you very much. Now let’s finish with participant number 4

R: Number 4, that is truly a challenge for example, today is my turn to see the doctor. Before I get to the clinic the service provider has already come to take me for vaccination. I refused, I told him before I go for vaccination I need to be well educated on it. I have to understand and not be dragged like a small child. so I refused and told him I am not being vaccinated.

I: Okay

R: Number 4, so I came back and continued with the clinic, I went in to see the doctor, I got my medication. Then this woman came, and she has an understanding and asked me questions. I told her that I was just dragged to go get vaccinated, so she educated me but I told her today I am not ready. So service providers sometimes want to rush us while that destroys their work. The service providers are few so they try to do two things at the same time.

Question number 6 Part A

I: ok ok, how long does it take from when they take your sample until they return your results, add from Dr. Peter, for example this client whose sample today, how long will it take to get his results? Welcome number 1.

R: Number 1, when he comes back for his next clinic he will get his results.

I: okay ok

R: Number 1, but it depends, you can be given your results straight and be told they did not run and as a client you will have to give another blood sample.

I: so you find that you are given your results in one clinic, ok thank you, welcome number 7.

R: Number 7, results are given that way because that is when the client is available but the results were ready even 2weeks before. So for the results to reach the client he has to come to the clinic because you can find the client lives far from the center so for him to come just to take the results and return is difficult. This is why the client is given his results when he comes for his scheduled clinic. So service providers have planned that if the client takes medication for 6months when he comes for clinic is when he will get his results.

I: thank you very much participant number 7, welcome number 3 for any additions.

R: Number 3, it is as participant number 7 spoke, this challenge could be solved if the results are back within a week and then by accident the results are destroyed. It is better if there was a system whereby if a client’s results are destroyed they look for him immediately to repeat the test rather than waiting until the next scheduled clinic. If the client is called to the clinic before the next clinic day, it will help him to get his results on the same date he was supposed to get them originally.

I: thank you very much participant number 3, is there anyone else who has something to say before we close on that question. Welcome number 5.

R: Number 5, like my sister said results are usually ready after 2weeks so if the results are invalid they should send the client a text message like they sent to me, so that he can come to the clinic and redo the test rather than waiting until he comes on his clinic date. This will help because when the client comes on his scheduled date he will get his results.

I: thank you very much number 5 for your views, welcome number 7 to add to that.

R: Number 7, I think they stop calling us because it seems like a disturbance, for example this week I have come it has costed me time and money also because most of us are employed at Arabs places and an Arab does not want you to miss work, so it is a challenge. You can find that you came for clinic this week and next week you are called again to the clinic that your results are not good, it is truly difficult.

I: welcome number3

R: Number 3, it is truly for his own benefit, when a client is told to come back for another test he will ask for his results and be told that they were destroyed so he can be called and be told that his results are invalid before the date he is scheduled to come for clinic. For example, let’s say a client takes medication for 6months, this means that the client will not come to the clinic until the 6months are up on the scheduled date so that is when you will tell him your results are invalid that is a challenge. I think it is better when a client’s results are out he should be called and told to come for another test. Or you can just ask the client which day do you have time to come so that they can take your sample so that on the next scheduled clinic date you can get your results.

I: add from Dr. Peter, so you mean you would like a better way to communicate with a client whose results are destroyed/invalid.

R: Number 3, this challenge is truly serious because as a client you have already given blood from your hand and while they are taking it, it hurts, then you stay six months and then come back and are told that your results are not there. It is better that you inform the client amicably just like you receive new clients, you ought also to convince this client patiently that their result is invalid so that they understand.

Question 6 Part C

I: okay ok, thank you, and in getting back your results, how well do they give back your results? Add from Dr. Peter: do they get time to explain to you your results. Welcome participant number 3.

R: Number 3, we are going back to what I said before, the service providers are many but it depends on the doctor that you meet and the answers he gives you, for example, “For example a doctor can give you a good feedback that your results are back and show that your viruses load is at zero, that they are asleep or are not seen but even if your viruses are asleep you should not stop using medication.” Or a doctor gives a client his results and just tells him you are at TND, so the client does not understand the meaning of TND.

I: ok, ok

R: Number 3, or a doctor tells a client that you are at a high viral load, the client does not know what high viral load is so there is a difference between those who give the service and those who give the results. So we go back to the same thing that the feedback to the clients is not good. So there are some clients who have not gone to school but have the education to ask that this I have not understood. For example, there are some clients who did not go to school but he has his own importance.

I: okay, okay, thank you very much, Add from Dr. Peter, thank you very much number 3 for your explanation. So let’s finish up, number 4 it seems you have a burning issue I can see you shaking your head, welcome, you can say a few words and then we move on.

R: Number 4, that is my only challenge because I give a blood sample every 6 months, some of us are given medication for 3months. “You know 3months are a lot so when you come for your scheduled clinic you find that the doctor has forgotten to take the sample and as a client you fail to ask him so you decide to take your medication and leave.” So if there is a possibility that when the results come out they send them to us even by text message. Because this schedule of 3 or 6 months is a long time so if the results are out they should send us a text message to tell us our results, it will really help us because as a client I can come back and repeat the test before the 6 months. Not that after 6 months when I come for my scheduled clinic is when they tell me there is no results because my sample was destroyed.

I: Okay, welcome number 5.

R: Number 5, “you can find that another client refuses and even shouts and say you will not take my blood.” So this is a serious challenge at our center.

I: okay let us finish with number 4.

R: Number 4, “For example I was getting these CTC services at Moshi, the services of there and here are very different, because if a client is scheduled for a blood test you go to the clinic and there is a specific book, you go and meet with him and he looks for your results, he writes them for you on paper then you go in to see the Dr.” but when I came here and started clinic I see that it is very different. GOOD EXAMPLE FROM MOSHI.

I: okay okay thank you number 4, welcome number 3.

R: Number 3, when you come for clinic you measure your weight, you see the doctor and you go and take your medication or go and do a test, so I think there should be on service provider who is special for giving back results to clients. This would help a lot because those clients who do not understand can get an explanation.

Question number 7

I: thank you so much, now what can you tell us about the health education on HIV viral load testing, how do you get it, is it really given? Welcome number 7. Add from Dr. Peter, First is the education given?

Section A

R: Number 7, The education is given.

I: Add from Dr. Peter, the education intended here is the one concerning viral load and not about other services, we have seen in other places that the emphasis is on taking medication but viral load is not talked about. I don’t know about this center, ok let us start with number 7.

R: Number 7, let me say something that is clear, in this season we have put emphasis on education about COVID 19, it is education that is frequently given and we have forgotten that other forms of education should also be given.(REPORTED BY PEER HEALTH EDUCATOR)

I: From Dr. Peter, so now the emphasis is on COVID-19, right? so what about other kinds of health education?

R: Number 7, they are totally forgotten, for example, if I stand and give education I will talk about CORONA, I will also emphasize that clients should come for clinic on their appropriate scheduled date, maybe I will also emphasize on taking medication at the appropriate time but education on CD4 and viral load especially at explaining them, although they are written in the file but there are still some customers who do not know this services.” But if you are giving education and explaining to clients and if you narrow down to this word TND in your card the meaning is that your viruses have decreased. And that this test test as low as below 20 at this point it stops measuring and give us these answers. “so if we explain to the clients in deep they will understand, this education has really been forgotten but in the past I used to hear that they do these tests.”

I: ok we have heard participant number 7 who has said that education on viral load has been forgotten and the emphasis is on education on COVID 19, participant number 4 welcome.

R: Number 4, On my side since I shifted to this clinic, in the beginning I would come every month but in June is when it was changed. That education on viral load is truly not given, there is nothing except you are told that when you move from here go and take the test there and after you have taken the test you leave and go on with your own plans.

I: thank you very much, you have heard participant number 4, welcome participant number 5.

R: Number 5, “since I have come for these services and gone to see the doctor he asked me if I have had a cervical test, he did not ask me how many viruses I had, he only asked me if I have done the cervical test? So about education it is truly not given, it should be emphasized so that we can properly understand.”

I: thank you very much participant number 5, participant number 8 what do you say about the issue of education?

R: Number 8, I have not yet got that education.

I: Participant number 8 also says he has not gotten that education on viral load, welcome number 3.

R: Number 3, No, education on viral is not given, no.

I: Number 7,

R: Number 7, we try and educate the clients but there is no education on that test.

I: welcome number 3

R: Number 3, for example when your scheduled date arrives they will just call you if you do not show up in the clinic but you will give them your reasons. But that clients came to the clinic in the morning and were educated is a No.

SECTION B

I: add from Dr. Peter, and are there any Posts that talk about viral load testing. On your information walls I can see posts on other things, are their posts that explain about viral load testing?

R: All together… there are no posts except on TB, CORONA

I: ok, let us begin with participant number 5.

R: Number 5, I can see that there are banners talking about TB, CORONA, and Dengue fever, those are the banners that we can see here, but no one among those speaking about viral load, I have never seen that.

I: ok, participant number 5 has also said he has never seen them and there are none, participant number 8 welcome.

R: Number 8, there are none and that is why I said I have never seen them. Because if they were there I would have also seen them.

I: add from Dr. Peter, so number 8 says that if he even saw a banner speaking about viral load he would be eager to read it/them but unfortunately there are no such banners.

R: all together: eee there are no banners that explain viral load testing.

I: welcome participant number 1

R: Number 1, there are no such banners, I have not seen them.

I: ok ok so we have seen that there are no banners that explain viral load testing, welcome number 5.

R: Number 5, they should help us with banners because they help us get a lot of information and learn more about viral load testing and the importance of that test. Also they should educate us concerning the importance of that test.

I: ok welcome number 6.

R: Number 6, I suggest that they even put a TV for us which will be showing different adverts on different diseases that we will watch and learn more about the diseases and their tests. For example, the Tandale center has a big TV but here at Magomeni there is not.

I: thank you very much participant number 6 has said that they should put a TV that will be showing adverts while they come for clinic. Welcome number 7.

R: Number 7, in the packages that we use there are channels, the government should form a channel that will be explaining more on HIV. Any client that comes to the clinic will find the HIV channel and will be learning about the disease.

I: ok ok thank you very much number 3 also wants to contribute so that we can finish.

R: Number 3, there are other people through different organizations ought to have a big contribution because when a client arrives here for clinic the clients just sticks to the doctor, ok, ee but the client does not know that there are some organizations that can help him get that education. It can be in a month some of the clients are taken to go and be educated because the doctor cannot do it by himself. Those organizations can help take those clients for training about those services.

Question number 8.

I: okay thank you very much for your views, we have reached the end now what should be done inorder to improve HIV viral load testing at this center, I know we have discussed a lot especially in the area of education, we have also discussed on the part of adverts, now what else can be done? Add from Dr. Peter, those that you want emphasized so that we can leave with them as researchers. Welcome participant number 1.

R: Number 1, the first thing I want to say is that service providers should be increased, so as to curb this ques. For example, a service provider dealing with CORONA should only deal with CORONA, If he is involved in viral load he should deal with viral load only.

I: okay okay

R: Number 1, because another client is coming from far, for example Gongolamboto or Chanika and he comes for clinic and to give his blood sample for testing. So the distance from home to this place he finds that the time for giving service is up.

I: thank you very much participant number 1 has said that service providers should be increased, participant number 3 welcome

R: number 3, improvement in giving education, emphasis should be added on education on viral load testing.

I: okay ok, participant number 3 has said we should improve on education, number 1 said service providers should be increased, now welcome participant number 4, what would you like to be done now in providing this service of viral load testing in your center.

R: Number4, my contribution is just that, the services should be improved, service providers increased, and the issue of time to get service, because some of us are the breadwinners of our families so you need to come to the clinic get your services fast and then leave to go and do other things like business, etc. but when you come here and find a que you are delayed.

I: okay okay. Thank you very much participant number 4, participant number 5 welcome.

R: Number 5, I suggest that services are increased, that is, if a service provider has the job of sample collection then he should stay there, the one who is in charge of medicine dispensing should remain in the same, and not that one service provider should be involved in everything, no. every service provider should remain in his section.

I: okay, okay, let us finish with participant number 6, welcome

R: Number 6, hahaha I have nothing to add different from what has been said.

I: thank you, welcome number 7

R: Number 7, thank you, the only thing to add is about these people who come for testing at this center.

I: who are they, the transporters?

R: Number 7, eee yes them, a good percentage of the service providers try really hard that al the clients with a test should have given their samples by noon, but the time they come to pick those samples should be increased because many of us here are employed and we know the challenges of being employed. There are a lot of fellow clients who we used to communicate. You may find someone telling you that, for him/her the right time to come for clinic is 6pm. So the center should prepare 2people who will volunteer to work until evening so that those clients can come in the evening and access the clinic services.

I: Add from Dr. Peter, what is the maximum time for one to come and give his sample here?

R: its 12 noon.

I: From Dr. Peter, 12 noon

R: Yes that is the end, you will be told that the end Is 12 noon, but also sometimes some of the service providers will not say what is the deadline, but if you see that person who picks the samples and you have not given your sample then you know that is it for today.

I: From Dr. Peter, once the transporter arrives, if you have not tested then you will not be able to test again.

R: Once he has passed you can no longer test again until tomorrow or another date.

I: okay thank you very much number 8, welcome

R: number 8, there is nothing extra from what my fellows have spoken, they should add the service providers because there are times you can come for clinic and you wait a lot because you have come for clinic to get a quick service and go back to your duties.

I: okay, thank you, participant number 5 welcome

R: Number 5, eee for example you are working at an Indian’s place and he says he is giving you one hour to come for clinic but when you arrive here you wait for more than 2hours it means when you go back to work you will not have a job. So the service providers should be increased and when they are many it will help us to get the services quickly and without getting tired.

I: okay, okay, after seeing what should be done in this center now I would like us to look at the district and nationally what should be done. Welcome participant number 3.

R: Number 3, first we would like to thank you because this service did not only start here, the services started somewhere else, so as the days proceed clients are increasing in number and the areas where this service is offered are few and the place to store the files is also small. It is possible that a client arrived a long while ago but his file has not yet been located. For example in other hospitals you can find that they have arranged the files in an orderly manner, the data section is on its own and the file section is on its own. Service providers have their place, so the district should increase the space where this service is offered. Even in the area of testing, it should be improved so that clients can bring their close associates to also get tested.

I: thank you very much, now nationally, what do you advise to be done. Welcome participant number 7.

R: Number 7, Nationally also as we saw in the records during AIDS day celebration in December, they said that infection has risen and they are not falling, this means that government target will not be reached.

I: okay, okay.

R: Number 7, now inorder for us to reach that target I think in the time we started taking medication 2008, but there were a lot of challenges more than now. Nowdays discrimination has reduced and other things have decreased but still we need to invest a lot more in the side of education. For example in those areas with a lot of people for example in the buses we suggested that there be drama showcases which explain on the things one should do to prevent this disease. The government should invest on this issue, and also clients who use these medications should be motivated.

I: okay okay

R: Number 7, if we manage to motivate these clients, motivation will increase in them and they can even start looking for their partners and bringing them for clinic, we can also motivate them through education so that they can also be used to motivate their fellows. For example there is a day I was going through the streets, “I found people arguing, it was on the AIDS day it was at night. They were saying that if you take your medication correctly you can not infect someone else, even if you sleep with a woman you can not infect her, nowdays people are living well.” It is a good thing but I asked myself because the topic was started by someone who does not know, there some who objected. So if education is spread to people properly the government will reach its target quickly, but if education is not given as it was in the beginning.

I: okay okay, so I would like to thank you very much for giving us your time and we have reached the end of our discussion and the wonderful views you have given us like we told you in the beginning, we will take your views and go and work on them, okay eee

R: All, Okaayy

I: so I welcome you if anyone has anything to add welcome so that you can add it. From Dr. Peter, we can add If there is anything because when we interviewed the service providers they pointed a finger at you that they give you a date for coming to test your viral load but you do not want to come. They did not speak of education or anything, they said you are given a date for testing but you donot show up.

R: Number 4, what about me, my file was written the wrong date, they wrote the wrong date, they told me to come back on the 5^th^ of June but because my medications were finished and I know my scheduled date I came to the clinic, I have the card here and they did not write a date. But because I know my responsibility and my medications had run out I came for service, I came today.

I: okay okay

R: Number 4, and they did not call me or anything.

I: thank you number 4, is there anyone else with something to add.

R: Number 4, if there is a possibility you should give us your phone numbers hahaha.

I: Number 7 welcome

R: Number 7, now I see as if the investment on AIDS has decreased, as if it is now a common thing so the investment has highly reduced. If we look at the energy of mobilizing it has also reduced not like in the past. In the past there was a lot of investment that is why we are like this today. Because in the past clients would come wearing full hijab but nowdays there are people who come forward and declare that they are living with this problem. We are truly thankful to the government it is doing its best. Education should be given to all sides, to those who get pregnant also, because there are people who think that if you get AIDS you can not give birth. Also the government should go into churches to give education on this problem. Clients should be educated on what TND means so that when they are tested and get their results they can understand that if I take my medication correctly it helps.

I: okay welcome number 5

R: Number 5, I would also like to add on that, service providers do not come to give you a test

I: From Dr. Peter, I would like to correct something that the service provider schedules a date for you to come and take the test but you as a client donot come but you come on the date for taking medication.

R: Number 5, ohoo, so its like that, well I understood incorrectly.

I: well I want to thank you very much , I thank Zenais for leading the discussion well for asking you questions, and she has covered everything. We are going to take this information and we will analyse them with the those from other centers because we have 6 centers this is one, there is Amana, Tambukareli, Mbezi beach of Kimara and there is Mbagala, so we will go even there and get data then we will analyse them together and present our findings. We will send the report to stakeholders who make policies, TACAIDS , MDH, and I will present on the AIDS DAY next year if God keeps us alive.

I: I also thank you for your participation and your time. I wish you all the best.

R: All together, okay

I: Thank you.
