# Supplementary material for: Patient reported experience measures on HIV viral load testing at public health facilities in Dar es Salaam, Tanzania: A convergent mixed method study
Source: PLOS Glob Public Health. 2023 Apr 7;3(4):e0001024. doi: 10.1371/journal.pgph.0001024 (PMC10081772; doi:10.1371/journal.pgph.0001024)
Supplement: S3 Data — (ZIP) [file pgph.0001024.s003.zip › S3_Data_FGDs transcripts (word documents)/Mbezi Dispensary FGD.docx]

PATIENTS FOCUSED GROUP DISCUSION (FGD) AT MBEZI DISPENSARY

**INTERVIEW DATE: 27- December- 2021**

**DEMOGRAPHIC CHARACTERISTICS**

| **ID** | **Age** | **Sex** | **Marital Status** | **Education Level** | **Source of Income** | **Duration (Year in Care)** | **Residence** |
| --- | --- | --- | --- | --- | --- | --- | --- |
| **1** | **42** | **F** | **Single** | **STD Seven** | **Small Business** | **5year** | **Mpiji Magohe** |
| **2** | **35** | **F** | **Single** | **Form Four** | **Small Business** | **6year** | **Mbezi Luis** |
| **3** | **33** | **F** | **Single** | **STD Seven** | **Small Business** | **6year** | **Kibanda cha mkaa** |
| **4** | **48** | **M** | **Single** | **University** | **Small Business** | **7year** | **Msuguri** |
| **5** | **40** | **F** | **Single** | **STD Seven** | **Small Business** | **7year** | **Kibaha** |
| **6** | **39** | **M** | **Single** | **STD Seven** | **Day worker** | **6year** | **Kimara** |
| **7** | **47** | **M** | **Single** | **STD Seven** | **Volunteer (Expert patient)** | **6year** | **Mbezi Msakuzi** |
| **8** | **64** | **M** | **Married** | **STD Seven** | **Small Business** | **21year** | **Kimara** |
| **9** | **37** | **F** | **Married** | **STD Seven** | **Small Business** | **6yaer** | **Mpiji Magohe** |

I: Hello, how are you?

R: All, Fine

I: welcome, today is 27/12/2021 I am at Mbezi center and I am having a discussion with some of the clients that attend this clinic. So you are welcome please feel free. My name is Zenais Anthony Kiwale I work at NIMR Muhimbili and today I am here to have this discussion with you so as to learn from your experience on issues that will help to improve HIV viral load testing services at this center. So, I will guide you through the questions that will help us get your views on how you have been accessing this service. Everyone here will contribute to this discussion. If there will be someone who has not understood the question, please feel free to ask so I can clarify. Ok?

R: All together, OK

Question number 1

I: so, let us start with the first question, since all of you are registered and are receiving services from this center, I would like you to tell me what kind of services you are supposed to receive from this center? What are those services that you are supposed to get? Welcome participant number 1.

R: Number 1, weight measurement, taking medication and getting a return date for when to come back to the clinic next.

I: Okay, ok participant number 1 has said the services that he receives are weight measurement, taking medication and getting a return date so he can come back for another clinic, okay, thank you participant number 1. Welcome participant number 9.

R: Number 9, Thank you, we have been testing for cervical cancer for women, we have done blood tests, and they tell us our treatment progress.

I: okay okay, we have heard participant number 9, she has said that there is testing for cervical cancer and blood testing and being told of your health progress, okay. Welcome another participant, welcome participant number 6.

R: Number 6, on my side the services that I get, like number 1 said, it is that way, but personally there is another service I got. I got an infection but I thanks to the clinic staff, they made efforts for me and I recovered, as you can see now I am very thankful.

I: okay okay, we have heard participant number 6, she has given us her views, welcome participant number 8.

R: Number 8, weight measurement and HIV viral load testing

I: we have heard participant number 8, welcome participant number 2.

R: Number 2, the services we receive here are like my fellows have said, and I am very happy with them. There is weight measurement, blood test in order to see how we are progressing with our health and changing of medication, this has also made me happy because the previous medication used to make me drowsy but the current meds, you just take them like you would be taking Panadol, so I am very happy.

I: okay okay, thank you, we have heard participant number 2 how she has shared about the services she gets when she attends this clinic. Welcome participant number 4, I saw you raised you hand.

R: Number 4, the services we get here are good, we are given advise and there are other times we forget to test for viral load but the service providers usually remind us, and give us advise and serve us well.

Question number 2

I: okay, okay, so after looking at the services that you get, there is a service you mentioned, HIV viral load testing, now what do you know about HIV viral load testing? What comes into your mind? Welcome number 4.

R: Number 4: About HIV viral load testing? well, when we use medication we are supposed to test so as to know if those medications are working. In the other words, the test is to see if a client is using ARVs correctly, like taking at the appropriate time, that is why we test HIV viral load.

I: okay, okay, we have heard participant number 4, welcome participant number 6.

R: Number 6, that is truly something that helps one know the progress of a client who is taking and using medication because one can take medication but not be able to use it on time. So we have to do that test so as to know our viral load, are they asleep or not and if they are increasing.

I: thank you very much, we have heard participant number 6, she has also given her views that we have to take the test in order to know how we are doing, thank you very much. I welcome another participant, what does that test involve? Welcome participant number 7.

R: Number 7, in order to know how you are progressing with your medication use, whether you are using your medication correctly or if you are making the mistake of skipping, in order to know that the viruses have increased or they are sleeping. So as to be instructed on how to use medication correctly.

I: thank you participant number 7, we have heard her, she has also given us her views. Is there anyone who would like to add to that or should we move on?

R: All, Let’s move on.

SECTION C

I: okay okay, now how often is this test supposed to be done? Welcome number 1.

R: Number 1, in a year this test is supposed to be done twice.

I: thank you very much, welcome participant number 4.

R: Number 4, this test is done twice a year, after every 6 months a client is supposed to take the test again.

I: thank you very much, so every after 6 months a client is supposed to take the test again, we have heard participant number 4, welcome participant number 5.

R: Number 5, it is done twice a year, like my fellows have said, that is after every 6 months they take your sample, so in a year they take your sample twice.

SECTION D

I: thank you very much, now who initiates first to talk about the need to do that test? Welcome participant number 3.

R: Number 3, I think that the doctor is first.

I: Participant number 3 says the doctor is the first to advise her to take the test. Number 4 I saw you raising your hand, welcome.

R: Number 4: Basically, it is the doctor who decides to test, but even a client is supposed to remember the date for his/her test because, the doctor can also forget and can skip reading in the card section that indicates the test date. But if as a client remember, even if the doctor forgets you ought to remind him that it’s the date for the test.

I: okay, thank you very much, welcome participant number 6.

R: Number 6, I am saying the same thing as my fellows, there are times the doctor can forget that a client is supposed to take a test, but if you remind him, because the doctor can forget or I can forget, so that is both our responsibility.

I: thank you very much, we have heard participant number 6, she has said that it is the responsibility of both of you, client and doctor to remind each other so as to get the test when you come for clinic. Okay, welcome number 9 to add to that.

R: Number 9, in reminding each other about the test, the first thing is that the client is supposed to be first because the doctor has a lot of things, he sees a lot of clients and there are times he may forget because his head has a lot of things, but as a client you need to care about your health and remind the doctor that I came on a certain date of a certain month and today I am supposed to take the test for the sake of my health. The first thing is the client caring for himself.

SECTION E

I: okay thank you very much, we have heard participant number 9, she has also said that a client ought to remind the doctor about doing the test. Now what is the reason why we keep doing the viral load test, is there any reason? Number 9, welcome.

R: Number 9, the reason is like we said before, you test to know your health progress because you are taking medication and taking them on time. For instance, I have witnessed many clients who don’t take the medication appropriately, there health shows vividly that they do not follow the prerequisites of taking the medication.

I: Okay

R: Number 9, this means that that client does not often follow the rules. The doctor can presume that a client uses medication correctly but find that he does not use it correctly. So the doctor does the test and brings the results to the client.

I: okay.

R: Number 9, it makes easy for you to know whether you are failing treatment and identify areas to improve.

I: okay ok, thank you very much, participant number 6 I saw you raised your hand, welcome.

R: Number 6, on my side, I think clients ought to follow the rules of the medication we are given. But also in the time we are given those rules we also ought to come and do the blood test so as to know our health progress.

I: okay ok, thank you very much participant number 6, welcome participant number 2.

R: Number 2, on my side they take that sample in order to know if I have contracted a new infection or if the infection is still the same, that is, if the viruses are asleep or if they have increased by getting a new infection.

I: okay ok is there anyone else who would like to add anything? participant number 3 do you have anything to add there?

R: Number 3, No.

Question number 3

I: Well, now what do you think is the importance of getting the HIV viral load test in your clinic? First is there any importance, welcome number 4.

Section A

R: Number 4: This is very importance because when you test you can know your treatment progress, because you will know whether the medication is working in your body or not. The good results give hope and trust to keep on using the ARVs when you see that the viruses have been suppressed and your CD4 has increased so you feel health like anyone else.

I: okay thank you very much, we have heard participant number 4, participant number 5 you raised a finger, you are welcome.

R: Number 5, the importance of testing is there, you get to know if the medication is helping you or not.

I: okay thank you, welcome participant number 7.

R: Number 7, my views are not different from my fellows who just spoke, it is important to do the test so as to know and contain the number of viruses.

I: Well, thank you very much participant number 7 talking about the importance of HIV viral load testing. Number 8 maybe you’d like to add something.

R: Number 8, No it is the same thing.

Question number 4

I: Hahaha, well it’s the same thing! Some of you have managed to do the HIV viral load test, okay. Can you tell us how have you been communicating with the service providers? Refer to the last time the sample was taken till the results shared? Welcome number 7.

R: Number 7, our communication is mainly through written patients CTC card, but if I have a phone number, and tomorrow is my scheduled day to come for the test, they would call to tell me about it.

I: thank you participant number 7, we have heard her, she says that she is reminded by telephone call. Participant number 6 what do you say about communication.

R: Number 6, communication is there, if a client forgets the service providers call you or text you to remind you of your clinic date.

I: thank you very much participant number 6, welcome participant number 4.

R: Number 4, communication is usually good so even when the date for you test arrives they usually call you and remind you that you are supposed to come for testing, if you are supposed to test for CD4 or if you are supposed to test for viral load, so communication is good.

I: okay, okay, welcome participant number 9

R: Number 9, communication is good because there are times you are supposed to come for viral load testing but it is not your date for taking medication. What is done is that the service provider give you a call, because when they check your file they can see that the date for taking medication is different from the one of testing. So what the service providers do is they call us and tell us that the date is approaching.

I: Yes

R: Number 9, Maybe it’s on Monday or Tuesday so they call and tell you that on Tuesday you are supposed to come and do the test.

SECTION A

I: Okay ok, thank you very much participant number 9, when we look at communication we also look at the language used, what language is used, welcome number 4.

R: Number 4, so basically when we say that communication is good we mean that the language that is used is also good because if the language is not good then there is no good communication.

I: okay okay, thank you very much, is there anyone who has had some challenges, let’s talk about the challenges you have faced when communicating when you are supposed to come for viral load test. Number 1 would you like to start, ahahah welcome number 3.

R: Number 3, there are no challenges

I: okay welcome number 7

R: Number 7, on my side I have not faced any challenges when it comes to communication.

I: thank you number 7, welcome participant number 8, let’s hear your views.

R: number 8, I too have no any challenges.

I: okay welcome number 9

R: Number 9, I once faced a challenge when I was sick on the day when I was supposed to come for clinic but I was sick and at the same time I was supposed to do the test. “I tried to send someone to come and take the medication for me but the challenge I faced was that the person I sent was not allowed to take the medication for me. He was told that I ought to come myself because I have a test. At the same time, I was very sick, so I had to be carried and brought to the clinic while I was sick so that I can do the test and take medications.”

I: I’m so sorry, so at that time were you supposed to take medications only or also do the test?

R: Number 9, I was supposed to take medication and do the test so I tried to ask them to give me medication first and then when I get better I will come for the test. But it wasn’t possible they said I must come to the clinic, so it gave me a hard time because I was sick and when I asked them they refused so I had to come.

I: So sorry, participant number 9 has shared her challenge with us. Welcome participant number 5.

R: Number 5, I also have faced a challenge, I usually bring my child to clinic but there was a day he was sick and he was on medication at home so I had to come and take the medication for him, but when I got to the clinic they told me that the child is scheduled for a test, I told them that the child is sick.

I: Yes.

R: Number 5, So I thought instead of coming with him and the way he is sick I saw that it is better that I come and take the medication for him and then when he gets better I will bring him for the test. So they refused to give me the medication they said I have to go and bring him.

I: Okay

R: Number 5, I had to go back home and plan for the next day to bring him so that he can do the test.

SECTION B

I: okay ok, thank you very much for sharing the challenges that you have faced when you came for clinic. Is there anyone else who has faced a challenge, others said they did not. Now let’s look at the issue of being served with respect, what do you say, are you served with respect, without being maltreated? Welcome participant number 9?

R: Number 9, sometimes there is a language that can be used and it can annoy a client because you can arrive at a time when the service provider who is supposed to take your sample is busy with her work and at the same time as a client you are supposed to be served. So she will tell you, “Why don’t you seat there and wait” and you can find that she is not doing anything she is just seated. As a human being it hurts you because you do not expect to get such an answer from a service provider.

I: Yes

R: Number 9, because even if you were doing something else that has no importance when a client arrives you are supposed to give him the service and then you continue withother things, but when you use words like, “just seat there” so when a service provider gives such strong words it is not a good thing.

I: okay, okay, we have heard participant number 9, the challenges he has gone through. Welcome participant number 4.

R: Number 4, I think the services are good, I have not met any challenge.

I: okay thank you participant number 4, welcome number 6

R: Number 6, on my side since I started getting this service there are certain services that were alittle bit bad. When you arrive here inorder to get services you can encounter a service provider who shouts at you like you are a small child, but for now that issue is no longer there.

I: thank you very much participant number 6

R: Number 6, on my side, I do not know for others, because there are times that you find we are not ok.

SECTION C

I: It is true because today you can wake up in a certain mood and most days we are not ok. Thank you very much for your views. Now in what ways did the doctors, nurses or service providers listen attentively to you when you came for service especially for this viral load testing. Did they give you the opportunity to ask them questions and did they explain? Welcome number 6.

R: Number 6, on my side I can say that they listen to me because when I tell you that when I started using this medication there are things that happened and I have got a lot of hope on treatment.

I: okay okay

R: Number 6, As for me, before I started medication, I had a certain infection but after I started using the medication and the doctors were serving me well, the service Is truly good, I am doing very well. Also when I came to the clinic this time they said I cannot get medication unless I am vaccinated for COVID 19, but I told the Dr that I have an infection and even you Dr know this, so how will that be? So he called his colleague to tell him the situation, and he told him that there was no problem for me to take the meds while unvaccinated. So I am the one who asked them not to be vaccinated. So I think the service is good because they listened to me.

I: okay ok, thank you very much. Welcome participant number 1.

R: Number 1, they serve me with respect because when I come for clinic they ask me how are you feeling, they ask me if I am sick, they give me a chance to talk and they listen, and I was also given good answers to the questions that I asked.

I: we have heard participant number 1, you said you were given good answers that were sufficient.

R: Number 1, Yes

I: okay, thank you, welcome number 4.

R: Number 4, I was also given a chance to ask questions and they listened and they answered me well.

SECTION F

I: thank you very much participant number 4, i welcome another participant so we can finish this part and go on to another question. I don’t see other participant to add to that, okay. So In all that we have discussed, what things would you like the service providers to improve? Welcome number 8.

R: Number 8, I think there is nothing else because the tests are available and when I come for clinic I get them.

I: thank you participant number 8, welcome participant number 7.

R: Number 7, what needs to be improved is the services on the side of communication because the service providers do not call us, because a client can forget to come to the clinic in the scheduled date, for example I was supposed to come for service on the 24^th^ but when I remembered and checked my card I found that the date I was scheduled for had already passed. So I thought that they would have reminded me.

I: Okay, ok, so as participant number 7 explained to us. Welcome participant number 6.

R: Number 6, My advice Is to improve in the area of communication, but secondly is to just remind them that when you are a civil servant you need to have a hobby in the thing you are doing, but there are some who do not have a passion with the job they are doing because of money. That is not a good thing, the service providers should try and be close to us clients that will help us get good service.

I: okay thank you very much, welcome number 2

R: Number 2, thank you very much, I think the service providers should just improve on love.

I: Thank you. Welcome number 5.

R: Number 5, I think like my fellow said here, the thing to improve is love, because if the service providers serve us with love they will serve us well and we will get good service. “Because in the past when your clinic date approaches you start thinking, how will I go for clinic because the service providers were very harsh, as a client you ask yourself questions whether to come or not.” But I see that they have stopped being harsh.

I: thank you very much, let’s finish with participant number 2 then we go on to another question.

R: Number 2, Like the lady over there said, the service providers should continue to call us and show us love. Because when they call us we know for sure that they care about us and remember us.

Question number 5.

I: okay ok, thank you for your wonderful views. Now I would like to know how easy or hard is it for you to get the HIV viral load test when you are told to do so? Let’s start with the ease, welcome participant number 7.

R: Number 7, in getting this test for HIV viral load on my side there is no hardship at all because the service is available.

I: Welcome number 3, let us hear you too.

R: Number 3, I also think it is easy, there is no hardship at all.

I: thank you, she has also said there is no hardship at all. Number 9, welcome

R: Number 9, I think there is no hardship at all because, when you are needed to take the test the tests are available on the same day.

SECTION 1

I: okay, ok, when you said that there is no hardship that you face, now I would like to look at the side of cost, what do you say, is there any cost that you face when you come to get the service here at the clinic? Welcome participant number 4.

R: Number 4, there is no cost that I incur, it is just your availability as a client to take the test.

I: thank you particiopant number 4, welcome number 9

R: Number 9, I also say there is no cost at all because there is no cash that we give in order to get the test, maybe the cost we can mention is time. It is just upon the client to give his time to come to the clinic so as to get the service. But there is no cost to the test.

I: so there is no cost to the test, welcome participant number 1.

R: Number 1, There is no cost because the test is free.

SECTION II

I: The tests are free, okay, ok, and what about the distance, by distance I mean that I know that each of you live at different places, what do you say about the distance of coming to this clinic? Please let us speak loudly because it is raining, so that we can hear each other. Welcome participant number 4.

R: Number 4, we cannot really talk about the distance because most of the time it is the client who chooses which clinic he wants to be served at, so the client can choose to get the test at this center and other services get at another center. So I do not think distance is an issue.

I: okay, ok, number 4 says the issue of distance is not a problem to him, welcome number 9.

R: Number 9, I still have the same answer as number 4, that a client is not forced that he has to come to Mbezi because you can take your medication at any center that you feel is easier for you in relation to where you live.

I: Yes

R: Number 9, so even for the one who came from Kimara to Mbezi center it means this is where he finds it easier for him to come for clinic. So on the issue of distance I think there is no problem because the center for getting service is the choice of the client.

I: thank you very much participant number 9, is there anyone who wants to add to that, welcome number 2.

R: Number 2, on my side the issue of distance is not a costly issue because, it is the client who chooses where to get service because there are some clients who live in Kimara who do not go to Kimara hospital but opt to come to Mbezi for service.

SECTION IV

I: okay, thank you very much, and what about the date for the test, is it different from the date for taking your medication? Have you faced such a thing? Welcome participant number 9.

R: Number 9, it has happened many times, it has even happened to me. It has been that way not because of their mistake, It depends on when was the first time you got the test, and how your current health status is.

I: okay okay, welcome participant number 7

R: Number 7, on my side I have never met such a challenge.

I: thank you, welcome participant number 6

R: Number 6, that challenge does exits and it happens but it has never happened to me since I started attending clinic.

SECTION V

I: thank you very much, now what about waiting time, when you have already been scheduled to come for the test and the date arrives and you come here, how long do you wait, Is the waiting time a challenge? Welcome participant number 9.

R: Number 9, about waiting time for me it has never bothered, because if the doctor has arrived in the sample collection room it does not take time. Once the doctor has arrived he calls you and you enter the room, he takes your sample that very moment it does not even take 5 minutes, he will take your sample and you will leave. So in the matter of time, on my side, there are no challenges at all.

I: okay thank you participant number 9, welcome number 7, let’s hear your views.

R: Number 7, on the matter of this test, as my fellow said, I think it does not take a long time.

I: how long does the test take?

R: Number 7, it does not even take one hour, it can take a few minutes.

SECTION VII

I: thank you very much. Are there any challenges that you face with the service providers when it comes to that test? Welcome participant number 2.

R: Number 2, I have never faced any challenges because on the dates that I had the test, I come for the test, I meet the service providers and I got the test done as usual.

I: thank you, and what about the challenge of lacking equipment for taking the sample? Welcome number 4.

R: Number 4, I have never faced such a challenge.

I: Welcome number 6.

R: Number 6, I too have never faced such a challenge.

I: thank you very much number 9 I saw you raise your hand.

R: Number 9, I want to add that the equipment are always available, so you do your test and leave.

SECTION B

I: okay, thank you very much, we have said there is ease in doing the test, the test takes a short time, right? So now please tell me, what made it easy for those who got the test, what made it possible/helped? Participant number 4.

R: Number 4, I think that nothing really helped apart from you as a client queuing, getting into the office and then you leave.

I: Number 6 what do you say?

R: Number 6, when you get to the center you wait for your fellows who you found to get the service and then you go in, give your sample and leave.

I: thank you, welcome number 9, you too raised your finger.

R: Number 9, I think the thing that makes it easy is that when it is your date you come to the clinic, you meet the service providers and then you go in to give your sample and leave.

I: thank you very much, in this year, is there any client who has not done the viral load test? Welcome number 3

R: Number 3, I have got the test

Number 1, I have also got it

Number 2, I have also done the test, twice

Number 5, I have also got it

Number 6, I have also got it.

I: Welcome number 8, let’s hear you. Have you done the HIV viral load test this year?

R: Number 8, I have also got it.

SECTION C

I: Now let’s look at the challenges of COVID 19, I would like to hear from you, did this challenge hinder or stop you from getting the HIV viral load test? Welcome participant number 4.

R: Number 4, in that time the tests were going on as usual.

I: thank you, welcome participant number 1 and let’s hear your thoughts. Was COVID 19 one of the reasons that this test was not done?

R: Number 1, No, the tests were available as usual.

I: thank you participant number 1, now welcome participant number 6

R: Number 6, there were no challenges, the service continued as usual.

I: okay, ok, thank you very much participant number 6 has also said that he did not meet any challenge in the area of testing, welcome number 9.

R: Number 9, I think there was no challenge because the services were continuing as usual.

Question number 6

I: okay, thank you very much. You have said that all of you have done the viral load test this year, now I would like to know, how long did it take to get the results of that test since the day they took your sample? Welcome participant number 9.

R: Number 9, about when you get the results it depends on when you got the test done. If you take your medication after every 3 months then after 3 months you will come and find results. Or if you are scheduled to take medication after 1 month then when you come back to the clinic on your scheduled date.is when you will get your results.

I: okay welcome participant number 1.

R: Number 1, if you give your sample for the HIV viral load test on the day you come for clinic, when you come next for your clinic to take medication you will be given your results.

I: okay, is there anyone else to add to that? Number 3 what do you say?

R: Number 3, the answer is the same, I don’t know when it made available from the lab. But if you are given medication for 3 months, once you finish those medications and you come to the clinic for the next dose that is the day they will give you your results.

I: We have heard participant number 3, she has also told us when she is supposed to receive her results. Is there someone else who wants to add to that? Number 2 what do you say?

R: Number 2, eee I have even forgotten the question haahaha.

I: we are talking about the time you receive your results, after giving out your sample for viral load. When do you receive your results?

R: Number 2, Your get your results depending on the type of medication you were given in that month. For example, if you are given medication for 3 months, when those 3 months are up and you have finished your medication and you come back for another dose, that is when you get your results back. If the doctor forgets you remind him that you are supposed to give me my results and if he remembers he will just give you your results straight.

I: okay thank you participant number 2, maybe is there another participant to add to that, welcome participant number 9.

R: Number 9, like participant number 2 said that the date you give your sample is comparable to the date you will come and take your results when you are coming to take your next dose of medication. When you come to take your medication is when you will get your results, and if the doctor forgets you will remind him that I did the test on such and such date and he will give you your results.

I: okay okay, now I would like to know from you, how Is this test done and which approach is used to give your results after the test? Welcome participant number 4.

R: Number 4, the results are given to you on the day you come to take your medication. When you go in to see the Dr you find that he has already written your results in your file. He reads the results to you maybe viruses are 100 or 200, are asleep, depending on your results on that day. There are no results that are given by phone.

I: Welcome number 7 you raised your finger.

R: Number 7, we get our results on the day that we come to take our medication. You are given your results and are told how you are doing, your viral load, he tells you everything then and then, and if there is anything lacking the doctor counsels you.

I: Okay, okay we have heard participant number 7. Welcome participant number 6

R: Number 6, it is like my fellows have spoken, the day you are scheduled to come and take the next dose of medication you are supposed to ask the doctor for your results. I know that the doctor can write them on your card but others do not follow-up and cannot read what is written in the file/card. So as a client you must ask because if you do not ask …………

I: so the results are documented in a card?

R: Numbe 6, I usually ask, I ask the doctor and that is when he gives me my results.

I: What about others, do you find your results already written in your card? Welcome number 5.

R: Number 5, they may be written in your card but you may not know. But what I used to do when I get to the clinic is first, I ask for my results if they are out, because I know I took the test on a previous visit, So he review file to check and share the results if available.

I: thank you very much, is there anyone else who would like to add, welcome number 1.

R: Number 1, No

I: welcome number 2

R: Number 2, they do not call us to give us our results but when a client comes to take medication in his clinic is when you are given the results and other times I ask if my results are ready so that they give them to me. So if they are ready the doctor will open the file and give you your results and he tells you that your results are out and your viruses have increased or are asleep.

I: thank you very much. So you have all said that when you come for your medication in your next clinic is when you are given your results, thank you very much. After answering all those questions, I can see there is a rain challenge. But I would like to know what made the access of your results easy or if there was any challenge in getting back your results? Welcome participant number 4.

R: Number 4, what makes receiving the results easy is because the same day you come for your medication is the same day you get your results. So you do not come to the clinic one day to get your results and another day to take your medication.

I; okay, okay, thank you very much, you have heard participant number 4. Welcome participant number 2.

R: Number 2, what makes getting the results easy is that on the same day you take your medication is the same day you get your results. The results are written in your file so on the day you come for your results the nurse looks in your file and gives you the results.

I: thank you very much, welcome number 8.

R: Number 8, on my side during CORONA they once called me.

I: thank you very much, can you tell us what was the reason for the call during CORONA?

R: Number 8, the service providers called me to ask me where I was and they came to serve me there.

Question number 7

I: Now let’s finish up, let’s look at the issue of education. What can you tell us about education concerning HIV viral load testing, how do you get this education concerning HIV viral load testing? And the education you get how is it given, first is the education even there? Welcome number 6.

R: Number 6, that education used to exists and they used to educate us in the past but these days it is not there anymore.

I: thank you very much, so the education used to be given in the past, right?

R: Number 6, Yes but now it is not there.

I: okay thank you, welcome number 4.

R: Number 4, Education used to be there because this viral load test came after CD4 test. So in the beginning clients were given education and when the time comes to do the test a client comes because he already has the knowledge on it.

I: thank you participant number 4, participant number 2 welcome.

R: Number 2, Education is given when you first start clinic, that is, when you are new to the service that is when you are given education from time to time but when they perceive you have enough experience in the service, they do not give you education anymore.

I: Thank you, we have participant number 2. Number 1 what do you say concerning education?

R: Number 1, Education used to be given when a client just starts clinic, for example if a client just started clinic and he has 1 or 2 months you are given education often about your health. But once you have attended clinic for a year the service providers see that you are experienced so they do not educate you anymore.

I: okay, and what about education on viral load I wanted to know concerning this test. Are you given that education? Welcome participant number 7.

R: Number 7, About viral load test? To be honest, I have not yet got that kind of education.

I: Eee number 9 what do you say concerning education on viral load?

R: Number 9, we are given education, we are educated on how we should continue to use the medication so that viruses do not increase.

I: okay, so that education which you said was given in the past what kind of education was that and how was It given? Number 1, welcome.

R: Number 1, When you come for clinic, they tell you to be careful with unprotected sex so as to reduce the infection. Because when we test for viral load and it exceeds 1000 it means the client is in a bad condition. So the service providers educates you to be careful with unprotected sex.

I: thank you so much, welcome participant number 6.

R: Number 6, the education they share is all about to adhere medication, the second is to eat well and balanced diet, and the third is to avoid unprotected sex.

I: thank you participant number 6. Participant number 5 would you also like to add something?

R: Number 5, my fellows who have spoken before are correct. We are given education to prevent new infection.

I: okay, okay, so in that education, are you also taught concerning viral load testing? Number 5

R: Number 5, Yes we were taught on the importance of testing and protecting ourselves from new infection.

I: okay, okay, and how often is that education given maybe? Number 6 welcome.

R: Number 6, on my side I’m not aware if there is any specific day for health education. And that is why I told you that in the beginning we were encouraged by such things, but nowadays I have not received such education in a long time. Before, it was that when you come for clinic you find the service providers seated there and they tell you take a seat and talk about health education.

I: Okay

R: Number 6, so there is no specific day maybe Saturday or Sunday or Monday. I have never known such a thing, maybe if that tendency is there but I have not known.

I: okay, okay, thank you very much. We are looking at education on viral load testing. Welcome number 2.

R: Number 2, Yes, just as the brother said, in the past it was that before taking medication first the service providers would tell us seat and let us educate you, but now days it seems they have reduced giving that education. So when you come for clinic what the service providers care about is serving you, giving you your medication and you leave but because we are older in the service and we know what to do to protect ourselves from new infections.

I: okay, okay, welcome someone else so we can wind up, welcome number 9.

R: Number 9. Education has been given when we come to take medication and in the past it was daily when you come for clinic before the service providers start giving medication they first give education. They educate us on how to use the medication, how to protect ourselves, and after giving us that education is when they continue with giving medication. But it has been a while, it is like they are complacent about doing it. We ask that they do not stop giving us that education taking it that we have understood. We ask that they continue giving us that education from time to time.

I: thank you very much participant number 9 we have heard his views. Now let’s look at the conditions in which education is given. For example, in the times when they were giving education here, was it the same time when you come to the clinic? Welcome number

R: Number 3, There isn’t a special day.

I: Welcome number 5, so is it when you come to the clinic that you get education?

R: Number 5, There isn’t a special day because if you schedule a client to come just to get education he will not come. But if a client has come to get medication on his clinic day it is easy to educate him.

I: when we look at the issue of advertisements, are there any advertisements that explain about viral load testing? Are there any adverts, banners or fliers posted at your clinic that educate on viral load testing? Number 1 welcome

R: Number 1, there are no fliers or adverts/posters concerning HIV viral load testing.

I: thank you participant number 1, welcome participant number 5, what do you say?

R: Number 5, there are no advertisements

I: Number 8 what do you say?

R: Number 8, There are no fliers or adverts about viral load.

I: Number 6, welcome

R: Number 6. On my side I say that adverts are there but there are no fliers, because I usually read them on the walls but there are no fliers I have never seen any.

I: Participant number 4 is there something you would like to add?

R: Number 4, Adverts are there but there are no fliers.

I: Thank you very much, now I would like to know are you given a chance to ask questions, to contribute your views when you are given that education on HIV viral load testing? Welcome participant number 9.

R: Number 9,We are given the chance because once the service provider is done teaching he usually asks if there is a client with a question, or anyone with a contribution. So they do give us a chance.

I: okay, participant number 7 what do you say?

R: Number 7, We are given a chance to ask questions

I: Welcome participant number 2.

R: Number 2, Yes, I am given a chance to ask questions.

I: and In asking those questions, do they give you the answer at the same time or do they answer you later? For example, maybe right now we have spoken of a certain issue concerning your health, if you ask a question now do they answer right now or later? Welcome participant number 9.

R: Number 9, We are given the answers right there and then because when we ask those questions there more than one service provider, sometimes there are 2, other times there are 3. So they help each other in answering the questions we have asked.

I: Okay, participant number 9 said that he gets the answers, it is like a discussion, he asks questions and he gets the answers then and there. Thank you very much, now this education that you have gotten, did it help you? How much has it helped you? Welcome participant number 4.

R: Number 4, the education we get on viral load testing helps us a lot because we can know our health progress, to know CD4, to know if the viruses have reduced or if you need to adhere to your medication. Also know that I should continue to use my medication at the right time, so I enjoy a lot of advantages because of the tests.

R: Number 2, the education that I got helps me because in the beginning when I came for the test and I got the results that I am infected you have a really different mind but through the education that you get you learn a lot of things about the disease, you continue to follow the rules and until now I am doing well and life goes on.

I: thank you very much participant number 2, welcome someone else to add to that. Welcome number 6.

R: Number 6, Education has really helped me when you are given education and you adhere to medication, you follow the allocated time properly for taking your medication. So if you follow what you have been taught even the results that you will get after testing will be satisfying. So the education we are given is good and it brings hope to us.

I: thank you all because you have known that the education that you are given helps you. And maybe who are those who give you this education? Welcome participant number 9.

R: Number 9, Education is given by those service providers who serve us, those who give us medication, take our blood samples for testing, all of them help each in educating us.

I: when the service providers are educating you what language do they use, do they use a friendly language that is easy to understand while they are educating you? Welcome participant number 2.

R: Number 2, they use a friendly language because they usually tell us, “I have the same status so do not be afraid” so as a client I am encouraged and I say, so even the service provider is like me, I better take my medication so that I can be like him. So you have peace and are happy.

I: Okay, thank you, anyone else? Welcome number 9.

R: Number 9, the language used is good but there are times if there is something to emphasize on they would emphasize it and say if you complacent in following the instructions of taking the medication there are going to be negative effects. So they have been using a good language and it also builds us to know that if we do this it will result in this and if we do that it will deter our health.

I: thank you very much number 9, welcome someone else to finish up, Number 4.

R: Number 4, the language used is good, it is fun it motivates and it is generally good.

Question number 8

I: okay, thank you very much, we would like to get your views now, what should be done in order to improve HIV viral load testing at your center? What thing do you think that if this is done it can improve this service? Let’s start at this center, then we will move to as a district then as a country. Number 7 welcome.

R: Number 7, To expand the area we get this service in our hospital. If there can be a room for the person who asks clients’ views or problems that clients have, we are asked but when we are in a group. but in public a person cannot say his secret problems in front of many people. So when you are asked by a service provider if there is anything bothering you, you will say you do not have a problem but in your heart there is something bothering you. This is a big problem here at our center. So the area for giving service should be expanded.

I: okay, okay, participant number 8 what do you say, what should be done at your center now?

R: I would add on the area of availability of medication. I would advise that they should add this medication for constipation because when we use these medications for a long time they bring us some effects. For example, our fellows in Uganda are given these for free.

I: okay thank you very much, welcome number 9.

R: Number 9, there is something I would like because there are things that we clients do not wish to be in public, we need that our area for getting service to be private. For example when we were brought here we were very happy when we were removed from an open area, but later they came and added people to us. When we were alone we felt like we are hidden and we did not want other people to know because there are people who know us.

I: Okay

R: Number 9,So if someone who knows you sees you there he knows what is going on. We have become lonely because of our news being spread around. So we were asking if they could cover this clinic area so that we are not seen by other people who came to get other services. So we are asking that our area be isolated so that we receive our services by ourselves.

I: welcome number 6, but also let us look at viral load testing.

R: Number 7, I would like to also remain in the first question that my fellows talked about but I would like to emphasize on one side that you sometimes as clients we are told that these cards are the ones that ID us. Now you can find that I came to get service and I have a problem I can not come to this side, I am told to go to the other side and it becomes a problem. My suggestion is that that if possible we should be put on this side, not that we have to go to the other side and show our cards first. This would also help to some extent.

I: okay, so improvements should be made on this side.

R: Number 7, Yes, not that you are told to go to the other side and show your card. That side has many people.

I: thank you very much, now what else should be done? Number 3 you have been very quiet, please tell us what should be done so as to improve HIV viral load testing? Number 3? Hahaha number 3 seems to be at a loss. Welcome number 1, number1 is just smiling. Now let’s go to the side of a district, welcome number 7.

R: Number 7, on the district side my thoughts are if all clients would be served as our fellows who get donations for nutrition from abroad, nutrition and food and other things that clients use.

I: thank you participant number 7, welcome participant number 9.

R: Number 9, something that I wished that they would consider also, some of us are small enterpreneurs who have a limited ability to take care of ourselves in terms of food, that is like getting food that is good and will give you the strength and good health you need, because of our limited ability we fail to get it. But if the government could help us as a district and support us with small loans. Because we can manage very small loans, that will not have very high return interest. This would really help us.

I: thank you very much, we have heard number 9. Now as a country what do you think should be done? Welcome number 9.

R: Number 9, People should continue to be educated and the emphasis should be added so that people can know what to do incase they are infected. And there are people who have not gotten this education and they are living without knowing that there is this disease and others are living carelessly. But if education is given often, it will help because people will know that this problem exists and they will know how to deal with it.

I: thank you very much, welcome participant number 6.

R: Number 6, I agree with participant number 9, education should be emphasized and continue to be given because you know those of us who have this condition, there are some who are in the government, they should help us to give this education on prevention, and its effects. So I ask that they continue to educate us so that it can help us.

WHY IS IT THAT SOME CLIENTS DO NOT WANT TO TAKE THE VIRAL LOAD TEST?

I: okay, okay, thank you for your good views. Now among the answers we got from your service providers that we interviewed on the challenges they face , they told us that some clients when they are scheduled for the viral load test they do not show up. That is one of the issues they raised, now we would like to hear from you, is it true or not? Number 9 welcome

I: Number 9, thank you very much. There is some truth in it, and the truth is when you find that the client who is scheduled to come for testing forgets the date for testing.

I: okay, okay

R: Number 9, and if you remember, in the beginning we said that as much as the service providers call us to remind us that helps us to come for the service on time. And sometimes it happens that the client gets an emergency or problem and has travelled and the scheduled date finds him on the journey. So it becomes a challenge to the client when he comes back to the clinic to take his medication.

I: okay, okay, thank you participant number 9, number 6 you also raised your finger, welcome we are listening.

R: Number 6, I have the same views as my sister there.

I: it seems that when you are scheduled for the test you do not come.

R: Number 6, let me tell you something, if you know who you are

I: Yes

R: Number 9, when you have an understanding and you have accepted yourself, that is the major thing. So even the time you are scheduled to take the medication, if you accept it, and value it, you will not fail to come for clinic. If you value yourself you will manage your time, you will take your medication on time, for example I live in KImara, and I have recognized the importance of getting this service so I will wake up very early in the morning to come for the service even if I decide to walk I will still come and I will have know myself. So there are some clients who have not yet understood the importance of this service. You find that a client forgets his appointment while you already knew the date you were given to come to the clinic for the test. Because you forget the service provider who calls you to remind you reminds you for your health. So the first thing is to value yourself.

I: thank you very much, let’s finish there. Welcome number 4

R: Number 4, there are some clients who are complacent and there is another one who knows that today is the date for sample taking but he avoids coming because he is just scared of giving blood.

I: thank you very much, number 2 welcome

R: Number 2, you can find that a client has been scheduled on a particular date but when the date arrives you find that you have other activities and you fail to come and you decide to come on another date. This contributes to distorting the service providers’ schedules because they had already scheduled you but you did not show up. So on the day you decide to come they will tell you to wait as they take samples from those who are on the schedule first and when they are done they will take your sample.

I: thank you very much. And if a client comes on a day he was not scheduled does he still get the test? Welcome number 4.

R: Number 4, when you come on a day you were not scheduled you will get the test but you have to accept that you will get it late. It is until the clients scheduled for that day get the test then when they are done they can take your sample. But it does not mean you will be the last one in the clinic to be attended, no, but you will have to wait for those on the schedule to take the test for that day to do so and then you will follow.

I: okay, okay, then I want to thank you for your views and as I told you in the beginning, they will help us know what needs to be done to improve the service of HIV viral load testing. Like we said it seems there is a challenge for some of the clients failing to get the test. Okay eee. So I thank you very much, is there anything anyone would like to add in line with what we have been discussing? Welcome number 6.

R: Number 6, on my side I think that you have interviewed as very well and everyone has given his contribution. But I want to emphasize first you who interviewed us or I should ask when you take views from people

I: Yes

R: first all these things we have told you should be worked on and also I think there is a reason why you came all the way here to interview us

I: Yes

R: but what is the main aim you had for coming to question us because I know you have been sent. What evaluation are you doing to make sure that what we have shared will be implemented? We are here and money is a challenge we have and you have come to take our views, so how will you solve such challenges?

I: thank you very much for a good question. Like I said from the beginning we need your views inorder to make changes in some of the services especially viral load, okay. That is the main aim because it seems that many clients do not take the viral load test. Now we want to know why and you have given us the reasons, ok. So my colleagues and I will take these views and work on them. In the discussion that we had we have seen 1,2,3, okay, for example the issue that some clients do not want to come and test like you said, some are afraid to give their blood. Now what do we do about clients like those, so we will seat as a committee and discuss it that we have found this challenge in a certain center, and then maybe we have found it also in another center so what should we do about this issue. What methods should we use so that they can still do the test because they have a right to do the test, because if we do not address it those clients will continue to get more problems. We also look at the issue of the environment where these services are given, what should be improved in those services. We will go and work on these issues and this is the main aim. Like we told you in the beginning. We cannot just rush to go and make changes if we do not know for sure the changes are needed. So these views will really help us and let me assure you that what we have spoken is for my ears, yours and the ministry and it will not be spread else where and we will work on them as we have been instructed. So you can be surprised that in 2022 things have started slowly changing then you know it is your views and suggestions working, because it is all your right to get the viral load test. So inorder for us to know what to do we have asked you about adverts, education. So we are going to emphasize on those very things that education should be given, service providers should do so and so. Okay eee. So I thank you very much for giving your views I donot know if I have answered appropriately.

R:Number 6, I have asked you this because it is not my first time seating for such an interview, we have been interviewed before and they told us that they want to bring the services into our homes then they call you and they give you your package.

I: Yes

R: Number 6, it once was done in that way but it did not continue.

I: Okay, okay

R: but the second thing is the question the lady there asked that there are times that a client can not come to the clinic, you do not have the strength to come but they say she can not get the service until she comes herself and does the test. I think you can see that this is an issue and it should be addressed.

I: okay, okay, thank you very much, and we will work on it because when it comes to the test the client himself must be present so as to do the test, this is a must because you can not send someone to do the test for you, that is the first thing. When it comes to the test, that is viral load test the client has to be present himself, but when you have a challenge like the lady number 9 said, you can ask but it depends on how the service provider will receive it. That I was supposed to come and take the test on a certain date but I failed to come because of certain challenges, for example our sister there said she was sick but it depends on the service provider who received the complaint. So it depends on the service provider and her reception of the situation, it depends on her humanity to think that this is our client who has never missed a clinic but she is facing a challenge how do we help her? That depends on the person’s heart. Welcome number 9.

R: Number 9, I think for a person who was supposed to do the test but is sick, it is not something she wanted so the government should look at this, sometimes they should help us. They can even send a service provider after his work to go and serve the client because the test time has no edn

I: Yes

R: Number 9, once she is done serving those other clients, even if it is at 4pm she can go for that client and take her sample then come back and continue with work. This will help because you donot know the condition of that client. But you find that the service providers say you must come, how can you ignore a test…. That is not a good answer to a client and it discourages us.

I: Thank you number 9

R: Number 6, I think she has said it all because I wanted to say the same thing. How do you help such a client? But she has already talked about it.

I: Thank you very much, number 5 I think you also faced this.

R: Number 5, Yes and it really hurt me.

I: Okay. So I just want to thank you for your time and we have come to the end of our discussion today and I thank you for being patient with me even thought it was raining and we have finished on time. So I wish you a good day and good work.

R: All, Thank you

I: okay.
