# Supplementary material for: Patient reported experience measures on HIV viral load testing at public health facilities in Dar es Salaam, Tanzania: A convergent mixed method study
Source: PLOS Glob Public Health. 2023 Apr 7;3(4):e0001024. doi: 10.1371/journal.pgph.0001024 (PMC10081772; doi:10.1371/journal.pgph.0001024)
Supplement: S1 Appendix — (DOCX) [file pgph.0001024.s005.docx]

FOCUSED GROUP DISCUSSION INTERVIEW GUIDE.

(Intended for adult HIV patients who are active in care for at least a year).

My names are ………………........................... I am a physician doing research to understand the facilitators and barriers of HIV Viral Load (VL) testing from your experience. I’m here to conduct this research which I’m ethically supported by the Muhimbili University of Health and allied sciences (MUHAS) and the Northwestern University (NU), United States of America. Thank you for being willing to speak with me, so we can learn more on patient’s viral load (VL) testing from you as patients. Everything you tell me will be important and useful to inform how we can improve the uptake of VL testing in the country. Our discussion will be guided by the important areas we have identified as challenges or helpful for VL testing and will last for 30-60 minutes. There are not right or wrong answers-just your experience and opinion. The information that you will provide will remain confidential and will only be accessible to our study team. Nothing you say will be shared with your providers and your responses will not affect your care in any way.

Do you agree to the interview? _______yes _______no

We request you to allow use the tape recorder with the aim of improving the correctness of the information when writing the report. After we complete converting the recording into writing, we will destroy the recording and your name will be removed from all documents

Do you agree to the interview? _______ yes ___no

If YES, please sign the consent provided in a separate sheet.

Do you have any questions before we start?

As we are starting our discussion may you kindly introduce yourself (age, marital status, duration in the HIV care, source of income, education and residence)

| **ID** | **Age** | **Sex** | **Marital status** | **Education** | **Source of income** | **Duration (years) in care** | **Residence** |
| --- | --- | --- | --- | --- | --- | --- | --- |
| 1 |  |  |  |  |  |  |  |
| 2 |  |  |  |  |  |  |  |
| 3 |  |  |  |  |  |  |  |
| 4 |  |  |  |  |  |  |  |
| 5 |  |  |  |  |  |  |  |
| 6 |  |  |  |  |  |  |  |
| 7 |  |  |  |  |  |  |  |
| 8 |  |  |  |  |  |  |  |

1. As you are all enrolled into the CTC, may you kindly tell me the type of services that you are supposed to receive from this clinic? (Probe mediation/consultation, monitoring of vital signs, CD4 counts test, drug refill & viral load testing)

1. Among the services that you have mentioned include VL testing. Let’s start by asking what you understand about HIV VL testing, who volunteers to start? Tell us.

Prompts:

- 1. What does it involve?
  2. What is its importance?
  3. How often should it be done?
  4. Who usually requests VL testing in routine care (Dr vs patient or other)
  5. What are the reasons of monitoring viral load?

1. How do you think getting the viral load testing has been important for your HIV care?

**Probe:**

1. If important why? Change treatment, know doing well?
2. If not important, why not? What would make it more important?
3. For those of you who have had a VL test, can you share with us on your experience of your interaction and communication with the care provider in previous VL test?

(REPORTED EXPIRIENCE MEASURES)

Prompts:

- 1. How well did you understand what they told you about the test? What is provided in a language that you easily understood? What were challenges
  2. Being treated with respect? Respect means being treated politely and fairly
  3. How well did the doctors and nurses or other health care providers listen to you.
  4. Being given a chance of ask questions and clarifications
  5. Were you given sufficient responses to what you ask/requested for?
  6. What would you like the care providers to improve?

1. Was it easy or hard for you to get VL testing when you were told to do one?

Prompts:

- 1. What were challenges
     1. Costs?
     2. Distance
     3. Health care worker don’t communicate the test appointment?
     4. Drug refill being different from test visit schedule? What else?
     5. Too long wait?
     6. The available Lab not able to do it?
     7. Lab technician not available to take the specimen?
     8. Lab supplies were out of stock?
  2. What has worked for those who got testing-what made it easier? What were things which helped
  3. Ask if there is anyone who has not tested VL in the past 1 year. Let the patient explain what were the reasons for not testing.

Probe: What was barriers

.

Was COVID 19 a challenge?

1. How long does it take for you to receive back the VL results?

Prompts:

- - 1. Was such waiting time for the results okay Why?
    2. How did you get results? (in person, by phone)
    3. How well were the information about the results explained to you?

What made it easier to understand? If it was hard to understand what were the reasons (probe on language used? Poor interaction with care providers or no one was available to ask?

- - 1. If you did not get results-what were the barriers?

1. Can you tell us about health education on viral load test and results? How do you receive education on VL test?

Prompts:

- 1. What kind of education on VL testing have you received or seen? How often have you gotten information in the last year? Ever”
  2. Probe on availability of the health education environment (during clinic session, is there posters/leaflets, any methods like use peer educators etc.)
  3. The friendliness of the language used during the education session
  4. Are you given an opportunity to ask questions during the education sessions?
     1. If so do you receive prompt responses?
  5. How helpful is the education provided?

1. In your opinion, what do you think should be done to improve the rates of VL testing at this facility?

Prompts:

- 1. Probe “what else” if seems to stop listing the points
  2. What do you think is needed at a city or national level?

**Conclusion:**

Thank you for your time and participation in this study.
