# Supplementary material for: Patient reported experience measures on HIV viral load testing at public health facilities in Dar es Salaam, Tanzania: A convergent mixed method study
Source: PLOS Glob Public Health. 2023 Apr 7;3(4):e0001024. doi: 10.1371/journal.pgph.0001024 (PMC10081772; doi:10.1371/journal.pgph.0001024)
Supplement: S2 Appendix — (DOCX) [file pgph.0001024.s006.docx]

**PATIENT REPORTED EXPERIENCE MEASURES (PREMs) SURVEY TOOL.**

**(Be conducted to a consented HIV patient who met the study criteria indicated in the patient information sheet and the protocol)**

**Patient Initials _______________**

INITIALS

**Study ID ____________________** ID

**Hospital Registration Number: ___________________**

HOSPREG

**Date: __ __/__ __/__ __ __ __ (dd/mm/yyyy)** DATE

**Start time __ __:__ __ (24 hour clock)** START

**Introduction:**

**Personal Information:**

1. What is your current age in years: ______ (number) AGE
2. What is your sex [check one] SEX
   1. *1. Male*
   2. *2. Female*
3. What level of school have you **completed?** ED
   1. *1. Less than primary school*
   2. *2. Incomplete primary education*
   3. *3. Completed primary education*
   4. *4. Incomplete Secondary education*
   5. *5. Completed Secondary education (O or A level)*
   6. *6. University/College or higher*
4. Which district do you live in most of the time?: ____________ DIST
5. How would you describe where you live? HOME
   1. *1. I or my family own the house*
   2. *2. I stay in a rented house*
   3. *3. I have no regular place to stay*
   4. *4. Othe*r (*describe) ________________*

**Socioeconomic status of a family.**

1. Does your household have: HOUSE
2. *Electricity: 1. Yes ____ 2. No ___*
3. *A television: 1. Yes ____ 2. No ___*
4. *A radio: 1. Yes ____ 2. No ___*
5. *An iron: 1. Yes ____ 2. No ___*
6. Does any member of this household have a bank account: 1. Yes ___ 2. No ___ BANK
7. What is the main material of the floor of your dwelling? FLOOR
   1. *1. Earth/sand/dung*
   2. *2. Cement/concrete*
   3. *3. Other*
8. What is the main material of the exterior walls of your dwelling? WALL
   1. *1. Cement blocks/bricks*
   2. *2. Other*
9. What is the main material of the roof of your dwelling? ROOF
   1. *1. Iron sheet*
   2. *2. Grass/palm*
   3. *3. Other*
10. What type of fuel does your household mainly use for cooking? FUEL
    1. *1. Firewood*
    2. *2. Charcoal*
    3. *3. Electricity*
    4. *4. Solar energy*
    5. *5. Gas*
    6. *6. Other*
11. What is the main source of energy for lighting in the household? LIGHT
    1. *1. Electricity*
    2. *2. Battery/solar power*
    3. *3. Kerosene*
    4. 4. *Other*
12. What is your current employment? EMPLOY
    1. *1. Employed*
    2. *2. Self employed*
    3. *3. House wife/ House husband*
    4. *4. Unemployed*

**Visit Characteristics**

1. When did you start attending this clinic? VISTDUR
   1. *1. Less than 6 months*
   2. *2. Approximately 6 months*
   3. *3. More than 6 months but less than a year*
   4. *4. More than a year*
2. Who recommended or told you to come to this clinic? WHOREC
   1. *1. I decided to come on my own*
   2. *2. Someone from my family*
   3. *3. I was referred by a clinic or from a hospitalization at this hospital*
   4. *4. I was referred from another hospital or clinic*
   5. *5. Other*
3. What type of visit are you having VISTYPE
   1. *1. Routine scheduled*
   2. *2. I have a new problem (Unscheduled)*
   3. *3. Drugs refilling*
   4. *4. Other*

**Clinical Characteristics**

1. Have you ever tested for HIV viral load? VLTEST
   1. *1. Yes*
   2. *2. No*
   3. *3. I don’t know*
2. Have you tested for VL in the past 12 months?
   1. *1. Yes*
   2. *2. No*
   3. *3. I don’t know*
3. If the answer for question above is No, what were the reason communicated to you?
   1. *1. Didn’t get any information*
   2. *2. VL test kit unavailable*
   3. *3. My previous VL test was undetected with no clinical failure symptoms*
   4. *4.Other………………………………*

1. If tested record the most recent Viral load (copies/ml) if applicable
   1. *1. Date sample collected*  /  /  *SVLDATE*
   2. *2. Results*  VLRESULT

1. Indicate patients ART regimen ART_reg
   1. *……………………………………………*
2. ART adherence status (Take a look in the patients file) ADH_Status
   1. *1. Good*
   2. *2. Satisfactory*
   3. *3. Poor*

**KAP**

1. What is your understanding on VL test? VL_UND
   1. *I don’t know*
   2. *A test to know the number of virus in my body*
   3. *A test to know the ARV concentration in my body*
   4. *A test to know the CD4 count in my body*
   5. *Other…………………………*
2. If VL results comes out you are having high number of viral loads, what does this mean to you? HIGH_MEAN
   1. *I don’t know*
   2. *The treatment is doing good with me*
   3. *The treatment is failing and I need to see the counselor*
   4. *Nothing wrong whether high or low*
   5. Other……………………………
3. Regarding the test, do you understand when are you supposed to take VL test? WHEN_VL
   - - 1. *I don’t know*
       2. *After every 3 months*
       3. *six months after initial treatment then once every year*
       4. *After every 3 years*
       5. *After 10 years*

**Patient Experience**

1. How long did it take to arrive at the clinic today ARRIVE
   1. *1. Less than 15 minutes*
   2. *2. 15-30 minutes*
   3. *3. 30-60 minutes*
   4. *4. More than one hour*
2. How difficult was it for you to get to the clinic today ARRDIFF
3. *Very difficult*
4. *Difficult*
5. *Easy*
6. *Very Easy*
7. How would you rate your experience of getting treated with **respect** today? Respect means being treated politely and fairly RESPECT
   1. *1. Very Good*
   2. *2. Good*
   3. *3. Moderate*
   4. *4. Bad*
   5. *5. Very Bad*

1. How well did doctors and nurses or other health care providers l**isten** to you? LISTEN

*1. Very Good*

*2. Good*

*3. Moderate*

*4. Bad*

*5. Very Bad*

1. How easy or difficult was it for you to **follow the provider’s advice** for you to test for HIV VL and managing your illness when results were shared? ADVICE
   - 1. *Very Easy*
     2. *Easy*
     3. *Difficult*
     4. *Very difficult*
     5. *no advice given*
2. How would you rate the **turnaround time** of the VL test at the facility before you received back the VL results? That means, the number of days you waited for the results after sample collection? TURN_TM

*1. Very Good*

*2. Good*

*3. Moderate*

*4. Bad*

*5. Very Bad*

1. How much time did you spend with the care provider during VL test service? (consultation time)? ______ (minutes)

TIME_SP

1. How long did you wait before being seen by your HIV VL service provider? _______________ (time in hours)
2. Where did you wait the longest before getting VL test service at the clinic? [tick one] LONGWAIT
   1. *1. Registration*
   2. *2. Nurse station*
   3. *3. Doctor*
   4. *4. Counselor*
   5. *5. Phlebotomy room*
   6. *6. Pharmacy*
   7. 7. Other…………………….

1. Regarding VL test and results sharing. What do you think about the care provider’s **medical knowledge and skills**? [read out options] MED-KNOW

*1. Very Good*

*2. Good*

*3. Moderate*

*4. Bad*

*5. Very Bad*

1. During the last VL test and results sharing, how would you rate **how clearly health care providers explained things to you**? EXPL

*1. Very Good*

*2. Good*

*3. Moderate*

*4. Bad*

*5. Very Bad*

1. During the last time you tested for VL, how would you rate your experience of **being involved in making decisions** for VL testing? DECISION
   - 1. *Very Good*
     2. *Good*
     3. *Moderate*
     4. *Bad*
     5. *Very Bad*

1. During the last VL test service visit, how would you rate the **readiness of the care provider** in providing the VL test? READINESS

*1. Very Good*

*2. Good*

*3. Moderate*

*4. Bad*

*5. Very Bad*

1. For the past 12 months, how has the COVID-19 pandemic affected you on accessing the VL test service at this facility?

*1. Did not affect at all COVID*

*2. Somehow affected*

*3. Moderately affected*

*4. Badly affected*

*5. Very Badly affected*

40. If the answer above is 2-5, how were you affected? HOW

1. *I did not attend a visit*
2. *Lab was closed or out of stock*
3. *The test was not ordered*
4. *Other……………………..*
5. How would you rate your satisfaction with the VL service you received? SATF_VL

*1. Very Good*

*2. Good*

*3. Moderate*

*4. Bad*

*5. Very Bad*

1. Overall, taking everything into account, how would you **rate the quality of VL test service** you received at this facility? QUALITY

*1. Very Good*

*2. Good*

*3. Moderate*

*4. Bad*

*5. Very Bad*

1. Overall, thinking about your entire last visit, please rate how well the VL service you received **met your health goals**. That is, how much did the VL test and results help solve your treatment failure or help you keep on good treatment adherence?

MET_GOAL

*1. Very Good*

*2. Good*

*3. Moderate*

*4. Bad*

*5. Very Bad*

1. Is there anything else you can tell us to help improve your experience with HIV VL test service at this clinic? ANY
